# CRISPR/Cas9 model of prostate cancer identifies Kmt2c deficiency as a metastatic driver by Odam/Cabs1 gene cluster expression

**DOI:** 10.1038/s41467-024-46370-0

**Published:** 2024-03-07

**Authors:** Huiqiang Cai, Bin Zhang, Johanne Ahrenfeldt, Justin V. Joseph, Maria Riedel, Zongliang Gao, Sofie K. Thomsen, Ditte S. Christensen, Rasmus O. Bak, Henrik Hager, Mikkel H. Vendelbo, Xin Gao, Nicolai Birkbak, Martin K. Thomsen

**Affiliations:** 1 Department of Biomedicine, Aarhus University, Aarhus, Denmark; 2 Computational Bioscience Research Center, King Abdullah University of Science and Technology (KAUST), Thuwal, Saudi Arabia; 3 Computer Science Program, Computer, Electrical and Mathematical Sciences and Engineering Division, King Abdullah University of Science and Technology (KAUST), Thuwal, Saudi Arabia; 4 Department of Clinical Medicine, Aarhus University, Aarhus, Denmark; 5 Department of Molecular Medicine, Aarhus University Hospital, Aarhus, Denmark; 6 Department of Pathology, Aarhus University Hospital, Aarhus, Denmark; 7 Department of Nuclear Medicine & PET Centre, Aarhus University Hospital, Aarhus, Denmark; 8 Aarhus Institute of Advanced Studies (AIAS), Aarhus University, Aarhus, Denmark

**Keywords:** Prostate cancer, Cancer models

## Abstract

Metastatic prostate cancer (PCa) poses a significant therapeutic challenge with high mortality rates. Utilizing CRISPR-Cas9 in vivo, we target five potential tumor suppressor genes (Pten, Trp53, Rb1, Stk11, and RnaseL) in the mouse prostate, reaching humane endpoint after eight weeks without metastasis. By further depleting three epigenetic factors (Kmt2c, Kmt2d, and Zbtb16), lung metastases are present in all mice. While whole genome sequencing reveals few mutations in coding sequence, RNA sequencing shows significant dysregulation, especially in a conserved genomic region at chr5qE1 regulated by KMT2C. Depleting Odam and Cabs1 in this region prevents metastasis. Notably, the gene expression signatures, resulting from our study, predict progression-free and overall survival and distinguish primary and metastatic human prostate cancer. This study emphasizes positive genetic interactions between classical tumor suppressor genes and epigenetic modulators in metastatic PCa progression, offering insights into potential treatments.

## Abstract (web summary)

The molecular basis of the metastatic disease in prostate cancer remains poorly characterised. Here, the authors investigate the interaction of tumor suppressor and epigenetic factor genes and highlight the role of Kmt2c deficiency in facilitating lung metastasis.

## Introduction

Prostate cancer (PCa) is the second most common cancer in man and the incidence is continuously increasing^1^. PCa is notoriously heterogeneous, and the progression from an indolent disease to advanced cancer usually takes several years^2–5^. The molecular alteration that drives disease progression is still not fully understood, even though genetic sequencing of patients’ samples and pre-clinical models have revealed essential mechanisms^2,3,6–8^. Genetically engineered mouse models of PCa have displayed gene functions in the development of prostatic intraepithelial neoplasia (PIN) and PCa^9–12^. Intercrossing of mouse strains with specific loss of different tumor suppressor genes in the mouse prostate has shown cross-talks between these factors and accelerated cancer progression. For instance, loss of Rb1 in combination with Trp53 and Pten results in the formation of neuroendocrine tumors at 4 months and loss of Smad4 with Trp53 and Pten forms adenocarcinoma at 4 months with metastasis formation in multiple organs^13,14^. Intercrossing of multiple mouse strains with conditional loss of specific genes in the prostate tissues is time-consuming. However, this can be overcome by combining CRISPR/Cas9 technology with adeno-associated virus (AAV) delivery system. AAV system is used for gene therapy as it has a low risk of insertional mutagenesis and elicits low immune responses, comparing to other vectors^15^. We have successfully developed a strategy to mutate multiple genes simultaneously in the epithelia of the mouse prostate^16,17^. This method allowed clonal expansion of tumor cells under Darwinian selection as seen in human cancer^18,19^.

Extensive genomic sequencing of PCa over the last decade has identified multiple essential loss-of-function mutations. This includes common mutations not only in tumor suppressor genes such as *TP53* and *PTEN* but also in genes, of which functions are less understood in the context of PCa^3,6,7^. For instance, mutations in epigenetic factors such as lysine methyltransferases (KMT) genes are found in a significant amount of samples^3,6^. Mutations in epigenetic factors such as *ZBTB16*, *KMT2C,* and *KMT2D* are often found in advanced PCa, but the impact on PCa of such alterations remains unclear. Similarly, mutation in *STK11* can occur in PCa, and data from Stk11 deficient mice have revealed a possible tumor suppressive function in PCa^20–23^. While germline mutations in *RNASEL* are less common, they have been associated with a predisposition to prostate cancer^24–26^. Moreover, RNASEL has been linked to other cancer types, suggesting a potential role in cancer incidence and initiation^27,28^.

In this study, we aimed to seek the genetic as well as epigenetic basis for PCa progression and onset of metastatic disease. For this, we applied CRISPR to simultaneously mutate five tumor suppressor genes *Trp53*, *Pten*, *Rb1*, *Stk11*, and *RnaseL*, in the mouse prostate by AAV delivery. This generated a rapid invasive and androgen-independent tumor where mice reached humane endpoint at eight weeks after initiation but without the formation of metastasis. Additionally, CRISPR guides targeting *Zbtb16*, *Kmt2c,* and *Kmt2d* were included together with the aforementioned five genes to mutate eight genes simultaneously. The tumor progression was unchanged, but all mice developed lung metastasis, and further investigation revealed that loss of Kmt2c was essential for metastasis formation. With whole genome sequencing (WGS) and RNAseq, we addressed that tumor progression was driven by alteration in gene expression in classical pathways but not by additional somatic mutations. Furthermore, the loss of Kmt2c resulted in the upregulation of a highly conserved region covering a unique gene cluster, which has not previously been associated with tumor development. Remarkably, the disruption of two genes in this cluster, *Odam* and *Cabs1*, dismissed secondary tumor formation, revealing their implication in metastatic formation. Overall, this study showed that loss of multiple tumor suppressor genes accelerated PCa progression and that loss of Kmt2c initiated the onset of metastatic disease through regulation of a unique gene cluster.

## Results

### Loss of multiple tumor suppressor genes accelerates PCa progression

**Fig. 1:**
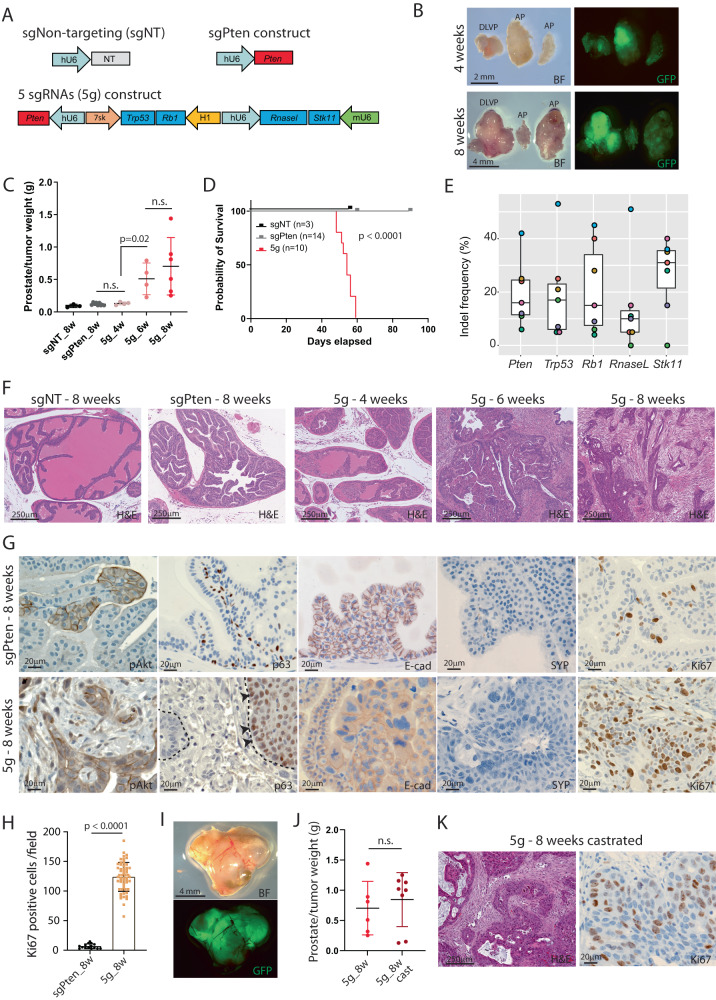
Generation of an aggressive primary tumor by loss of five tumor repressor genes. **A** Three AAV constructs were cloned to generate AAV particles. A construct with a non-targeting (NT) sgRNA, a construct with a sgRNA for Pten, and a construct with sgRNAs for Pten, Trp53, Rb1, Stk11, and RnaseL (5 g). **B** Mice were injected with AAV particles targeting the five genes, and macroscopic pictures were taken 4 and 8 weeks after injection with bright field and GFP channel. DLVP: Dorsal, lateral, and ventral prostate. AP Anterior prostate. **C** The weight of the prostates was measured at different time points after administration of the AAV particles (*n* = 3, 9, 4, 4, 6). **D** Overall survival for the three groups of mice (Log-rank Mantel–Cox test). **E** Indel formation in the tumors at 8 weeks after delivery of AAV particles containing five sgRNAs (*n* = 7, each color represents the same sample. Box plots present minimum-maximum and percentile data at 25%, 50%, 75%). **F** H&E staining of prostate tissues from the three groups at different time points. A representative picture is shown (*n* = 10). **G** IHC on prostatic sections at 8 weeks after the mice received either sgPten or the 5 g construct (*n* > 5, a representative picture is shown). **H** Quantification of Ki67 positive cells in tissue sections from sgPten control and 5 g tumor samples at 8 weeks (*n* = 15, 57 fields, respectively). **I** A group of mice was castrated at 5 weeks after delivery of AAV particles containing 5 sgRNAs and sacrificed at 8 weeks. Images were taken with bright field (BF) and GFP channel. **J** The weight of the prostatic tissues from castrated and intact mice 8 weeks after injection of AAV particles with 5 sgRNAs (*n* = 6, 8). **K** H&E staining and IHC for Ki67 on prostate tissue sections from castrated mice injected with 5 g AAV particles (*n* = 5). Data are presented as mean values ±SD as appropriate.

Whole exome and genome sequencing (WES/WGS) has revealed genomic alteration in a large number of genes that are associated with prostate cancer, including *TP53, PTEN, RB1, STK11,* and *RNaseL*^3,6,7^. Analysis of WES data of 494 PCa patients from the cancer genome atlas (TCGA) and 1013 patients from Memorial Sloan Kettering Cancer Center (MSK) and Dana-Farber Cancer Institute (DFCI) confirmed that all 5 genes are mutated in prostate cancer with the highest prevalence of *TP53*, *PTEN,* and *RB1* (Fig. S1). Furthermore, we observed mutational co-occurrence between these tumor suppressor genes with no mutual exclusivity detected, and the alteration was associated with a worse prognosis (Fig. S1). To evaluate the positive genetic interaction between loss of multiple tumor suppressor genes in prostate cancer progression, we applied CRISPR technology to mutate these five targets simultaneously in the same somatic cell of a mouse prostate. We constructed a viral vector with unique sgRNAs for *Pten*, *Trp53, Rb1, Stk11,* and *RnaseL* (hereafter 5 g), and generated AAV particles for in vivo applications. As controls, viral constructs with only a guide for *Pten* (sgPten) or a non-targeting guide RNA (sgNT) were produced (Fig. 1A). We delivered viral particles to the murine anterior prostate by surgical injection to Cas9-EGFP flox mice, which were bred to the prostate-specific Cre line, PB4Cre, for limiting expression of Cas9 and EGFP to the prostatic tissues. Hereby, cancer was induced specifically to the mouse prostate, and undesired tumor inductions to other organs were avoided.

The weight of the prostate was evaluated four weeks after tumor initiation, and no difference was observed between control groups and 5 g samples. However, this was significantly altered at 6 weeks after injection, where prostates with loss of five genes were enlarged (Fig. 1B, C). At around eight weeks, prostates with five mutations had become very enlarged, and mice were sacrificed as a humane endpoint. In comparison, loss of *Pten* alone or a non-targeting gRNA had not compromised the mice at this stage (Fig. 1D).

To assess if the rapid tumor progression was driven by the CRISPR-induced mutations in the five target genes, biopsies underwent Sanger sequencing at the target site. Mutations in *Pten* were detected for all samples, revealing that loss of Pten is required for initiation of PCa, as shown before^16,18^. Mutations in *Trp53* and *Rb1* were also detected for all the samples, but *Stk11* and *RnaseL* were only mutated in a subset of samples, revealing that clones with different mutation profiles were present as a consequence of imperfect CRISPR/Cas9 generated mutations (Fig. 1E, S2). Histology assessment of the prostates revealed low-grade PIN in sgPten control samples at eight weeks, whereas 5 g samples contained areas of high-grade PIN at four weeks^10^. At 6 weeks, 5 g tumors progressed to invasive cancer, but no metastasis formation was identified in lung, lymph node, or liver before humane endpoint at 8 weeks (Fig. 1F). Immunohistochemistry (IHC) for phospho-Akt (pAkt) revealed increased levels due to the loss of Pten and staining for basal cell marker p63 showed intact basal structure in control samples. However, this feature was lost in 5 g samples at 8 weeks, as seen in human PCa^29^. Tumors were positive for E-cadherin and negative for Synaptophysin (SYP), showing epithelial origin of the tumors (Fig. 1G). Cells positive for proliferation marker Ki67 were significantly increased in 5 g samples compared to sgPten control in agreement with larger tumor burden (Fig. 1G, H). To assess if the rapidly growing tumors will respond to androgen deprivation, mice were castrated 5 weeks after tumor initiation, and samples were assessed at 8 weeks. No difference in tumor size was observed between non-castrated and castrated mice, even though two mice did present smaller tumors (Fig. 1I, J). Histological assessment of the tumors from the castrated mice showed an invasive tumor with a high proliferation index, similar to the samples from intact mice (Fig. 1K). Overall, loss of Pten, Trp53, Rb1, Stk11, and RnaseL in the murine prostate drives tumor formation to invasive cancer in less than 8 weeks, revealing the existence of positive genetic interaction between these gatekeeper genes in prostate cancer progression.

### Loss of epigenetic factors drives metastatic formation

**Fig. 2:**
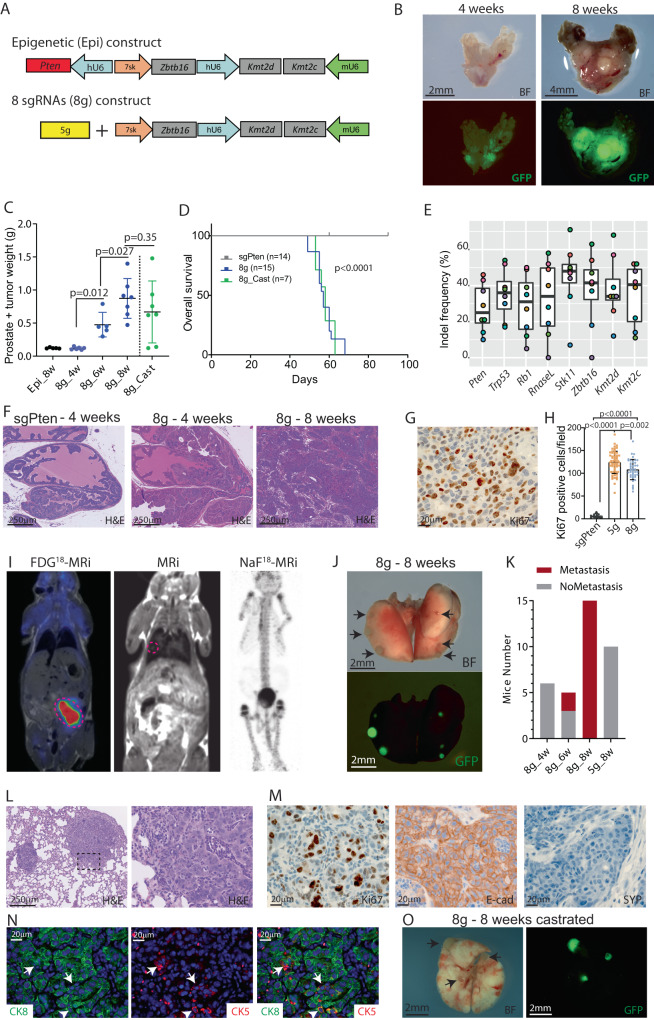
Loss of epigenetic factors facilitated metastasis in the lung. **A** Two new AAV constructs were cloned to generate AAV particles. A construct containing sgRNAs targeting Kmt2c, Kmt2d, and Ztbt16 together with a sgRNA for Pten (Epi). The sgRNAs cassettes for Kmt2c, Kmt2d, and Ztbt16 were added to the 5 g vector, resulting in a construct named 8 g. **B** Mice were injected with AAV particles of 8 g, and macroscopic pictures were taken 4 and 8 weeks after injection with bright field (BF) and GFP channel. **C** The weight of the prostates was measured at different time points after administration of the AAV particles. A group of mice were castrated 5 weeks after AAV delivery (Cast) (*n* = 5, 6, 6, 7, 7). **D** Overall survival for mice reaching humane endpoint for three groups of mice (Log-rank Mantel–Cox test). **E** Indels formation in tumors at 8 weeks after delivery of AAV particles containing eight sgRNAs (*n* = 8, each color represents the same sample. Box plots present minimum-maximum and percentile data at 25%, 50%, 75%). **F** H&E staining of prostate tissues from mice targeted by sgPten or 8 g at different time points (*n* = 10). **G** IHC for Ki67 on tissue sections from 8g-transformed prostates at 8 weeks (*n* > 5). **H** Quantification of Ki67 positive cells in tissue sections from sgPten control, 5 g and 8 g tumor samples at 8 weeks (*n* = 15, 57, 41 fields for each group). **I** PET/MRi scanning of mice injected with 8 g particles at 8 weeks with tracer for glucose metabolism (FDG) and Sodium Fluoride (NaF) for bone metastasis (*n* = 5–10). **J** Images of bright field and GFP of lungs from mice receiving 8 g AAV particles at 8 weeks after injection. Black arrows mark metastases (*n* > 10). **K** Presence of metastasis in lung tissues at different time points from mice injected with either 5 g or 8 g AAV particles. **L** H&E staining of lung metastasis. Black box (left) marks the area in high magnification (right) (*n* > 5). **M** IHC staining for Ki67, E-cad, and SYP on lung metastasis (*n* > 5). **N** IF staining of lung metastasis with Ck8 (green) and Ck5 (red). Arrows mark Ck5 positive basal cells, and arrowheads indicate double-positive cells (*n* > 5). **O** Lungs isolated at 8 weeks from castrated mice injected with 8 g AAV particles. Arrows mark metastasis (*n* = 5). A representative picture is shown, and data are presented as mean values ±SD as appropriate.

Epigenetic factors are frequently mutated in human PCa but their implication on PCa is still not fully understood. Especially, mutations in *KMT2C, KMT2D,* and *ZBTB16* are found in primary PCa and associated with poorer survival, as well as mutation burden for *KMT2C* is increased in metastatic lesions compared to primary PCa (Fig. S3). We sought to understand the implication of epigenetic alterations in PCa progression for these three genes. To this end, we cloned CRISPR guides targeting *Kmt2c*, *Kmt2d,* and *Zbtb16* together with a sgRNA for Pten in an AAV backbone. Furthermore, we assembled the fragments targeting three epigenetic factors into 5 g vector, to mutate eight genes simultaneously in the murine prostate (hereafter called 8 g) (Fig. 2A). AAV particles containing 8 sgRNAs were injected to the anterior mouse prostate, tumor progression was rapid and similar to mice that received AAV particles containing 5 g (Fig. S4). At 4 weeks after tumor initiation the weight of the prostate was not altered, but at 6 weeks a large tumor developed, and ~8 weeks after initiation, mice reached humane endpoint (Fig. 2B–D, S4). Initiation of PCa by the AAV particles containing the epigenetic factors in combination with sgPten did not accelerate tumor progression when compared to only loss of Pten (Fig. S4). Furthermore, mice with loss of Pten, Trp53 and Rb1 had slower tumor progression, revealing that loss of Stk11 and RnaseL accelerated tumor development (Fig. S4G–J). A group of mice was castrated 5 weeks after tumor initiation, and deprivation of testosterone did not impair tumor size, even though two mice had smaller tumors, and expression of the AR-regulated gene Klk4 was downregulated (Fig. 2C, D, S5). Hereafter, sanger sequencing was performed on the tumor samples at the target regions of the guides, to analyze indel frequency. This confirmed that all guides induced indel formations, and most samples had mutations in all eight genes (Fig. 2E). Histological assessment of the tumors showed areas of high-grade PIN 4 weeks after initiation and invasive cancer at 6 to 8 weeks. This was similar to the tumors induced by the injection with 5 g virus but more advanced compared to sgPten control samples (Fig. 2F). Evaluation of the proliferation by Ki67 staining revealed increased proliferation in 8 g group when compared to sgPten controls, but a slight decrease compared to tumors with loss of five tumor suppressor genes (Fig. 2G, H).

The tumor development was monitored by PET/MRi scanning, which confirmed the advanced prostate tumors but also revealed metastatic formation in the lungs (Fig. 2I). To evaluate possible bone metastasis, NaF^18^ was used as PET tracer but without revealing bone metastasis (Fig. 2I). Multiple organs were assessed for metastasis, including liver, lymph node, and lungs. Metastasis was only found in the lungs, but with a penetration of 100 percent at 8 weeks after tumor initiation, and approximately half of the mice had metastasis at 6 weeks (Fig. 2J, K). Histological assessment confirmed secondary tumors of adenocarcinoma, which contained cells positive for basal cells (p63 and CK5) and luminal cells (CK8). Furthermore, the metastasis was positive for androgen receptor (AR) and E-cadherin but negative for SYP and contained a high proliferation index (Ki67 positive), confirming the origin from the prostate epithelia (Fig. 2L–N, S5). The presence of lung metastasis in the group of castrated was unchanged, revealing that deprivation of testosterone did not impair secondary tumor formation (Fig. 2O, S5). Altogether, loss of Kmt2c, Kmt2d, and Zbtb16 in combination with mutations in *Pten*, *Trp53, Rb1, Stk11,* and *RnaseL* in the prostate epithelia resulted in undifferentiated PCa with secondary tumors in the lung six weeks after initiation. This revealed that alterations in epigenetic factors regulate the metastasis potential of the primary tumor.

### Few common exonic mutations in PCa metastasis

**Fig. 3:**
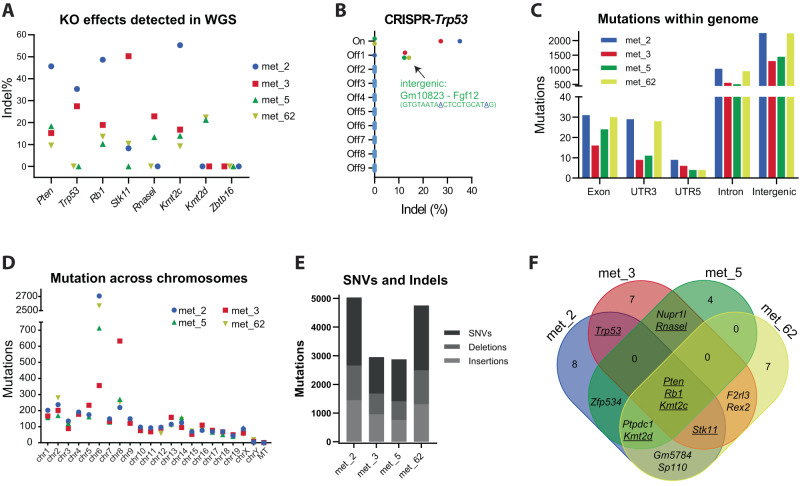
Mutations landscape in metastatic prostate cancer samples. Lung metastases samples from four mice injected with 8 g AAV particles were subjected to whole genome sequencing and mapped to the mouse reference genome (mm39). **A** Indel analysis for the sgRNAs at the specific eight genomic locations targeted by CRISPR/Cas9. **B** Analysis of off-target induced by CRISPR/Cas9 for Trp53 sgRNA. **C** The distribution of genomic alterations was analyzed for each sample and grouped into: exon, UTR3, UTR5, intron, or intergenic regions. **D** Total number of detected genomic mutations based on chromosomes. **E** The contributions of single-nucleotide variants (SNV), deletions, and insertions were analyzed for each sample. **F** A Venn diagram showing either unique or shared exonic mutations across the metastatic samples. Genes with underline were mutated by CRISPR/Cas9 (*n* = 4).

We wanted to investigate if the formation of metastasis in the lung was driven by somatic mutations. For this, four metastatic samples underwent whole genome sequencing (WGS) and were aligned to the mm39 mouse genome. First, we confirmed mutations at the different CRISPR guide targets. Mutations were present in *Pten, Rb1,* and *Kmt2c* for all four samples (Fig. 3A). Mutations in *Trp53, Stk11, RnaseL,* and *Kmt2d* were present in a subset of the samples, while mutations in *Zbtb16* were not detected in any of the 4 samples (Fig. 3A). Next, we performed screening to analyze the potential off-target incidents induced by the CRISPR/Cas9 system. For each guide, we identified the potential off-target sites by CHOPCHOP or tefor analysis tool (Table S1). These sites were assessed in the WGS data and revealed one off-target incident by the Trp53 guide in 3 of the four samples, which was located in an intergenic region flanked by *Gm10823* and *Fgf12* (Fig. 3B).

The WGS analysis showed few mutations in protein coding sequences and the untranslated regions (UTR), whereas most mutations occurred in intronic and intergenic regions (Fig. 3C). The four samples had approximately 1.5 mutations per megabase, equally distributed over the genome, except for chromosomes 6 and 8, which were hypermutated in some samples. The mutations in those two chromosomes were highly over-representative for all 4 samples in both coding and non-coding regions (Fig. 3D and S6). Analysis of the mutation types showed that approximately half were single-nucleotide variants, and the other half were deletions or insertions (Fig. 3E). Besides the CRISPR/Cas9-induced mutations, mutations found in the exonic regions were present in 33 genes across all four metastatic samples (Table S2), of which seven were shared by at least two samples (Fig. 3F). Overall, the WGS data revealed that many additional mutations had occurred across the genome in the metastatic samples, however, few were present in the coding regions, and they were barely shared across different samples (Fig. 3C). This indicates that the specific CRISPR-induced mutations are sufficient to drive cancer formation and metastatic seeding, even though the tumors exhibit genetic instability with a high mutation frequency in intronic regions, resulting in heterogeneous tumors.

### Transcriptome analysis revealed alterations in cancer hallmarks and metabolism

**Fig. 4:**
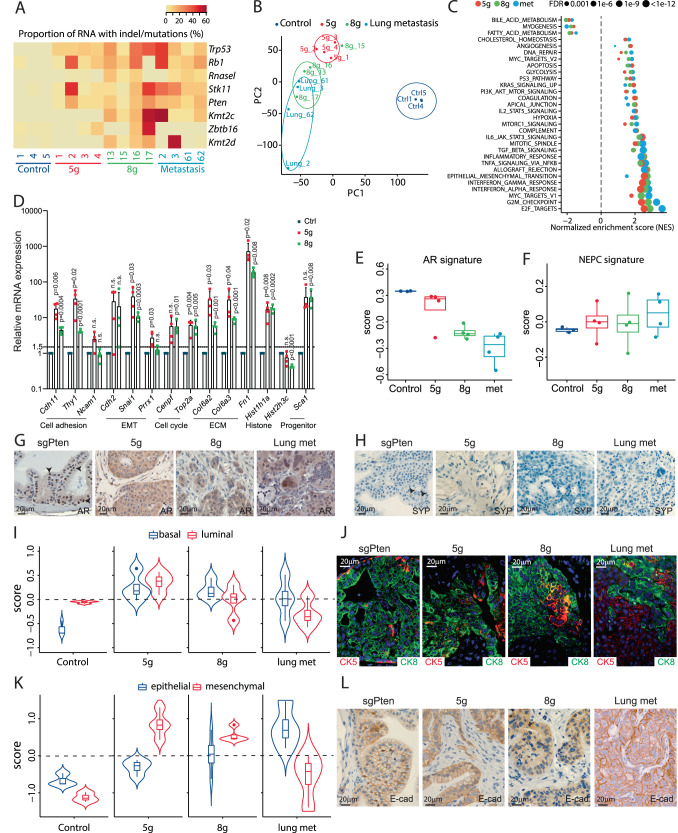
Transcriptome analysis identified alterations in cancer-associated pathways. RNA sequencing (RNAseq) was performed on control prostate tissues, primary tumors from 5 g and 8 g samples, and lung metastases (met) (*n* = 3, 4, 4, 4). **A** Proportion of the abnormal transcripts of the CRISPR/Cas9 targeted genes across all samples. **B** Principal-component analysis of the samples. **C** Pathways enrichment of the dysregulated genes for the three tumor groups compared to control. **D** qPCR on selected genes on RNA from control tissues, 5 g and 8 g primary tumors at 8 weeks (*n* = 4). **E**, **F** Analysis of AR signaling and neuroendocrine prostate cancer (NEPC) signature based on RNAseq data from the four groups. Each dot represents a sample (*n* = 3, 4, 4, 4). Box plots present minimum-maximum and percentile data at 25%, 50%, 75%). **G**, **H** IHC staining for androgen receptor (AR) or synaptophysin (SYP) on prostatic tissues from sgPten, 5 g, and 8 g tumors together with lung metastases at 8 weeks after cancer initiation (*n* > 5). Enrichment analysis for (**I**) prostatic basal and luminal cell signatures and (**K**) epithelial and mesenchymal gene signatures on the RNAseq samples (*n* = 3, 4, 4, 4). **J** IF staining for Ck8 (green) and Ck5 (red), or **L** IHC staining for e-cadherin (e-cad) on prostatic tissues from sgPten, 5 g and 8 g tumors together with lung metastasis at 8 weeks after cancer initiation. Representative pictures are shown (*n* ≥ 5). Data are presented as mean values ±SD as appropriate. Source data are provided as a Source Data file.

To address the molecular mechanism of tumor development and metastasis formation in the model, we performed RNA sequencing (RNAseq) on the primary tumors from 5 g and 8 g groups, together with lung metastasis (met) and control tissues. We were able to detect the abnormal transcripts with indels from the targeted genes, resulting in dysfunctional protein products (Fig. 4A). These results confirmed the mutations identified by Sanger sequencing and WGS, where RnaseL has the lowest mutation frequency in 5 g samples and Zbtb16 was intact in the lung metastasis (Fig. 4A). Next, analysis of differentially expressed genes (DEGs) showed that nearly half of the protein-coding genes were altered in tumor samples, compared to control (*n* = 12,912) (Fig. S7A–C, S8). Principal-component analysis (PCA) revealed that 8 g primary tumors clustered close with the lung metastasis samples derived from 8 g PCa, while 5 g primary tumors and control samples were separated (Fig. 4B). Next, we performed gene set enrichment analysis (GSEA) to identify alteration for the 50 cancer-related hallmarks from the molecular signature database (MSigDB). It showed that multiple hallmark gene sets related to well-known oncogenes or tumor suppressor genes, such as Kras, E2F, p53, and c-Myc were dysregulated in prostate tumors (Fig. 4C, S8). Besides, some common cancer-related hallmarks, such as EMT, cell cycle, apoptosis, hypoxia, and inflammation, were also altered (Fig. 4C, S7D, Table S6). Interestingly, gene sets for metabolism were significantly changed, as observed in human prostate cancer, where protein secretion is stopped, and glycolysis is increased^30^ (Fig. 4C, Table S6). To validate the findings, we performed qRT-PCR analysis on a selected group of genes and confirmed alteration in cell adhesion, EMT, cell cycle, stemness, and histone modification (Fig. 4D).

To define the prostate cancer subtypes, we analyzed the expression data in two gene sets related to AR signaling and neuroendocrine prostate cancer^31–33^. Lower abundance of mRNAs defining AR signaling was found in tumors, which is more evident in 8 g primary tumors and lung metastasis, whereas neuroendocrine signature was unchanged (Fig. 4E, F, S9, S10). IHC staining for AR showed decreased nuclear localization and increased cytoplasmic staining with development of the cancer, whereas IHC for neuroendocrine cells was negative for all tumor groups (Fig. 4G, H, S9). Next, two gene sets related to prostatic luminal and basal cells were assessed across the four groups of samples. The signatures in tumor samples were obviously different from those in the control, and they were also distinctive across the three groups of tumors (Fig. 4I, S11). IF staining revealed changes in the tumors with cells positive for the basal cell marker CK5 distributed throughout the tumor (Fig. 4J). To further confirm the altered EMT signaling, we analyzed two gene sets representing epithelial and mesenchymal cells. This showed highly increased mesenchymal expression in primary tumors but a decrease in lung metastasis (Fig. 4K, S12). IHC for E-cadherin showed transition to cytoplasmic localization in primary tumor, but in the metastasis, the expression was found membrane-bound, suggesting changes in cell fate in the metastasized tumor cells (Fig. 4L). Taken together, these results indicate that multiple cancer-associated pathways are altered in tumor samples, and epithelial plasticity might be a driving force in the primary tumor.

### KMT2C regulates a conserved region of 150 kb in prostate cancer and interferes transcriptional regulation

**Fig. 5:**
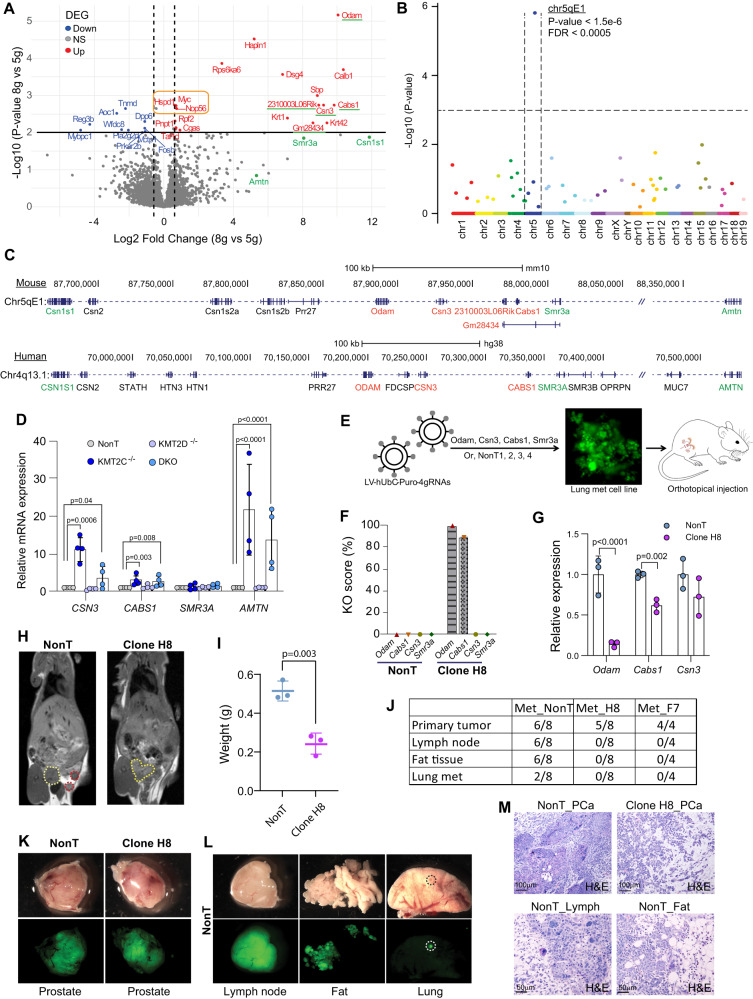
Epigenetic factors altered expression of a unique chromosomal region and driving metastatic formation. **A** Volcano plot showing differential expressed genes between 5 g and 8 g primary tumors at 6 weeks post tumor induction (*n* = 4). Genes underlined in green or names in green were located in a short genomic region. Myc and its targets genes are marked with an orange box. **B** Dot plot indicating the association analysis between chromosomal regions and distribution of DEGs. **C** Gene alignment of the mouse dysregulated region at chr5qE1 and its corresponding region in human, chr4q13.3. **D** mRNA expression analysis for four genes located in the human chr4q13.3 in BPH1 cells. Cells with CRISPR/Cas9 induced mutations in either KMT2C or KMT2D or both (DKO) were analyzed and compared to control cells (NonT) (*n* = 4). **E** Schematic strategy for engineering the mouse Chr5qE1 with four specific sgRNAs in a primary lung metastatic cell line derived from 8 g PCa. As control, a clone transduced with four non-targeting sgRNAs was applied. **F** KO scores for the four genes are shown for NonT and clone H8. **G** Expression of three target genes in the control cells and clone H8 (*n* = 3). **H** MRi scanning of mice 6 weeks after orthotopically implantation of the H8 clone to the prostate. Yellow dotted line marked the primary tumor, and the red dotted line marked metastasis. Representative pictures are shown (*n* = 4). **I** The weight of the primary tumor at 6 weeks after implantation (*n* = 3). **J** Table for formation of primary and secondary tumors by control and two KO clones. **K**, **L** Images of bright field and GFP of primary prostate tumors, lymph nodes, abdominal fat, and lungs from mice receiving either control or KO clone for Odam and Cabs1 6 weeks after injection. Representative pictures are shown; black or white dotted lines marks lung metastases (*n* = 6). **M** H&E staining of primary and secondary tumors at 6 weeks after implantation. A representative picture is shown (*n* = 3). Data are presented as mean values ±SD as appropriate. Source data are provided as a Source Data file.

To investigate the underlying mechanisms that drove metastatic formation, we compared the transcriptome of 5 g to 8 g prostate tumors. Even though we could observe the difference between these two groups of tumors based on the PCA of transcriptome profiles, only 29 significant DEGs were identified, which might be due to the high heterogeneities of samples from the group of tumors. These DEGs include upregulation of c-Myc and two target genes of this transcription factor in 8 g samples, which is known to regulate cell growth, differentiation, metabolism, and cell death (Fig. 5A)^34^. Interestingly, in 8 g samples 5 of the 29 DEGs were significantly enriched in a cytoband chr5qE1, located within a small genomic region of less than 150 kb (Fig. 5B). Moreover, we found that gene arrangements in this region are highly conserved between human and mouse (Fig. 5C).

Since epigenetic factors regulate gene expression by changing chromatin conformation and accessibility with histone modifications and DNA methylation, we speculated that the local chromatin state in this region might be changed in 8 g tumors compared to 5 g. This change was very likely induced by a deficiency of KMT2 factors as they are capable of modifying lysine residues of histone. To test this hypothesis, we generated cell clones with loss of KMT2C or KMT2D and double deficient clones by CRISPR-Cas9 in a benign prostatic hyperplasia cell line BPH1 (Fig. S13). We designed primers to multiple genes located in the region and performed qRT-PCR to assess the expression in the absence of KMT2C and/or D. Expression levels of *CSN3*, *CABS1,* and *AMTN* were indeed significantly increased in clones with loss of KMT2C and double deficient clones. However, clones with loss of KMT2D alone did not alter the gene expression from this region (Fig. 5D). These results showed that dysregulation in this genomic region caused by KMT2C deficiency is highly conserved between human and mice, which might be critical to shape phenotypic difference between 8 g and 5 g tumors.

These facts compelled us to hypothesize that this region may regulate PCa metastasis formation under these conditions. To explore this possibility, we generated a primary cell line from a lung metastasis that contained a mutation in *Kmt2c* but not in *Kmt2d* (Fig. S14A, B). Using CRISPR, we targeted four genes in the dysregulated region and obtained two unique clones with mutations in *Odam* and *Cabs1*. Additionally, a clone with four non-targeting sgRNAs was generated as a control. The cell clones were orthotopically implanted into the anterior lobe of the mouse prostate to establish primary tumors and potentially induce metastasis (Fig. 5E–G). Primary tumors were observed in both the control group and the group with loss of Odam and Cabs1. However, the clones with these mutations had reduced tumor size (Fig. 5H, I). Interestingly, the clones with mutations in Odam and Cabs1 did not form secondary tumors, whereas the control clone disseminated to lymph nodes, abdominal fat, and the lungs of the mice (Fig. 5J–M). Similar results were obtained from a second independent cell clone, revealing that this phenomenon was clonally independent (Fig. S14C–F). A group of mice implanted with the mutant clone was followed for an additional two weeks to allow the primary cancer to reach a similar size as the control tumors, but secondary tumors still did not form (Fig. S14G). Overall, the loss of KMT2D in PCa increased the expression of a conserved gene cluster, which is essential for tumor growth and the formation of metastasis.

### Loss of epigenetic factors enhances p-Src/p-Lyn-c-Myc pathway in primary PCa

**Fig. 6:**
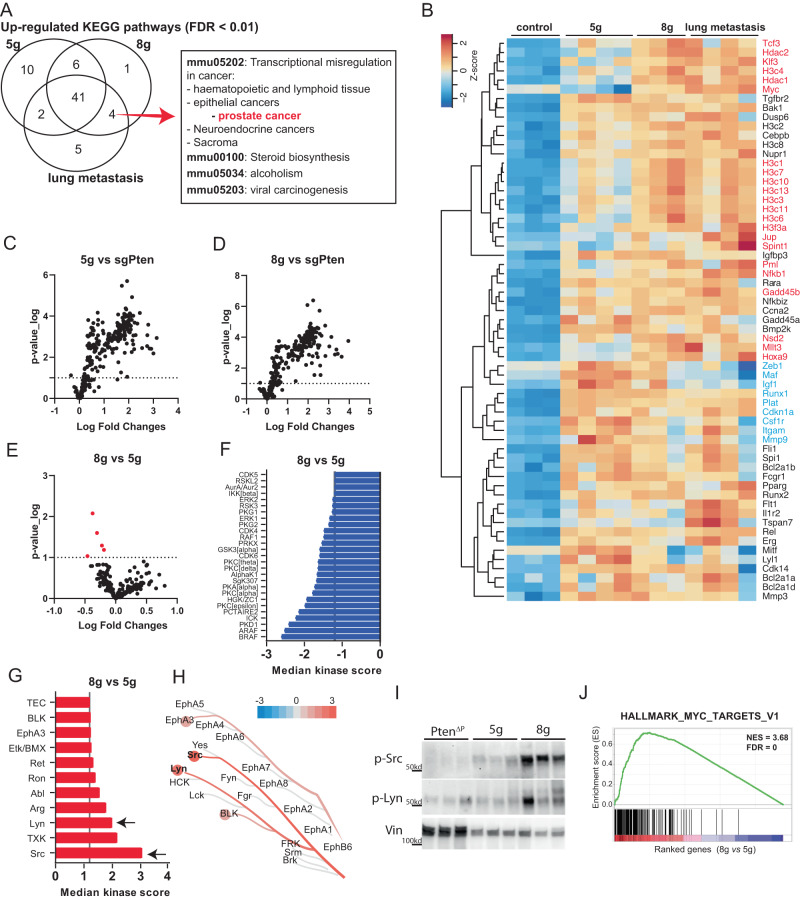
c-Myc pathway was increased with loss of epigenetic factors in Kinome assay and RNAseq. **A** Venn diagram showing the upregulated KEGG pathways in the three different tumor groups comparing to control tissues, based on RNAseq data. **B** Heatmap for the genes involved in transcriptional misregulation in cancer (mmu05202), which were dysregulated and shared by 8 g primary and metastatic samples. Transcriptional-associated genes are marked in red, EMT related genes are marked in blue. **C**–**E** Volcano plot for differential phosphorylated peptides targeted by serine/threonine and protein tyrosine kinases in the primary tumors and Pten control samples (*n* = 4). Graphs indicating the downregulated (**F**) and upregulated kinases (**G**) activity between 5 g and 8 g primary tumor tissues (*n* = 4; cutoff = 1.2). Black arrows mark the kinases of interest, Lyn and Src. **H** They were further visualized in a Coral tree. **I** Western blot for p-Src and p-Lyn on protein lysate from sgPten, 5 g and 8 g samples at 8 weeks after AAV delivery. Vinculin (Vin) was used as loading control (*n* = 3). **J** Gene set enrichment analysis for Myc hallmarks between 5 g and 8 g primary tumors at 6 weeks post AAV delivery (*n* = 4). Source data are provided as a Source Data file.

To gain further insight into molecular mechanisms that drive the metastasis incidence, we conducted KEGG pathway analysis by comparing the three tumor groups to control samples, respectively. We found that four significantly enriched dysregulated pathways were shared by only 8 g derived primary tumors and lung metastases (mmu00100, mmu05034, mmu05202, mmu05203) (Fig. 6A). Among them, mmu05202, transcriptional misregulation in cancer, attracted the most attention. It has been implicated in playing crucial roles in epithelial cancers, including prostate cancer based on KEGG annotation. Within this pathway, a panel of histone H3 genes was highly expressed in 8 g primary tumor and lung metastases, which may be directly stimulated by loss of Kmt2c and Kmt2d^35–37^ (Fig. 6B). In addition, c-Myc, a well-known proto-oncogene that is also a transcription factor, even showing opposite directional expression changes in 8 g tumor and lung metastasis compared with 5 g tumor.

To assess changes in cellular signaling pathways in addition to RNA expression profiles, we performed a protein kinase activities screen for sgPten, 5 g, and 8 g prostate tumors. Two peptide chip arrays were used to measure the activity of serine/threonine and protein tyrosine kinases (STK and PTK, respectively). Overall, both primary tumors had enhanced activity of serine/threonine and tyrosine kinase, compared to benign tumor with only loss of Pten (Fig. 6C–E). To interpret each kinase activity, multiple peptide targets for each kinase were evaluated for phosphorylation. This revealed a group of kinases that were changed between the two subtypes of primary tumors. Among them, Src and Lyn, two non-receptor tyrosine kinases, were more abundant in 8 g primary tumors than in 5 g samples (Fig. 6F–H). Furthermore, western blot analysis confirmed that at protein level, phosphorylated Src and Lyn were enhanced in 8 g tumors compared to both 5 g and sgPten control samples (Fig. 6I). Noting that expression of SRC and LYN were associated with MYC expression, we particularly performed gene set enrichment analysis for the hallmark of MYC targets (HALLMARK_MYC_TARGET_V1) based on RNA expression. Indeed, genes from this hallmark were significantly activated in 8 g compared to 5 g tumors (Fig. 6J). MYC is often amplified in PCa, but we did not find evidence of Myc amplification by WGS (Fig. S6C), indicating that increased Myc activation is transcriptionally regulated. In view of these findings, we proposed that activation of Myc signaling pathway could be enhanced by mutation of the epigenetic factors, likely the Kmt2 genes.

### Loss of Kmt2c effectively drives metastatic formation in the lung

**Fig. 7:**
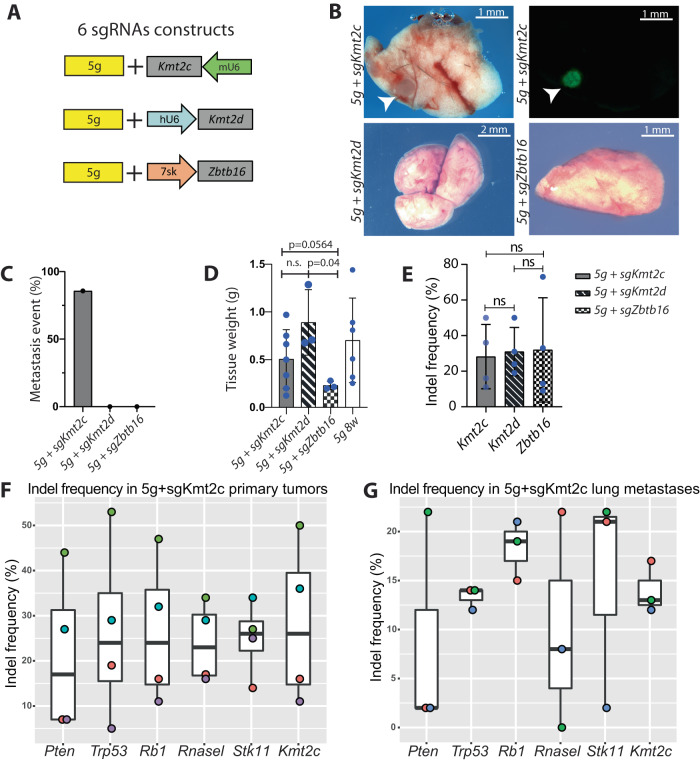
Depletion of Kmt2c drove metastasis formation in the lung. **A** Schematic of three different AAV constructs for cloning the sgRNAs for either Ztbt16, Kmt2d, or Kmt2c, together with the 5 g construct for AAV production. **B** Mice were injected with the AAV particles to the prostatic lobes, and after 8 weeks was, bright field and GFP images taken of the lungs. Representative pictures are shown; white arrowheads mark the metastasis (*n* > 5). **C** Incidents of lung metastasis in three groups of mice (*n* = 7, 3, 3). **D** Weight of the primary tumor at 8 weeks after AAV delivery from three groups of mice (*n* = 7, 3, 3, 6). **E** Indel analysis of Kmt2c, Kmt2d, and Zbtb16 in primary tumors induced by respective AAV particles targeting 5 g with sgKmt2c, sgKmt2d, or sgZbtb16 (*n* = 4). Indel analysis in primary (**F**) and metastasis (**G**) samples at 8 weeks after injection with AAV particles containing 5 g and sgKmt2c (*n* = 3, Box plots present minimum-maximum and percentile data at 25%, 50%, 75%). Data are presented as mean values ±SD as appropriate.

The WGS of the metastatic samples revealed that *Kmt2c* was mutated in all the samples, where intact *Kmt2d* or *Zbtb16* could be found in some metastatic samples, indicating that loss of *Kmt2c* might be crucial for metastatic formation. To genetically assess the implication of the three different epigenetic factors, each CRISPR guide was cloned into the construct containing guides for the five tumor suppressor genes (Fig. 7A). The three different AAV particles were delivered to the murine prostate, and tumors were assessed 8 weeks after initiation. Lung metastases were observed in mice with loss of *Kmt2c* but not in mice where *Kmt2d* or *Zbtb16* were mutated in combination with the five tumor suppressor genes (Fig. 7C). The primary tumor recaptured the weight of the tumor with only the loss of five tumor suppressor genes except the loss of *Zbtb16*, which generated significantly smaller tumors (Fig. 7D). The primary and the metastatic tumors were sequenced for mutations at the target genes. Indel analysis confirmed that all targets were mutated in the primary tumor (Fig. 7E, F). In the secondary tumor *Kmt2c* was mutated together with the other targets, except one sample where *RnaseL* was intact (Fig. 7G). Overall, this analysis showed that loss of *Kmt2c* facilitates lung metastasis formation, whereas loss of Kmt2d or Zbtb16 is dispensable.

### Gene signature differentiated human primary and metastatic prostate cancer

**Fig. 8:**
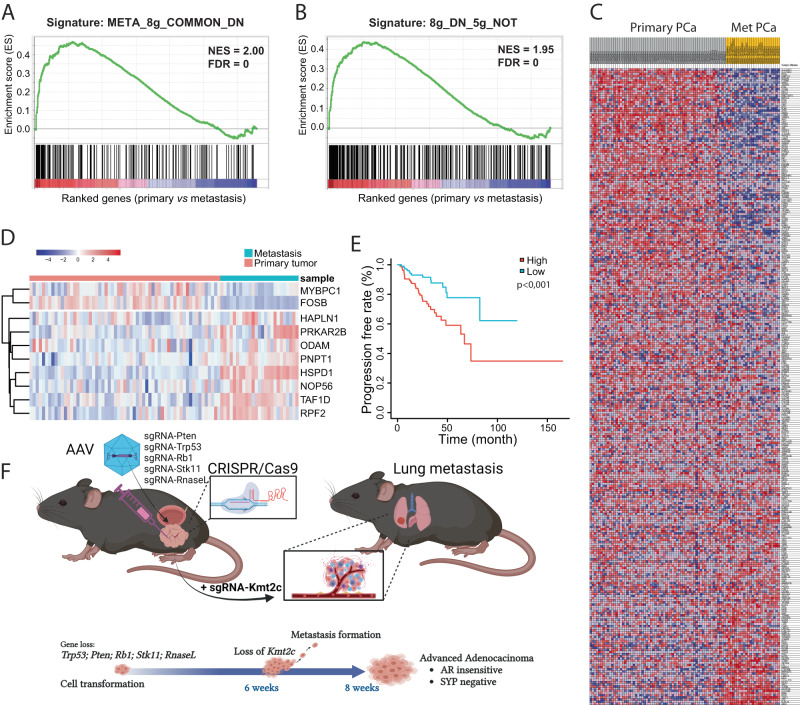
Differential expressed genes can predict clinical outcome of PCa progression. **A** The commonly downregulated 240 genes of 8g-derived primary tumor and metastases. **B**, **C** The downregulated genes (*n* = 394) of 8g-derived primary tumor but not present in 5 g tumors differentiate human PCa into primary and secondary tumors (GSE35988, *n* = 54, 35). **D** A 10-gene set from DEG between 5 g and 8 g tumors distinguishes human primary tumors from metastatic tumors (GSE6919, *n* = 61, 25). **E** A 20-gene set from DEG between 5 g and 8 g was used to predict progression-free survival for TCGA dataset (*n* = 494). **F** Illustration of tumor initiation by five tumor suppressor genes and progression to metastatic disease by loss of Kmt2c at 6 weeks after tumor initiation. Mice reach humane endpoint at 8 weeks after initiation.

To evaluate the clinical relevance of our findings based on mouse tumors, we collected RNA expression data from a human prostate cancer cohort, which included 54 primary and 35 metastatic tumors^7^. We inspect the expression of DEGs, identified in each group of mouse tumors, in human tumors. We found that the shared downregulated genes in 8 g primary and metastatic tumors also tended to be downregulated in human secondary tumors, compared to primary tumors. This also holds true for genes that are downregulated in 8 g tumor but not in 5 g tumor (Fig. 8A–C, S15). We further investigated the expression of 24 DEGs between 8 g tumor and 5 g tumor in the human dataset. Among them, 10 genes were detected in the human data, and their expression profiles were able to distinguish secondary from primary tumors (Fig. 8D). As 5 g and 8 g tumors had different metastasis potential, we finally performed the progression-free survival analysis using the signature score for 20 of these genes based on their expression in TCGA_PRAD data. Indeed, we observed a significant difference between tumors with high and low scores, which was associated with the mutation statues of the 8 target genes particularly KMT2C (Fig S16A). It showed that patients with high scores were correlated with worse progression-free survival (Fig. 8E). Similar trend was observed for overall survival in TCGA_PRAD dataset and another PCa cohort (Fig S16B, C)^38^. Together, our results showed that the dysregulated genes identified in the mouse tumor are consistent with those from human tumors, and more importantly, our study provides several gene signatures that can be used for predicting the prognosis of human prostate cancer patients.

## Discussion

In this study, we applied AAV delivery and CRISPR-Cas9 to mutate and study multiple genes simultaneously in the mouse prostate and their roles during cancer formation. We revealed that loss of five tumor suppressor genes, including the three classical genes *Trp53, Pten,* and *Rb1*, together with two less-explored candidates, *Stk11* and *RnaseL* induced a poorly differentiated tumor over a short time, with humane endpoint eight weeks after virus delivery. We identified a few biopsies with intact *Stk11* and *RnaseL*. However, we assume that these present intratumoral heterogeneity, as a previous study has shown that mice with the loss of only *Trp53*, *Pten*, and *Rb1* reached the humane endpoint after ~20 weeks^14^. Additional mutations in *Kmt2c* facilitated metastasis formation (Fig. 8F), revealing further positive genetic interaction, as loss of Kmt2c in combination with mutation of Pten alone had a long latency (Fig. S4)^39^. The development of human prostate cancer is slow, but our study shows that mutations in multiple key genes interact to develop a very aggressive tumor with rapid expansion and secondary tumor formation. This agrees with the understanding of human cancer biology, where a minimum of mutations was required for tumorigenesis^40^. Mutations in STK11 and RNASEL are less common in human PCa, but lately, the loss of STK11 has been associated with metastatic disease^20,23^. Mutation in *Stk11* and *RnaseL* enhance PCa growth in our mouse model (Fig. S4), but we could not observe an effect on overall survival in patients when analyzing the TCGA dataset. For this, a larger dataset is required to address the impact of these less common mutations. *ZBTB16* is frequently mutated in PCa, but in this study, we found that the loss of Zbtb16 delayed tumor growth, and mutations in this gene were not present in the metastatic samples. The underlying molecular mechanisms are unknown, but it suggests that Zbtb16’s impact on PCa is context-dependent and should be studied further.

We used CRISPR/Cas9 to generate mutations to multiple target genes. Considering the unspecific effect of CRISPR technology reported in a previous study^41^, we performed whole genome sequencing to address potential off-target mutations, which could have driven tumor formation. We identified only one off-target event generated by the *Trp53* sgRNA. This mutation was located at an intergenic region with unknown function but was found in three out of four samples, showing that specific off-target mutations for specific sgRNA can be common. The WGS analysis also revealed that no more than 33 mutations were identified in exonic regions of the four metastatic samples. Of these, few were present in more than one sample, indicating that additional somatic mutations might not be the common driver of the cancer progression. However, a mutation was found in two samples for *Nupr1l*, which is a negative regulator of Nupr1^42^. Interesting, the RNAseq data showed that Nupr1 expression was upregulated in both primary and metastatic tumors. Nupr1 has recently been shown to repress ferroptosis under stress conditions by mediating Lcn2 expression, which was also increased in tumors. Further work will be needed to investigate the possibility of iron-induced cell death in prostate cancer with a focus on the Nupr1/Lcn2 pathway^43^.

At the transcriptome level, the RNAseq analysis identified many differentially expressed genes and enriched pathways when comparing tumor groups to control tissues. This confirmed that mutagenesis in 5 to 8 essential genes is sufficient to induce global transcriptional dysregulation and the development of an invasive tumor. A decrease in AR signature with no obvious upregulation of NEPC scores suggested these tumors belong to a subtype of aggressive PCa, identified as double-negative prostate cancer (DNPC), which is either AR or neuroendocrine driven with the ability of therapy resistance^44,45^. Besides, both 5 g and 8 g derived tumors were resistant to testosterone ablation by castration. This indicated that the model could serve as pre-clinical model to develop more efficient treatment strategies for castration-resistant PCa.

Metastasis formation in pre-clinical models of PCa is an infrequent event. Advanced prostate tumors with only loss of *Pten* has shown to form metastasis in lymph nodes in a few cases^11^. Additional loss of tumor suppressor genes increases the possibility and loss of Smad4 or Kras gain-of-function mutation generates an aggressive primary tumor with metastasis in different organs, including bones^13,46^. In this study, we observed metastasis formation in the lungs of all mice at 8 weeks after initiation of the tumor. Our genetic analysis shows that loss of Kmt2c is essential for metastasis formation in this setting. Interestingly, we did not observe metastasis formation in other tissues such as lymph nodes, liver, or bone, even though this was reported by other models of PCa^13,46^. Recently, it was shown that PCa with loss of *Kmt2c* in combination with *Pten* formed lung metastasis at 18 months but also in other tissues in a subset of the mice^39^. This confirms that loss of *Kmt2c* is associated with metastasis formation, and our work delineated that Kmt2c regulates the formation of lung metastasis. We identified metastasis in the lungs as early as 6 weeks after tumor initiation, which indicated that metastases seeding occur early in the development of the primary tumor. The mechanism for the organotropism to the lung is unknown, but we speculate that loss of Kmt2c is essential for this phenotype.

KMT2C is a histone methyltransferase and is found mutated in many types of cancers^3,47^. KMT2C is not a classic tumor suppressor as loss of KMT2C is not associated with cell proliferation in PCa and we did not see a phenotypic change of the primary tumor with loss *Kmt2c*. Analysis of RNA sequencing identified minor differences between 5 g and 8 g primary tumors. But we found one very interesting region with 150 kb length at chr5, and all six genes inside were strongly upregulated. This region is conserved between mouse and human (4q13.3), which drove us to mutate KMT2C and D in a human benign prostate cell line, BPH1. mRNA expression analysis of genes in this region revealed that loss of KMT2C but not KMT2D was responsible for the upregulation. Interestingly, the genes included in this region have not been previously associated with cancer. However, we generated metastatic cell clones from 8 g tumors and induced mutations in Odam and Cabs1^48^. Orthotopically grafting the cell clones resulted in reduced tumor size when Odam and Cabs1 were mutated, which was not observed in vitro. Furthermore, the loss of Odam and Cabs1 prevented the formation of metastasis, whereas control cells showed metastasis in various organs. Recently, a super-enhancer was discovered upstream of *Odam*, which requires the presence of the *Csn3*-specific enhancer in mouse mammary and salivary glands, suggesting an interaction between genes in this genomic region^49^. Additionally, one clone generated by us was heterozygous for Odam, but the expression levels of this gene were found to be downregulated. This indicates a strong regulatory interaction among multiple genes in this genomic region. Hence, we discovered important functions of Odam and Cabs1, which have not been reported before. Therefore, further research will be essential to elucidate the potential functional pathways of Odam and Cabs1 in the onset of metastatic disease, along with other candidate genes located in the genomic region.

An expression alteration and population changes between luminal and basal cells were seen between tumor types. Others have reported that changes in basal and luminal cells are associated with tumor initiation and metastasis formation^50–53^. Similarly, dysregulations were also found when assessing the signatures of epithelial and mesenchymal cells. EMT is a hallmark in metastasis formation and here we observed the transition of e-cadherin from membrane-bound to cytoplasmic localization in 8 g samples^54^. However, after settling in the lung tissues, e-cadherin was found in the membrane. In addition, upregulation of stemness signatures indicated the emergence of cancer stem cells or basal progenitors. As 8 g tumors and lung metastasis contain similar mutation profiles, this suggests complicated crosstalk in the microenvironment, which interplays with protein localization and cellular migration^55^. We speculate that these changes could be related to the dissemination of tumor cells and the formation of metastasis.

Metastatic PCa carries a poor prognosis, and the identification of a biomarker to predict the risk of metastasis formation from the primary tumor is crucial. We identified a group of DEGs between 5 g and 8 g primary tumors, where only 8 g tumors demonstrated metastatic potential. This set of genes was utilized to predict progression-free survival and distinguish between primary and metastatic tumors in multiple cohorts of human PCa. These findings underscore the high relevance of our pre-clinical model, which involves mutations in 5–8 genes, for understanding the onset of metastatic disease. Thus, this study has provided insights into metastasis formation in PCa, highlighting the essential role of KMT2C loss in regulating a conserved genomic region, including Odam and Cabs1. Understanding the function of KMT2C and the DEGs between 5 g and 8 g tumors is pivotal for advancing PCa diagnostics and treatment in the future.

## Methods

### Mice models

B6J.129(B6N)-Gt(ROSA)26Sortm1(CAG-cas9*,-EGFP)Fezh/J mice were purchased from Jackson Laboratories (catalog no. 26175) together with Pb-Cre4 mouse line (catalog no. 68167). The two mouse lines were interbred to produce Pb4-Cas9/EGFP offspring, ensuring that Cas9 and EGFP would be only expressed in prostatic epithelial cells. The mice were bred and housed at Aarhus University, and all animal experiments were conducted in accordance with the protocol approved by the Danish Animal Experiments Inspectorate, with a maximum tumor size of 1 cubic cm (license no. 2020-15-0201-00711).

### sgRNA design and cloning strategy

All single guide RNA’s (sgRNA), two for each target gene, were designed based on Tefor (http://crispor.tefor.net/crispor.py) or Chopchop (https://chopchop.cbu.uib.no/) webtools. The annealed protospacers were firstly cloned into lentiCRISPR v2 plasmid (Addgene plasmid #52961). The sgRNA with higher insertion or deletion (indel) formation for each gene was selected for the next cloning. In principle, all plasmids were constructed based on considerations of promoter-of-choice and orientations of expression cassettes, to avoid potential recombination or deletion during the cloning and virus preparation. To this aim, the human U6 (hU6)-sgPten expression cassette of pAAV-gPten-CAG-Cre^16^, which was used for generating *Pten* indels in vivo, was reverted with the use of Gibson Assembly Cloning kit (NEB). Then CAG-Cre cassette was replaced with a short fragment containing EcoRI and XmaJI (NEB), resulting pRev-AAV-gPten. As a negative control, a non-targeting (NT)^56^ protospacer was cloned into lentiCRISPR v2 plasmid, followed by amplifying the sgNT cassette with primers containing restriction sites for MluI and HindIII. The PCR product was digested with these two restriction enzymes, and the digested fragment was then ligated to a vector resulting from pAAV-sgPten-hU6 cut by MluI and HindIII.

Next, sgTrp53, sgRb1, sgRnaseL, and sgStk11, together with sgPten, constituted the 5 g group. To construct the 5 g plasmid, the Golden Gate assembly strategy was utilized. The selected protospacers targeting Trp53, Rb1, RnaseL, and Stk11, were separately and randomly cloned into four plasmids containing four different promoters (Addgene plasmids #53186, #53187, #53188, #53189)^57^. Here, sgTrp53, sgRb1, sgRnasel and sgStk11 are driven by 7SK, H1, hU6 and murine U6 (mU6) promoters. These four promoter-gRNA cassettes were then cloned into a lentiviral plasmid, pLV-hUbC-Cas9-T2A-GFP (Addgene plasmid #53190). A colony PCR using 4g-test primers was performed to screen the right clones. Based on the right plasmid, a 1.5 kb fragment comprised of the four promoter-sgRNA cassettes, was amplified with XmaJI_7sk F and EcoRI_mU6 R primers, digested, and then ligated to AAV-sgPten-hU6 vector.

For targeting the three individual epigenetic factors, the three sgRNA cassettes were added in a serial manner. A pair of primers was used to amplify the corresponding promoter-sgRNA cassette, digested, and then ligated to the previous plasmid backbone. By this, an 8 g construct was lastly made. Furthermore, from the basic 5 g plasmid, three different 6 g constructs were cloned, which contained basic 5 g cassettes and one additional sgRNA targeting one of the epigenetic factors. These were named 5 g+sgKmt2c, 5 g+sgKmt2d, and 5 g+sgZbtb16, separately.

To mutate human KMT2C and KMT2D, the protospacers with higher indel frequency in HEK293T cells were chosen from two tested candidates of each gene. Then these sgRNAs, together with scaffold sequence, were synthesized by Synthego for the use of in vitro electroporation in a benign prostatic cell line, BPH1. Protospacers sequence and the corresponding PCR primers are in Supplementary Data S1.

To engineer the lung metastatic cell line, a vector using Puromycin as a selection marker was created based on pLV-hUbC-Cas9-T2A-GFP (Addgene plasmid #53190). First, the plasmid was cleaved using XbaI and EcoRI to remove the coding sequence for Cas9 and EGFP. The Puromycin expression cassette was amplified from lentiCRISPRv2 by PCR, with the addition of XbaI and EcoRI sites at the 5’ and 3’ ends, respectively. The digested PCR fragment was then ligated into the cleaved backbone, resulting in an updated version called pLV-hUbC-Puro.

Next, the annealed synthesized protospacers targeting Cabs1, Smr3a, Csn3, and Odam were individually inserted into four vectors containing mU6, hU6, H1, and 7sk promoters. These four plasmids, along with pLV-hUbC-Puro, underwent a cutting-and-ligating process to produce the pLV-hUbC-Puro-OCCS construct. Similarly, four non-targeting protospacers were cloned into pLV-hUbC-Puro, resulting in the pLV-hUbC-Puro-NonT vector, which was utilized as a negative control. The sequence of the protospacers and PCR primers for Indel analysis can be found in Supplementary Data S1.

### Cell work and virus production

The prostate cell line, BPH1, was cultured in RPMI1640 medium supplemented with 10% fetal bovine serum (FBS, Gibco) and 1% penicillin–streptomycin (Sigma-Aldrich). MEFs cells were isolated from LSL-Cas9 mouse embryos, as described before^58^. MEF and HEK293T cells were grown in DMEM (Sigma-Aldrich) supplemented with 1% penicillin–streptomycin and 10% FBS. All cells were incubated at 37 °C in humidified atmospheric air with 5% CO_2_ at 37 °C. For transfection of MEF and HEK293T cells, X-tremeGENE-9 reagent was utilized according to the manufacturer protocol. Puromycin (2ug/ml) was applied one day after transfection. With four days’ selection, cells were collected for DNA extraction and further validation of the indel formation. For BPH1, nucleofection of synthesized gRNA oligos (Synthego) was electroporated together with Cas9 Nuclease V3 protein (Alt-R®, IDT), by using 4D-Nuleofector^TM^ and the optimized protocol for PrEC cell line by the manufacture. Five days later, cells in bulk were collected, meanwhile, 3000 cells were seeded to a 10 cm dish, to form single-cell clones.

AAV production method was as described before^58^. Briefly, 14 μg Adeno-helper plasmid, 14 μg serotype 9 capsid plasmid, 14 μg plasmid of interest, and 2.2 μg GFP plasmid (serving as transfection control) would be transfected to HEK293T cells of a 15 cm dish, using the branched MW 25,000 polyethylenimine (PEI) (Sigma-Aldrich, 408727) reagent. After 60 hours, all cells were collected and lysed, followed by virus preparation. The produced virus genome was titrated by qPCR using a standard curve of the corresponding plasmid and 10^4^-to-10^6^-fold dilutions of the AAV sample.

To generate lentivirus, 13 μg of pMDLg/pRRE (Addgene #12251), 3.75 μg of pMD2.G (Addgene #12259), 3 μg of pRSV-REV (Addgene #12253), and 13 μg of either pLV-hUbc-Puro-OCCS or pLV-hUbc-Puro-NonT were transfected into HEK293T cells in a 10 cm dish. After 72 hours, the supernatant was collected, centrifuged, and sterilized using a 0.45 μm syringe filter, then stored at −80 °C for further use.

### Establishment of primary tumor cells

PCa metastases were dissected from lung tissues under a fluorescent microscope. The isolated tissues were minced with scissors in complete DMEM supplemented with FBS, penicillin–streptomycin and Gentamicin. The tissue fragments were mechanically dissociated by vigorous pipetting with a 10 ml serological pipette before being washed twice in PBS. Subsequently, the fragments were digested in Accutase supplemented with DNaseI (Thermo Fisher) and ROCK inhibitor (Y27632; STEMCELL Tech) at 37 °C for 30 minutes. The cells were then resuspended in DMEM and filtered through a 100 μm mesh followed by a 70 μm mesh to select for single cells and small cell clusters. The cells were resuspended in Neurobasal medium supplemented with B27, Glutamax (NBM; Life Technologies), hEGF (20 ng/ml), FGF2 (20 ng/ml), Heparin (100 ng/ml; Peprotech), and dihydrotestosterone (DHT, 100 nM; Methods), and cultured in a low-attachment flask.

Primary cells transduced with LV underwent puromycin selection (2 μg/ml) two days after infection and were selected for 10 days. The single cells were sorted by FACSAria III Cell Sorter (BD) into 96-well plates with low-attachment, and clones were generated.

### Orthotopically delivery of virus and cell lines to the mouse prostate and surgical castration

Each virus with 10^9^ virus genome (vg) was orthotopically injected into each anterior lobe of the mouse prostate allowing the viral particles to disseminate to the other prostatic lobes, as described previously^17^. As a negative control, the non-targeting virus or PBS was injected. Implantation of the metastatic cell clones was performed using 200,000 single cells resuspended in twice-diluted Matrigel (Sigma-Aldrich), in a total volume of 25 µl. The cells were injected into one anterior prostatic lobe. Castration was performed on a group of mice 5 weeks after virus delivery. For castration, the mice were anesthetized, and an insertion was done in the abdomen, then the testis were lifted out, blood supply was ligated, and the testis were removed before the abdomen was closed.

### PET and MRI scanning

Positron emission tomography (PET) and magnetic resonance imaging (MRI) were used to evaluate disease burden in animals. Anesthesia was induced using isoflurane, administered via a respiration mask throughout the scanning procedure. The animals were given an intraperitoneal injection of either [18 F]-flurodeoxyglucose (FDG) or [18 F]-sodium fluoride (NaF) at a dose of ~10 MBq/animal. The PET scan was conducted 60 minutes post-injection, with a duration of 10 minutes, followed by a T1 weighted MRI scan lasting 30 minutes. PET images were reconstructed using a three-dimensional ordered subset expectation algorithm (Tera-Tomo 3D) with four iterations and six subsets, at a voxel size of 0.6 × 0.6 × 0.6 mm3. Data was corrected for dead-time, decay, and randoms, and presented as Standardized Uptake Value (SUV) without corrections for attenuation and scatter.

### DNA and RNA isolation

Tumor or prostate tissues were collected at 4 weeks, 6 weeks, and 8 weeks post virus injection. For the castrated mice, tumors were obtained three weeks after surgery castration. Human cell lines from two different clones of KMT2C and/or KMT2D KO were collected. DNA and RNA were isolated from the samples by Qiagen AllPrep DNA/RNA kit according to manufactures protocol.

### Indel analysis based on Sanger sequencing and WB

Primers were designed to cover the CRISPR-Cas9 target regions. PCR was performed on DNA samples from cell lines or tumor tissues, and the product was purified with a gel-isolation kit (Thermo Fisher), followed by Sanger sequencing. Then indel formations were analyzed by ICE webtool (Synthego). The potential off-target sites were predicted by Chopchop and Tefor webtools, as listed in Supplementary Data S2.

To validate the loss of protein by newly designed sgRNAs, Western blot analysis was performed for targets where antibodies could be obtained. These analyses were conducted on cell lines generated from the tumor samples to avoid interference from non-tumorigenic cells.

### Quantitative real-time PCR

Primers were designed by Primer 3.0 tool and were spending and intron. Quantitative Real-Time PCR (qRT-PCR) was performed on 20 ng total RNA samples by Brilliant III Ultra-145 Fast SYBR® Green QPCR Master Mix (Agilent Technologies). All data were analyzed with the ΔΔCT method and normalized to housekeeping genes. For mouse tissue, the geometric mean of Ct values for Rpl4 and Rpl32 was used for normalization. For human cell lines, all gene expressions were normalized to RPL4. See Table S1 for primers.

### Histochemical and immunohistochemistry analysis

Tissue samples were fixed in 4% paraformaldehyde (Santa Cruz Biotechnology), followed by dehydrating and then embedding in paraffin. Tissue sections of four μm were cut and underwent deparaffinization. Antigen was retrieved by heating at 100 °C in a citrate buffer at pH 6. Sections were blocked in 2.5% BSA (Sigma-Aldrich) in PBST prior to incubation with the following primary antibodies: pAkt (CST, 4060), Ki67 (abcam, 16667), E-cadherin (BD Biosciences, 610181), AR (Millipore, 06-680), SYP (Santa Cruz, sc177501), p63 (CST, 39692), Ck5 (BioLegend, 905501) or Ck8 (BioLegend, 904801). Next, appropriate horseradish-peroxidase-conjugated (Vector Laboratories) or florescent secondary antibodies were used (Invitrogen). Counterstaining was performed with hematoxylin or DAPI.

### Western blot and kinome analysis

To prepare protein lysate, M-PER mammalian extraction buffer (Pierce, Thermo Scientific) supplemented with 1× protease inhibitor cocktail was added to cell pellets and pieces of mice prostate or tumor tissues. For mice tissues, additional steps of tissue homogenization and sonication were applied. Concentrations of the isolated products were determined by Pierce BCA protein assay kit (Thermo Scientific), and samples were prepared with Laemmli loading buffer for WB. Lysates were separated on SDS-PAGE gel and transferred to a PDF membrane. The following antibodies were applied: Trp53 (CST, 2524), Pten (CST, 9188), pRb1 (sc102), Stk11(sc32245), Odam (16509-1-AP), p-Src (CST, 2101), p-Lyn (CST, 2731), Vinculin (Sigma, v9131), followed by incubation with its relative secondary antibodies. The bands were visualized with ECL reagent (GE Healthcare).

For Kinome analysis, the PamGene chips were used according to the manufactory protocol. Five individual protein samples from each group (sgPten, 5 g, and 8 g) were included. Assays were performed on a PamStation12 (PamGene, Netherlands). Briefly, the tyrosine kinase assay (PTK) was processed in a single-step reaction. Tissue extracts, ATP, and fluorescein isothiocyanate (FITC)-labeled pY20 antibody were incubated on the chip, and the phosphorylation of tyrosine peptides was detected by fluorescence signal in real-time. For the serine-threonine kinase (STK) array, a two-step reaction was designed. First, the sample mix of tissue extracts, ATP, and the primary antibody were incubated for 110 min. Next, the sample mix was washed, followed by adding a detection mix containing the secondary FITC-labeled antibody. Development of the FITC fluorescence signal was then performed. Signal intensities were analyzed in the BioNavigator software (PamGen) and expressed as log2 fold changes versus sgPten control. The differential kinases among the groups were analyzed by the Upstream Kinase Analysis (UKA) tool. The kinome tree was made by CORAL tool (http://phanstiel-lab.med.unc.edu/CORAL/).

### Whole genome sequencing

Four DNA samples of lung metastasis, three of which matched with RNAseq experiment, were included for whole genome sequencing (WGS). Short insert fragment library was prepared by BGI (Denmark), and then PE sequencing with 150nt read length was performed on DNBseq platform, which produced 96 Gb raw data per sample. The raw data was filtered after removing adaptor sequences. The filtered fastq files were mapped to the mouse genome mm39 using BWA^59^, and the alignment was sorted with samtools^60^. Duplicates were removed with MarkDuplicates, the individual variant calls were found using Mutect2 and filtered by FilterMutectCalls all from the Genome Alignment Toolkit (GATK)^61^. The mean base quality and mean mapping quality are shown in Supplementary Data S3 and S4. All were annotated to genes by ANNOVAR^62^ using the mm39 reference genome. As no normal sample was sequenced to remove germline mutations, the variants found in all 4 samples were flagged, as potential germline variants. Myc amplification was assessed by comparing the average read for chr15: 61857190-61862210 (Myc gene) to the average read for the entire genome.

### RNAseq analysis

Transcriptomic analysis was performed on 5 g and 8 g derived primary tumors (*n* = 4) and macro dissected lung metastases from 8 g group (*n* = 4), using prostatic tissues from PBS-injected Cas9:PB4Cre positive mice as control (*n* = 3). Libraries were prepared using an Illumina KAPA mRNA Hyperprep kit, followed by Illumina high-throughput sequencing on the NovaSeq 6000 platform. An average of 45 million paired-end (PE) reads with 150nt read length (150 × 2) were obtained for each sample. Adaptors were trimmed by Cutadapt^63^(v3.2), and clean reads were aligned to the mouse reference genome (mm10) with the incorporated transcriptome annotation from Gencode (vM25) using STAR (v.2.79)^64^. FeatureCounts (v2.0.1)^65^ was applied to count the aligned fragments for each gene from the Gencode (vM25) annotation. Raw gene counts were normalized across the 15 samples, and differently expressed genes (BH-adjusted *p* value < 0.05, log2 fold change >1) between each two groups of samples were identified using DESeq2 (v.1.3.2)^66^ in R environment (v4.1.0). The list of differentially expressed genes is provided in Supplementary Data S5.

To check the expression pattern of multiple well-known signature genes in each group (control, 5 g, 8 g, and lung met samples), we first extracted 21/22 genes for mesenchymal/epithelial, 22/31 genes for basal/luminal, 17 genes related to AR, 11 genes for NEPC^31,33,67^. Besides, we used four different gene signatures to assess the stemness feature of the tumor tissues^68–71^. Next, we calculated the average of *z* scores for genes in each signature and visualized them with library ‘ggpubr’(v.0.4.0) in R environment (v4.1.0).

Functional enrichment analysis of differentially expressed genes in each comparison was conducted by using the WEB-based Gene Set Analysis Toolkit (WebGestalt 2019, http://webgestalt.org) for Gene Ontology (GO) terms, Kyoto Encyclopedia of Genes and Genomes (KEGG) pathways and chromosome locations. Enriched molecular signatures and cancer hallmarks were identified using GSEA (v3.0) with annotation from the Molecular Signatures Database (MSigDB, v6.1). To utilize annotations from MSigDB, mouse genes were mapped to their corresponding human orthologs using BioMart from Ensembl (https://www.ensembl.org). The significantly enriched (FDR < 0.05) pathways, GO terms, cancer hallmarks, and signatures were provided in Supplementary Data S6.

The expression of genes that mapped to human orthologs was compared to two public datasets from GEO with session numbers GSE6919^72,73^, GSE16560^38^, and GSE35988^7^. The 24 signature genes identified by comparing 8 g with 5 g were used for overall and progression-free survival analysis in 494 Prostate adenocarcinoma (PRAD) patients from TCGA using the average z-scores of the RNA expression from cBioportal (http://www.cbioportal.org).

### Data mining and visualization

GraphPad Prism software (v.9.4.0) and R-studio (v1.4), R (v.4.1.1) were used for statistical calculations and data visualization (Biorender). N is representative of a unique tumor in a unique animal, and a non-parametric *T* test was used for statistical calculations where nothing else was written.

### Reporting summary

Further information on research design is available in the Nature Portfolio Reporting Summary linked to this article.

### Supplementary information

Supplementary Information

Peer Review File

Reporting Summary

Description of Additional Supplementary Files

Supplementary Data 1-6

### Source data

Source Data

## Data Availability

Source data are provided in this paper, and the RNAseq and WGS data generated in this study have been deposited in the NIH database under accession codes PRJNA1053864 and PRJNA1061795.
