# Supplementary material for: CRISPR/Cas9 model of prostate cancer identifies Kmt2c deficiency as a metastatic driver by Odam/Cabs1 gene cluster expression
Source: Nat Commun. 2024 Mar 7;15:2088. doi: 10.1038/s41467-024-46370-0 (PMC10920892; doi:10.1038/s41467-024-46370-0)
Supplement: Supplementary file 1 — Supplementary Information [file 41467_2024_46370_MOESM1_ESM.pdf]

A

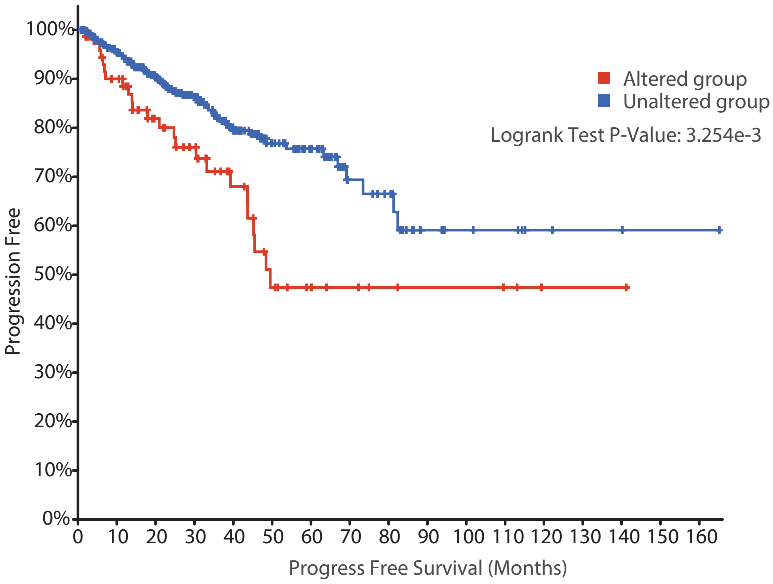
Altered group
Unaltered group
Logrank Test P-Value: 3.254e-3
Progression Free
100%
90%
80%
70%
60%
50%
40%
30%
20%
10%
0%
0 10 20 30 40 50 60 70 80 90 100 110 120 130 140 150 160
Progress Free Survival (Months)

B

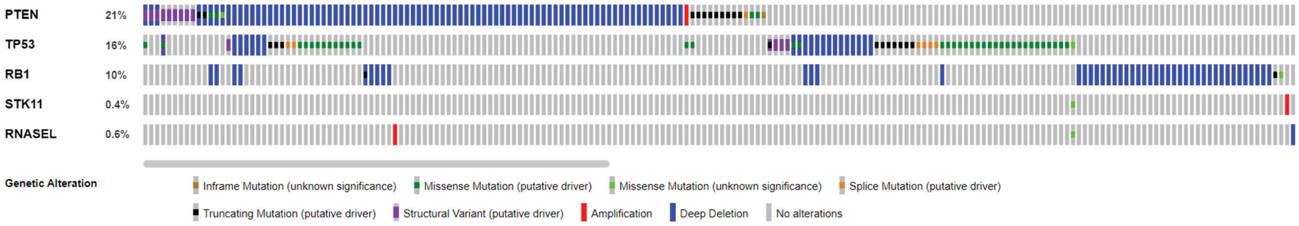
PTEN 21%
TP53 16%
RB1 10%
STK11 0.4%
RNASEL 0.6%
Genetic Alteration
Inframe Mutation (unknown significance)
Missense Mutation (putative driver)
Missense Mutation (unknown significance)
Splice Mutation (putative driver)
Truncating Mutation (putative driver)
Structural Variant (putative driver)
Amplification
Deep Deletion
No alterations

C

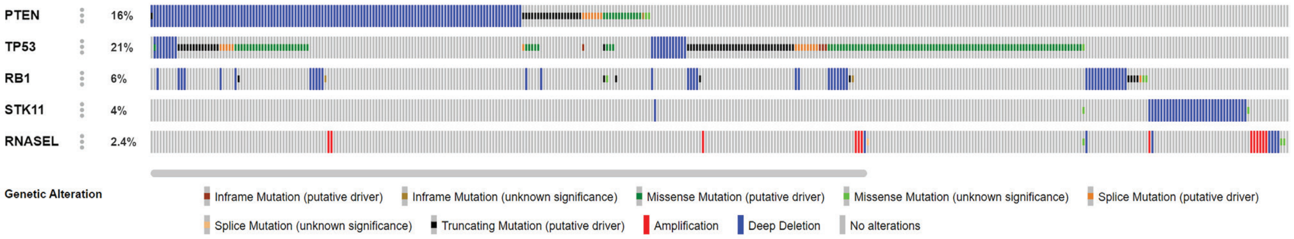
PTEN 16%
TP53 21%
RB1 6%
STK11 4%
RNASEL 2.4%
Genetic Alteration
Inframe Mutation (putative driver)
Inframe Mutation (unknown significance)
Missense Mutation (putative driver)
Missense Mutation (unknown significance)
Splice Mutation (putative driver)
Splice Mutation (unknown significance)
Truncating Mutation (putative driver)
Amplification
Deep Deletion
No alterations

Figure S1: Mutations in TP53, PTEN, RB1, STK11 and RNASEL is associated with worse prognosis

(A) Analysis of progression free survival determined by groups with and without alterations of five tumor repressor genes, TP53, PTEN, RB1, STK11 and RNASEL, based on TCGA_PRAD dataset (n=494). Oncoprint plot showing distribution of mutations in TP53, PTEN, RB1, STK11 and RNASEL in the (B) TCGA_PRAD dataset (n=494), and (C) the MSK/DFCI cohort (n=1013).

A

Gene:	RnaseL	Rb1	Kmt2d	Kmt2c	Zbtb16
indel:	18.90%	55.30%	72.90%	83.30%	91.40%

B

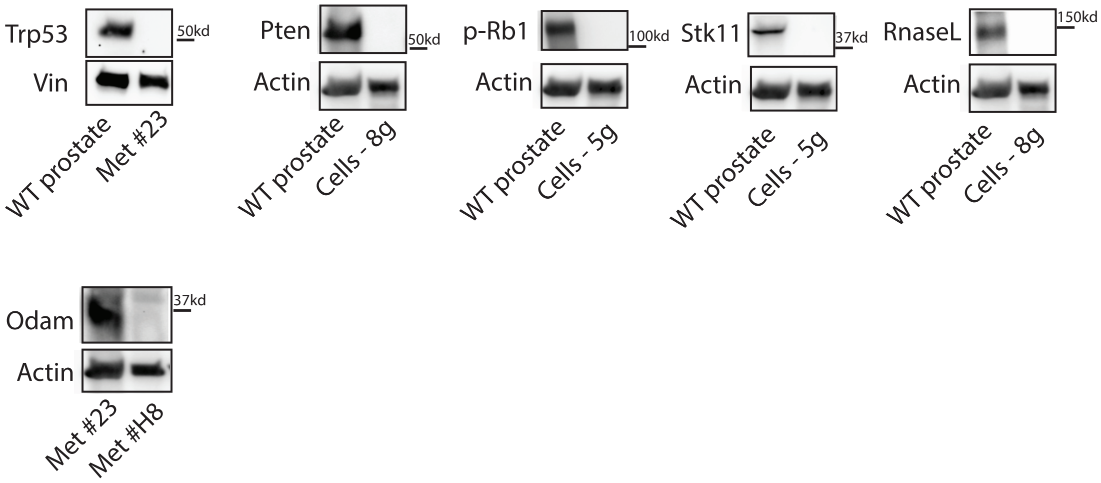

Figure S2: Loss of protein expression by CRISPR induces mutations

A: Induction of indels was validated for novel sgRNAs in MEF cells. B) Cell lines generated from primary or secondary tumors were used to analyze protein expression for genes mutated by CRISPR. Protein from wild-type prostate, or a cell line (Met #23), was used as a control. Vinculin (Vin) or beta-Actin were used as loading controls. Source data are provided as a Source Data file.

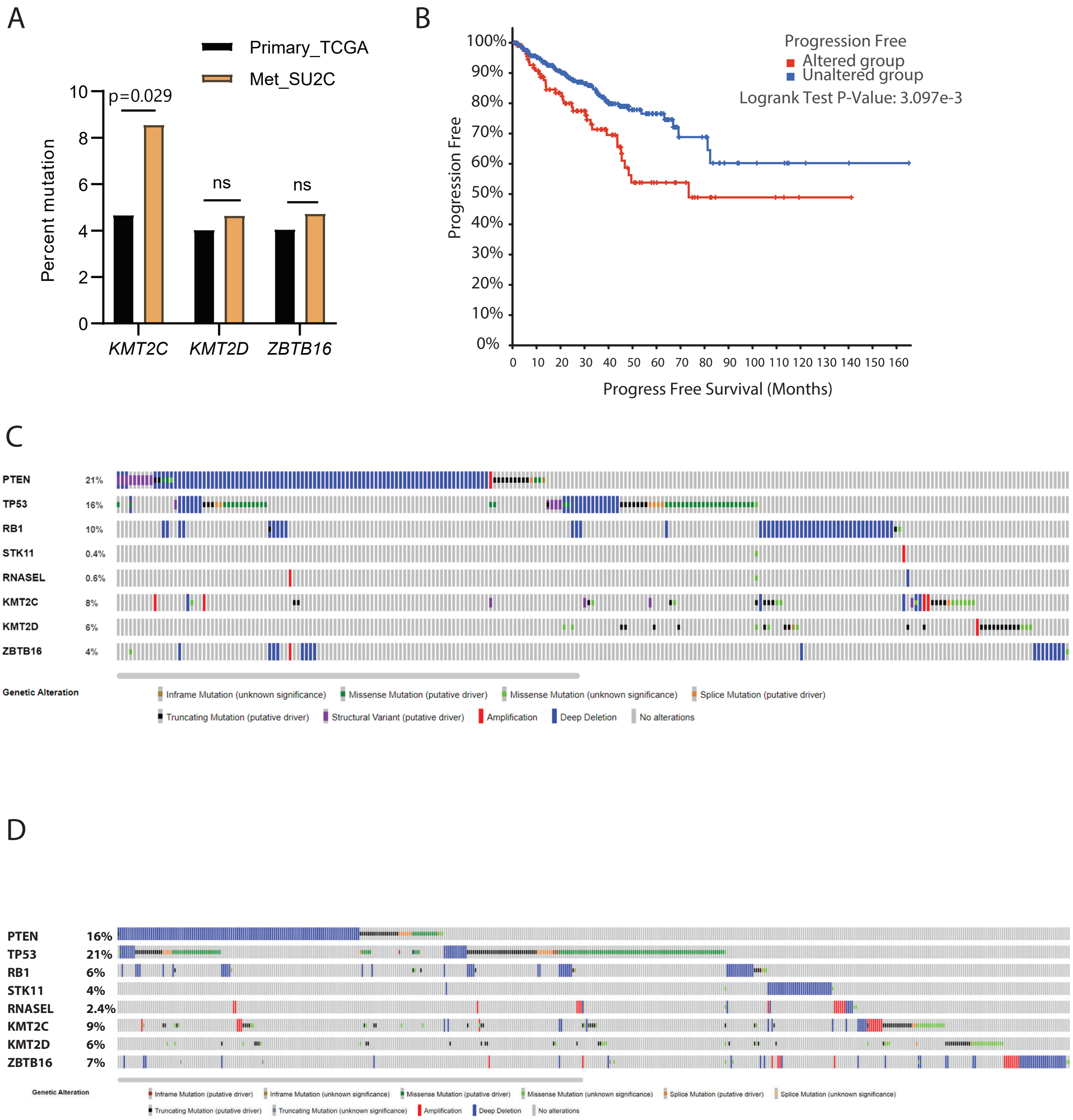
A
Primary_TCGA
Met_SU2C
Percent mutation
p=0.029
ns
ns
KMT2C
KMT2D
ZBTB16
B
Progression Free
Altered group
Unaltered group
Logrank Test P-Value: 3.097e-3
Progression Free
Progress Free Survival (Months)
C
PTEN 21%
TP53 16%
RB1 10%
STK11 0.4%
RNASEL 0.6%
KMT2C 8%
KMT2D 6%
ZBTB16 4%
Genetic Alteration
Inframe Mutation (unknown significance)
Missense Mutation (putative driver)
Missense Mutation (unknown significance)
Splice Mutation (putative driver)
Truncating Mutation (putative driver)
Structural Variant (putative driver)
Amplification
Deep Deletion
No alterations
D
PTEN 16%
TP53 21%
RB1 6%
STK11 4%
RNASEL 2.4%
KMT2C 9%
KMT2D 6%
ZBTB16 7%
Genetic Alteration
Inframe Mutation (putative driver)
Inframe Mutation (unknown significance)
Missense Mutation (putative driver)
Missense Mutation (unknown significance)
Splice Mutation (putative driver)
Splice Mutation (unknown significance)
Truncating Mutation (putative driver)
Truncating Mutation (unknown significance)
Amplification
Deep Deletion
No alterations

Figure S3: Mutations in KMT2C is increased in metastatic PCa

(A) Percent of mutation counts in KMT2C, KMT2D and ZBTB16 from TCGA-based primary PCa (n=494) and SU2C-derived metastatic PCa datasets (n=429). For KMT2C and D was mutations of unknown significance exclude. Fishers exact test was used. (B) Analysis of progression free survival determined by groups with and without alterations in eight genes: KMT2C, KMT2D, ZBTB16, TP53, PTEN, RB1, STK11 and RNASEL in the TCGA_PRAD dataset (n=494). Oncoprint plot showing distribution of mutations in the eight genes in (C) the TCGA_PRAD dataset (n=494), and (D) the MSK/DFCI cohort (n=1013)

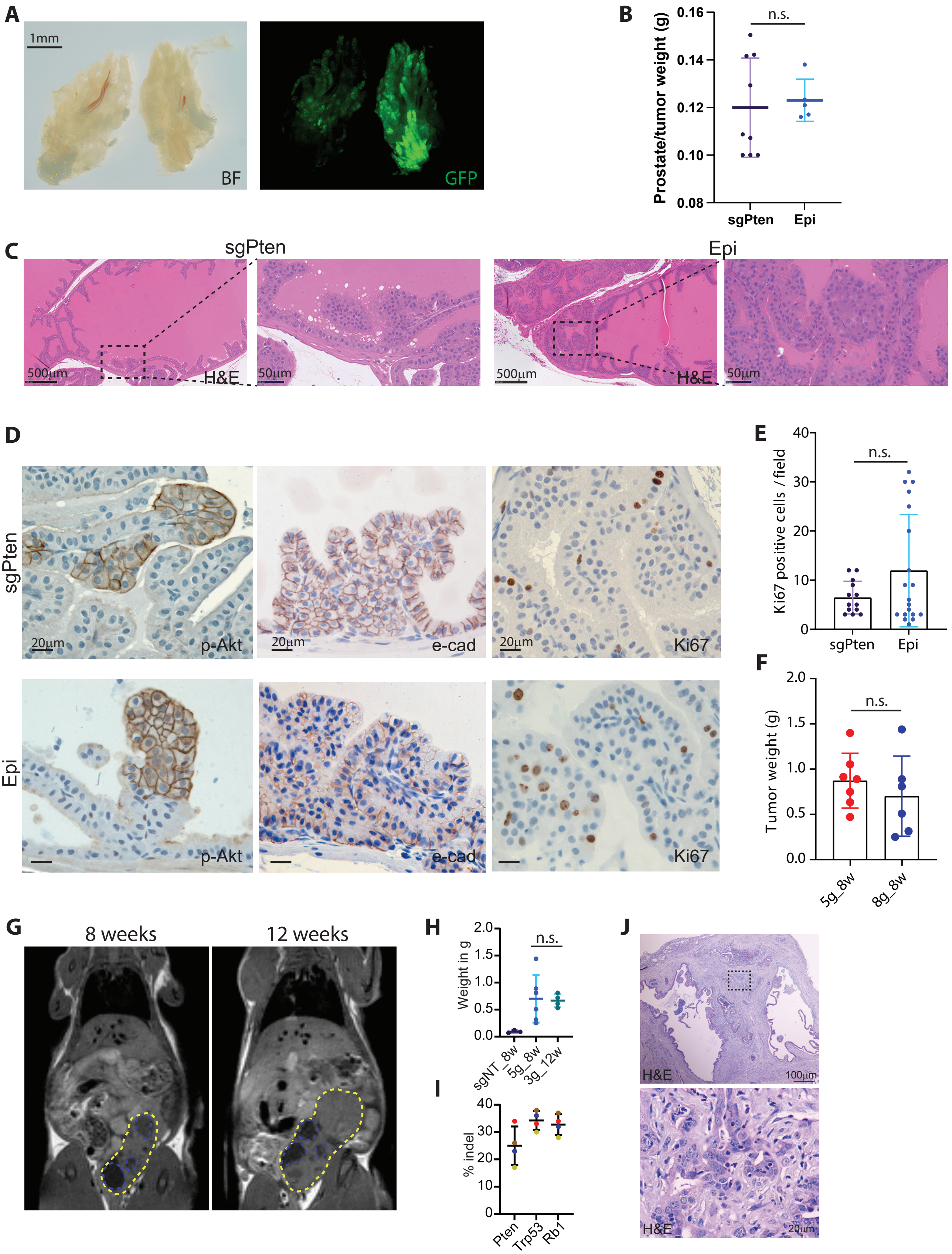
A
1mm
BF
GFP
B
Prostate/tumor weight (g)
0.16
0.14
0.12
0.10
0.08
n.s.
sgPten
Epi
C
sgPten
Epi
500μm
50μm
H&E
D
sgPten
Epi
20μm
p-Akt
e-cad
Ki67
E
Ki67 positive cells / field
40
30
20
10
0
n.s.
sgPten
Epi
F
Tumor weight (g)
2.0
1.5
1.0
0.5
0.0
n.s.
5g_8w
8g_8w
G
8 weeks
12 weeks
H
Weight in g
2.0
1.5
1.0
0.5
0.0
n.s.
sgNT_8w
5g_8w
3g_12w
I
% indel
40
30
20
10
0
Pten
Trp53
Rb1
J
H&E
100μm
20μm

Figure S4: Combinational loss of Pten and three epigenetic factors did not accelerate tumor progression.

(A) Mice were orthotopically injected with AAV particles targeting Pten and three epigenetic factors, Kmt2c, Kmt2d and Zbtb16. Macroscopic pictures were taken with bright field and GFP channel at 8 weeks after injection. (B) The weight of the prostates from mice injected with Epi-AAV was compared to control mice with only Pten mutated (n=9, 4 for each group). (C) H&E staining indicated the benign prostatic tumors in both sgPten and Epi groups. (D) IHC were performed on tissue sections of sgPten and Epi prostates, to detect abundance of p-Akt, E-cad and Ki67 ($n \geq 3$). (E) Comparison of Ki67-positive cells between sgPten and Epi groups (n=13, 19 fields). n.s.: not significant (Mann-Whitney U test). (F) The weight of the prostates was measured at 8 weeks after administration of the AAV particles for 5g and 8g tumors (n=7). G) Mice were injected with AAV containing sgRNAs for Pten, Trp53, and Rb1. Cancer development was monitored with MRI scans at 8 weeks and 12 weeks after. The dashed yellow line marks the prostate, and the blue dashed line marks cystic areas. Representative pictures are shown (n=4). H) The weight of the prostates was measured at termination and compared to 5g samples and non-target control samples (NT) (n=3,6,4). I) Indel formation in tumors at 12 weeks after the delivery of AAV particles was assessed (n=4). J) H&E staining of prostatic tissues at 12 weeks after cancer initiation. A representative picture is shown (n=4).

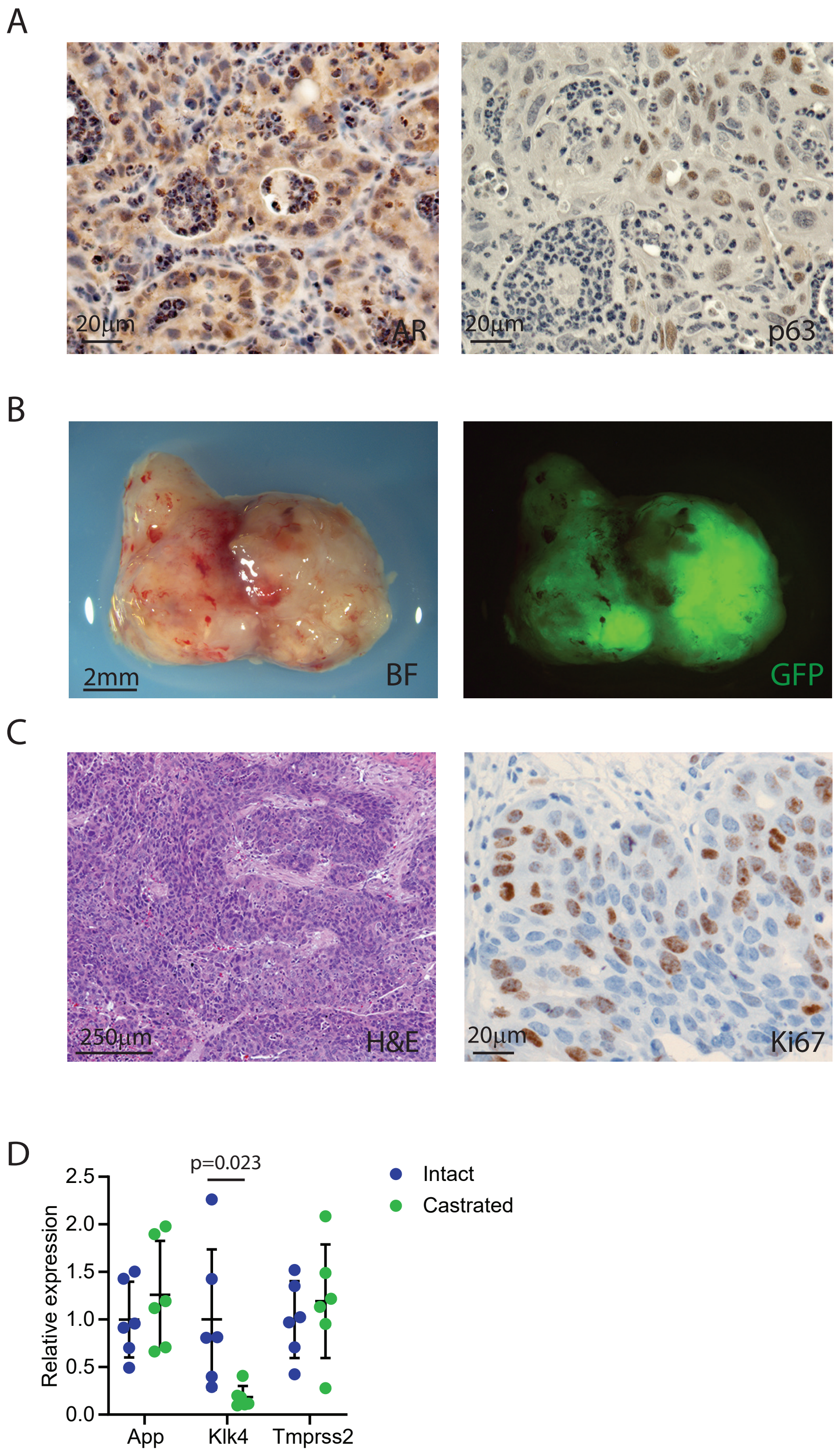
A
20µm
AR
20µm
p63
B
2mm
BF
GFP
C
250µm
H&E
20µm
Ki67
D
Relative expression
2.5
2.0
1.5
1.0
0.5
0.0
p=0.023
App
Klk4
Tmprss2
Intact
Castrated

Figure S5: Epithelial derived metastases and castration resistant PCa.

(A) Mice were injected with 8g-derived AAV particles and lung metastases were stained for AR or p63 (n=5). (B) A group of mice were castrated at 5 weeks after injections and sacrificed at 8 weeks. Macroscopic pictures were taken with bright field and GFP channel at 8 weeks and representative pictures are shown (n=7). (C) H&E staining and IHC detecting Ki67 were performed on tissue sections of 8g-castrated mice (n=7). (D) Expression analysis of 3 AR-regulated genes was performed on total RNA from intact or castrated mice at 8 weeks after tumor initiation (n=6). Source data are provided as a Source Data file.

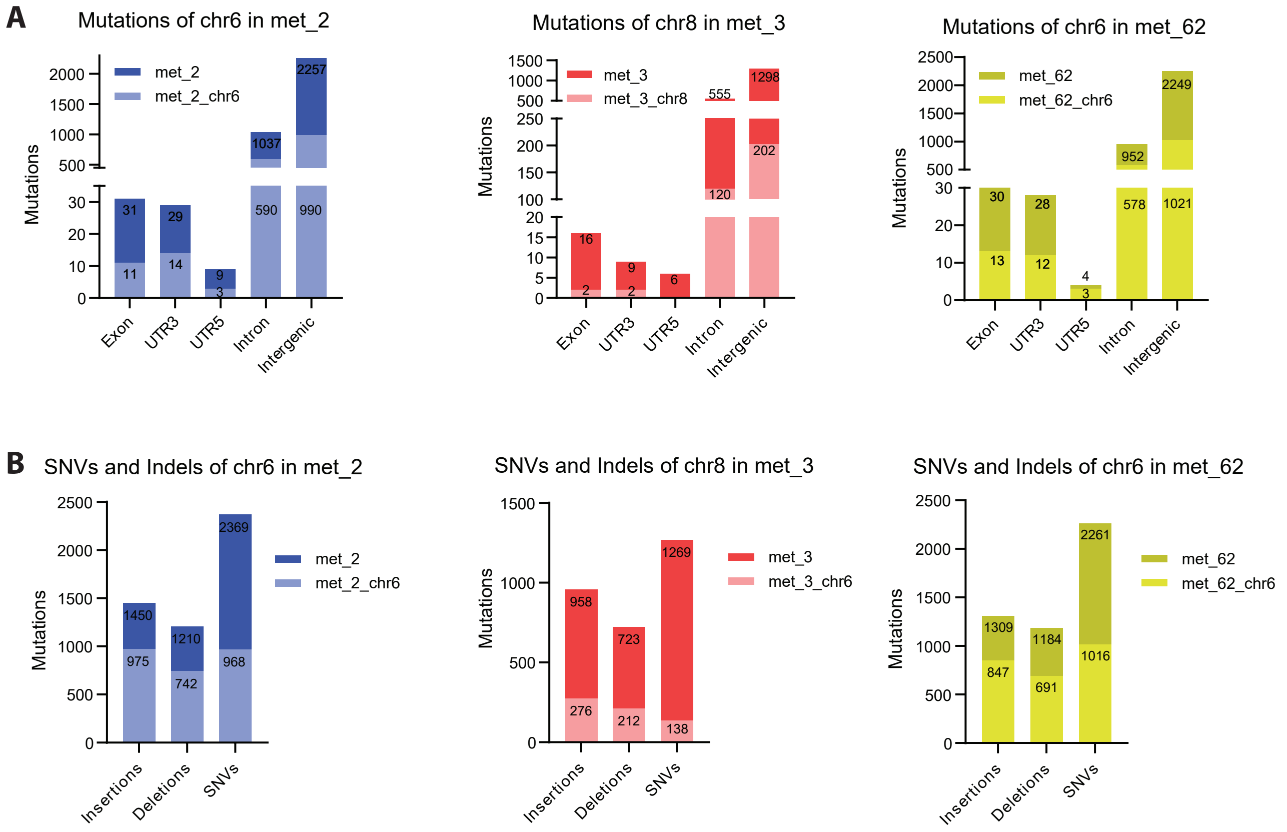

C

**Genome depth**				
Sample	met_2	met_3	met_5	met_62
Average genome	38,7	36,6	37,0	35,6
Average MYC	36,9	36,9	33,9	33,9

Figure S6: Ectopic and high mutation burden was found at chromosome 6 and 8.

(A) Mutation counts at chr6 for sample lung_2 and Lung_62, and at chr8 for sample lung_3. Genomic features including exon, UTR3, UTR5, intron or intergenic regions are listed. (B) Plots indicating the SNVs, Indels and overall counts at chr6 and chr8 for the representative samples. (C) The average reads for the locus containing c-Myc were compared to the overall read coverage (n=4).

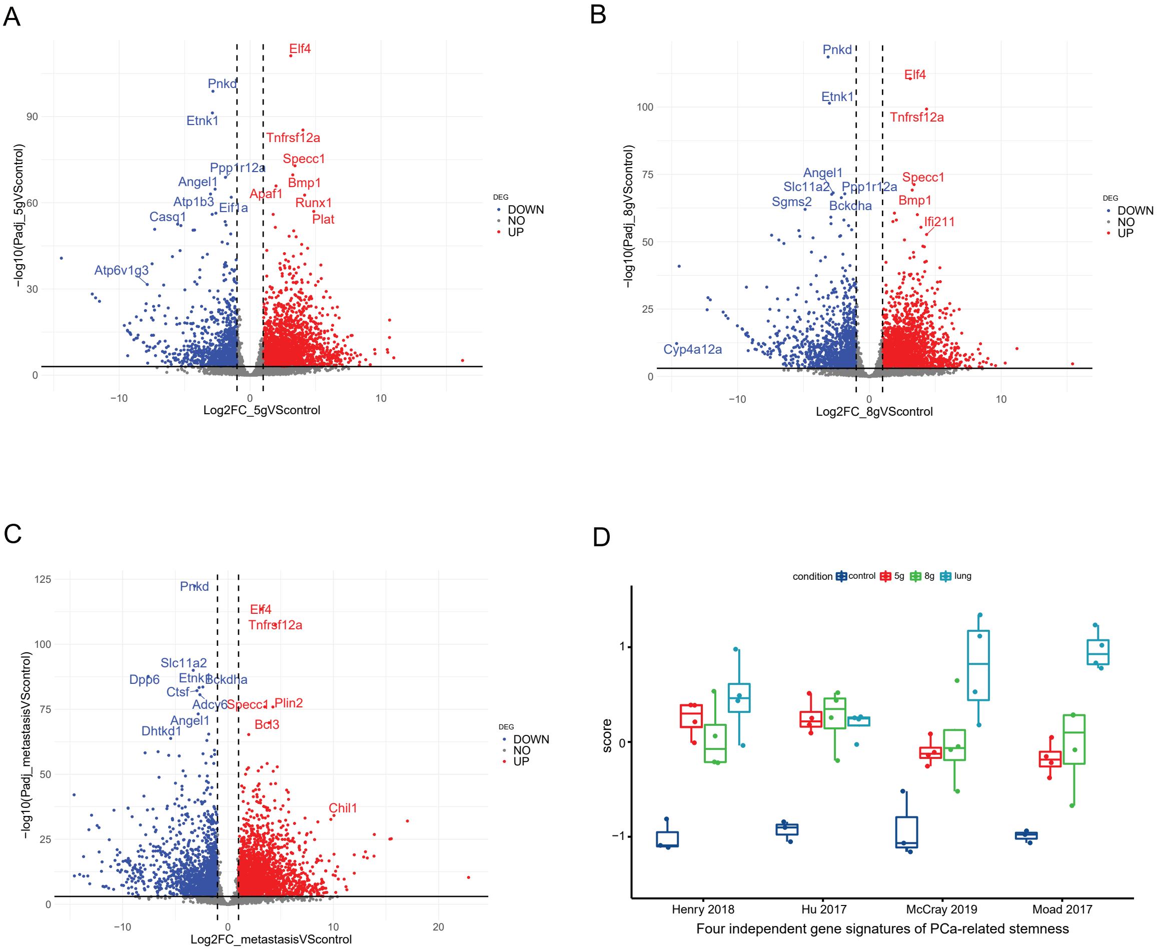
A
Elf4
Pnkd
Etnk1
Tnfrsf12a
Specc1
Ppp1r12a
Angel1
Bmp1
Apaf1
Atp1b3
Runx1
Eif1a
Casq1
Plat
Atp6v1g3
−log10(Padj_5gVScontrol)
Log2FC_5gVScontrol
DEG
DOWN
NO
UP
B
Pnkd
Elf4
Etnk1
Tnfrsf12a
Angel1
Specc1
Slc11a2
Ppp1r12a
Sgms2
Bckdha
Bmp1
Ifi211
Cyp4a12a
−log10(Padj_8gVScontrol)
Log2FC_8gVScontrol
DEG
DOWN
NO
UP
C
Pnkd
Elf4
Tnfrsf12a
Slc11a2
Dpp6
Etnk1
Bckdha
Ctsf
Adcy6
Specc1
Plin2
Angel1
Bcl3
Dhtkd1
Chil1
−log10(Padj_metastasisVScontrol)
Log2FC_metastasisVScontrol
DEG
DOWN
NO
UP
D
condition
control
5g
8g
lung
score
Henry 2018
Hu 2017
McCray 2019
Moad 2017
Four independent gene signatures of PCa-related stemness

Figure S7: Transcriptome analysis revealed many DEGs in all tumor groups, including PCa-stemness related genes.

(A-C) Volcano plots over DEGs in all tumor groups comparing to control tissues. (D) PCa stemness genes were found to be highly expressed in all tumor samples.

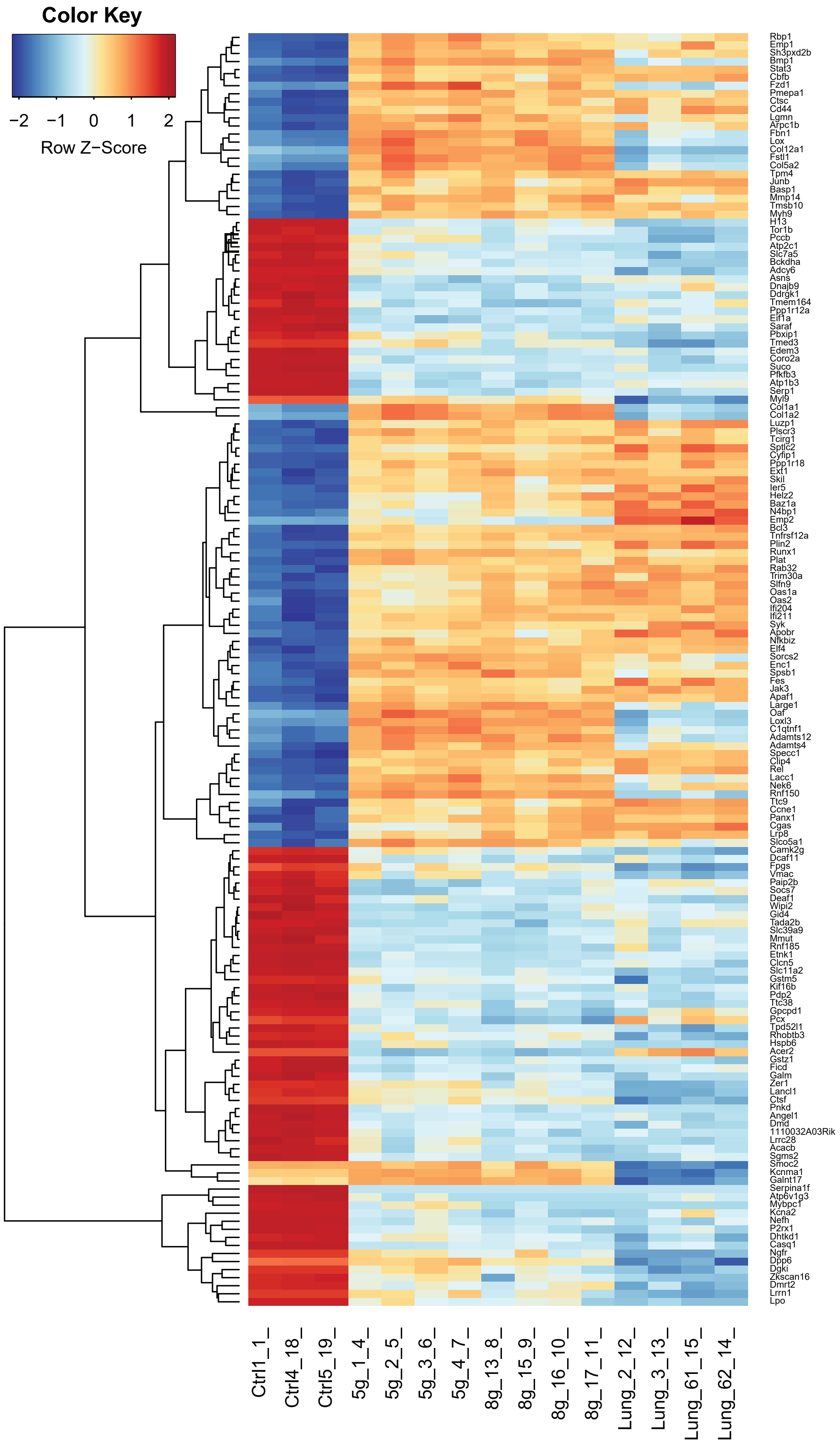

Color Key
−2 −1 0 1 2
Row Z−Score
Rbp1
Emp1
Sh3pxd2b
Bmp1
Stat3
Cbfb
Fzd1
Pmepa1
Ctsc
Cd44
Lgmn
Arpc1b
Fbn1
Lox
Col12a1
Fstl1
Col5a2
Tpm4
Junb
Basp1
Mmp14
Tmsb10
Myh9
H13
Tor1b
Pccb
Atp2c1
Slc7a5
Bckdha
Adcy6
Asns
Dnajb9
Ddrgk1
Tmem164
Ppp1r12a
Eif1a
Saraf
Pbxip1
Tmed3
Edem3
Coro2a
Suco
Pfkfb3
Atp1b3
Serp1
Myl9
Col1a1
Col1a2
Luzp1
Plscr3
Tcirg1
Sptlc2
Cyfip1
Ppp1r18
Ext1
Skil
Ier5
Helz2
Baz1a
N4bp1
Emp2
Bcl3
Tnfrsf12a
Plin2
Runx1
Plat
Rab32
Trim30a
Slfn9
Oas1a
Oas2
Ifi204
Ifi211
Syk
Apobr
Nfkbiz
Elf4
Sorcs2
Enc1
Spsb1
Fes
Jak3
Apaf1
Large1
Oaf
Loxl3
C1qtnf1
Adamts12
Adamts4
Specc1
Clip4
Rel
Lacc1
Nek6
Rnf150
Ttc9
Ccne1
Panx1
Cgas
Lrp8
Slco5a1
Camk2g
Dcaf11
Fpgs
Vmac
Paip2b
Socs7
Deaf1
Wipi2
Gid4
Tada2b
Slc39a9
Mmut
Rnf185
Etnk1
Clcn5
Slc11a2
Gstm5
Kif16b
Pdp2
Ttc38
Gpcpd1
Pcx
Tpd52l1
Rhobtb3
Hspb6
Acer2
Gstz1
Ficd
Galm
Zer1
Lancl1
Ctsf
Pnkd
Angel1
Dmd
1110032A03Rik
Lrrc28
Acacb
Sgms2
Smoc2
Kcnma1
Galnt17
Serpina1f
Atp6v1g3
Mybpc1
Kcna2
Nefh
P2rx1
Dhtkd1
Casq1
Ngfr
Dpp6
Dgki
Zkscan16
Dmrt2
Lrrn1
Lpo
Ctrl1_1_
Ctrl4_18_
Ctrl5_19_
5g_1_4_
5g_2_5_
5g_3_6_
5g_4_7_
8g_13_8_
8g_15_9_
8g_16_10_
8g_17_11_
Lung_2_12_
Lung_3_13_
Lung_61_15_
Lung_62_14_

Figure S8: Top dysregulated genes

Heatmap showing the top 100 dysregulated genes between tumor samples and control tissues.

A

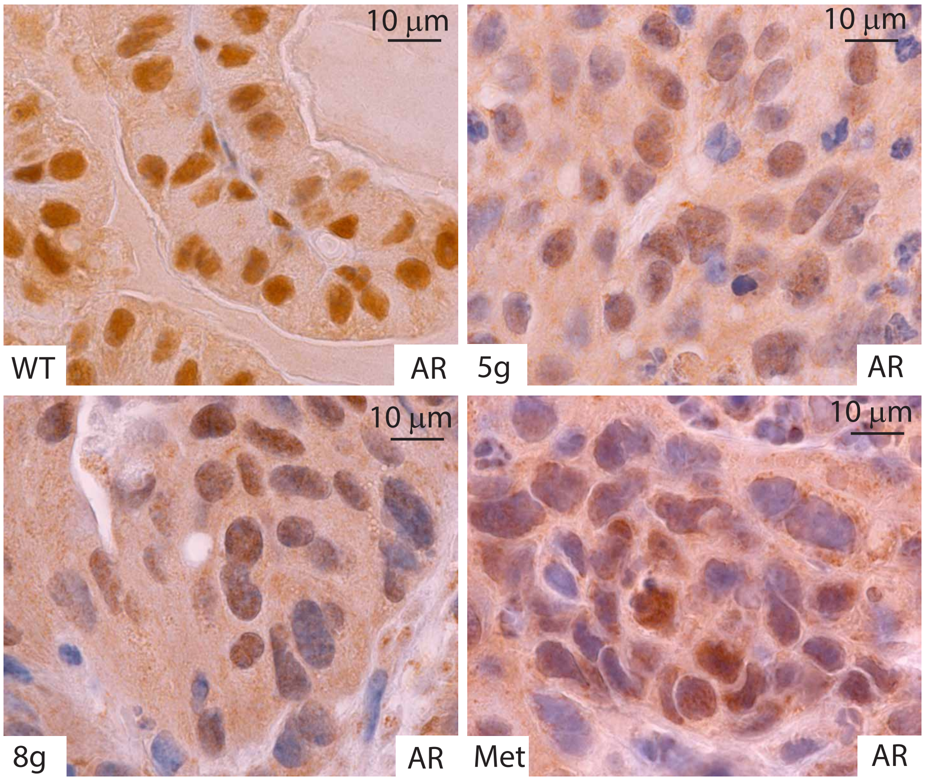

10 µm
WT
AR
10 µm
5g
AR
10 µm
8g
AR
10 µm
Met
AR

B

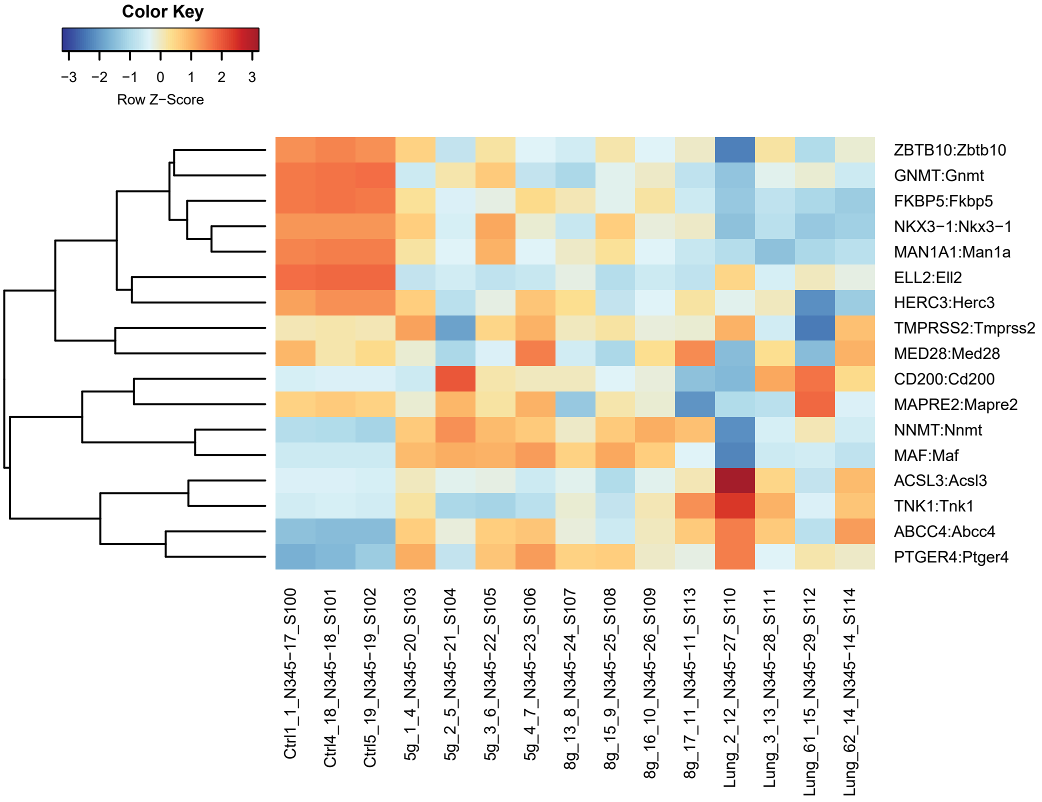

Color Key
−3 −2 −1 0 1 2 3
Row Z−Score
ZBTB10:Zbtb10
GNMT:Gnmt
FKBP5:Fkbp5
NKX3−1:Nkx3−1
MAN1A1:Man1a
ELL2:Ell2
HERC3:Herc3
TMPRSS2:Tmprss2
MED28:Med28
CD200:Cd200
MAPRE2:Mapre2
NNMT:Nnmt
MAF:Maf
ACSL3:Acsl3
TNK1:Tnk1
ABCC4:Abcc4
PTGER4:Ptger4
Ctrl1_1_N345−17_S100
Ctrl4_18_N345−18_S101
Ctrl5_19_N345−19_S102
5g_1_4_N345−20_S103
5g_2_5_N345−21_S104
5g_3_6_N345−22_S105
5g_4_7_N345−23_S106
8g_13_8_N345−24_S107
8g_15_9_N345−25_S108
8g_16_10_N345−26_S109
8g_17_11_N345−11_S113
Lung_2_12_N345−27_S110
Lung_3_13_N345−28_S111
Lung_61_15_N345−29_S112
Lung_62_14_N345−14_S114

Figure S9: Decreased expression of Androgen-regulated genes

A) IHC staining for androgen receptor (AR) on prostatic tissues from sgPten, 5g and 8g tumors together with lung metastases at 8 weeks after cancer initiation. Representative pictures are shown ($n \geq 5$).

B) Expression of AR-regulated genes was assessed between control tissues and the three tumor groups.

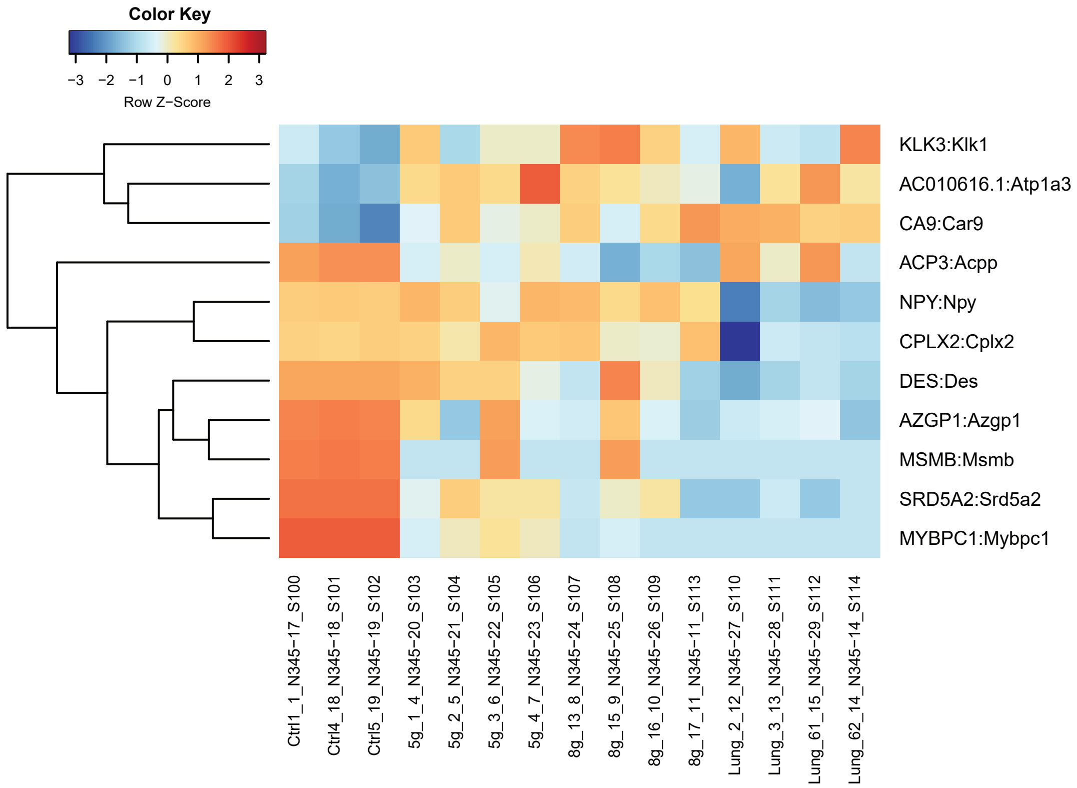

Color Key
−3 −2 −1 0 1 2 3
Row Z−Score
KLK3:Klk1
AC010616.1:Atp1a3
CA9:Car9
ACP3:Acpp
NPY:Npy
CPLX2:Cplx2
DES:Des
AZGP1:Azgp1
MSMB:Msmb
SRD5A2:Srd5a2
MYBPC1:Mybpc1
Ctrl1_1_N345−17_S100
Ctrl4_18_N345−18_S101
Ctrl5_19_N345−19_S102
5g_1_4_N345−20_S103
5g_2_5_N345−21_S104
5g_3_6_N345−22_S105
5g_4_7_N345−23_S106
8g_13_8_N345−24_S107
8g_15_9_N345−25_S108
8g_16_10_N345−26_S109
8g_17_11_N345−11_S113
Lung_2_12_N345−27_S110
Lung_3_13_N345−28_S111
Lung_61_15_N345−29_S112
Lung_62_14_N345−14_S114

Figure S10: Unchanged neuroendocrine signature

Expression of neuroendocrine related genes was assessed between control tissues and the three tumor groups.

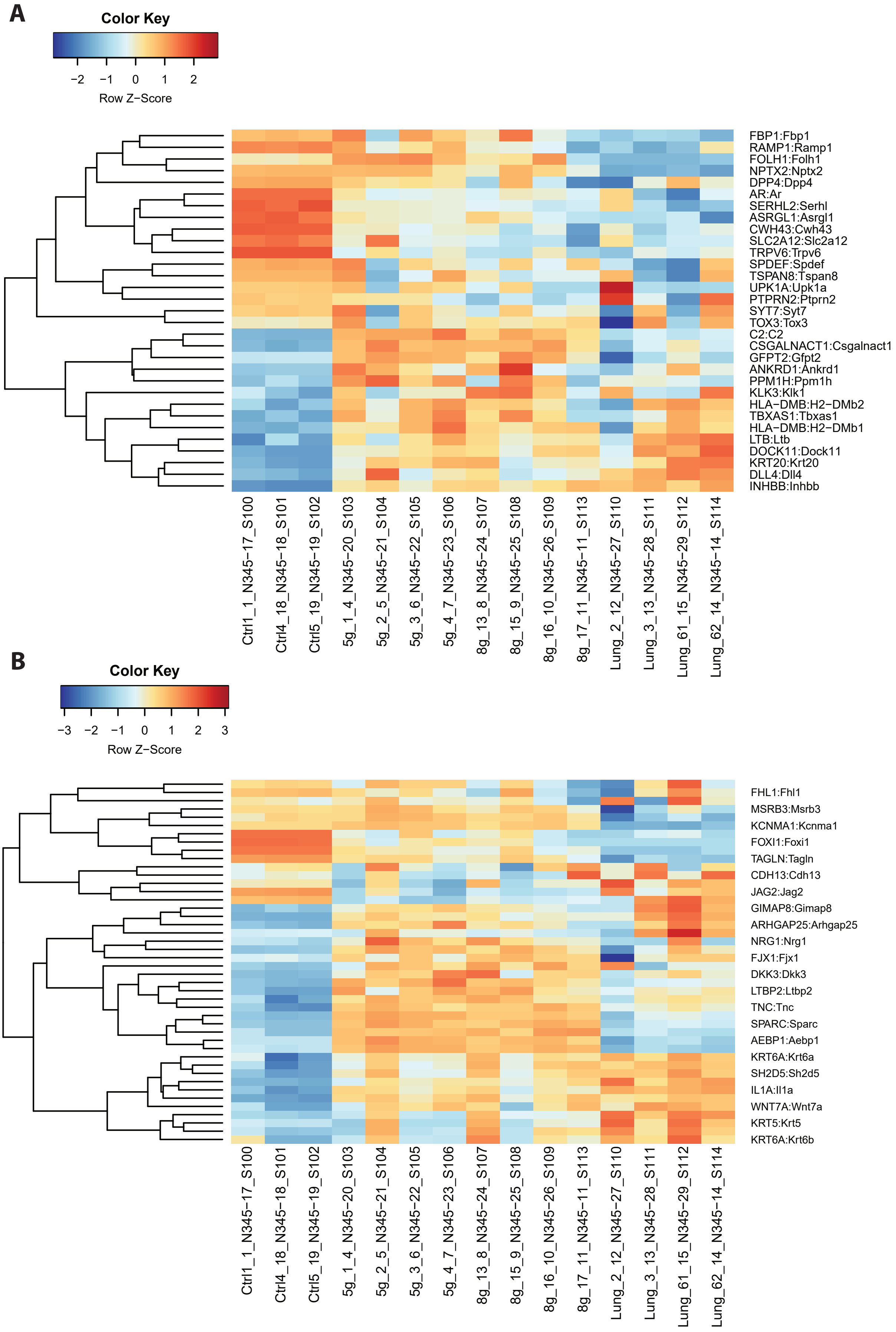
A
Color Key
−2 −1 0 1 2
Row Z−Score
FBP1:Fbp1
RAMP1:Ramp1
FOLH1:Folh1
NPTX2:Nptx2
DPP4:Dpp4
AR:Ar
SERHL2:Serhl
ASRGL1:Asrgl1
CWH43:Cwh43
SLC2A12:Slc2a12
TRPV6:Trpv6
SPDEF:Spdef
TSPAN8:Tspan8
UPK1A:Upk1a
PTPRN2:Ptprn2
SYT7:Syt7
TOX3:Tox3
C2:C2
CSGALNACT1:Csgalnact1
GFPT2:Gfpt2
ANKRD1:Ankrd1
PPM1H:Ppm1h
KLK3:Klk1
HLA−DMB:H2−DMb2
TBXAS1:Tbxas1
HLA−DMB:H2−DMb1
LTB:Ltb
DOCK11:Dock11
KRT20:Krt20
DLL4:Dll4
INHBB:Inhbb
Ctrl1_1_N345−17_S100
Ctrl4_18_N345−18_S101
Ctrl5_19_N345−19_S102
5g_1_4_N345−20_S103
5g_2_5_N345−21_S104
5g_3_6_N345−22_S105
5g_4_7_N345−23_S106
8g_13_8_N345−24_S107
8g_15_9_N345−25_S108
8g_16_10_N345−26_S109
8g_17_11_N345−11_S113
Lung_2_12_N345−27_S110
Lung_3_13_N345−28_S111
Lung_61_15_N345−29_S112
Lung_62_14_N345−14_S114
B
Color Key
−3 −2 −1 0 1 2 3
Row Z−Score
FHL1:Fhl1
MSRB3:Msrb3
KCNMA1:Kcnma1
FOXI1:Foxi1
TAGLN:Tagln
CDH13:Cdh13
JAG2:Jag2
GIMAP8:Gimap8
ARHGAP25:Arhgap25
NRG1:Nrg1
FJX1:Fjx1
DKK3:Dkk3
LTBP2:Ltbp2
TNC:Tnc
SPARC:Sparc
AEBP1:Aebp1
KRT6A:Krt6a
SH2D5:Sh2d5
IL1A:Il1a
WNT7A:Wnt7a
KRT5:Krt5
KRT6A:Krt6b
Ctrl1_1_N345−17_S100
Ctrl4_18_N345−18_S101
Ctrl5_19_N345−19_S102
5g_1_4_N345−20_S103
5g_2_5_N345−21_S104
5g_3_6_N345−22_S105
5g_4_7_N345−23_S106
8g_13_8_N345−24_S107
8g_15_9_N345−25_S108
8g_16_10_N345−26_S109
8g_17_11_N345−11_S113
Lung_2_12_N345−27_S110
Lung_3_13_N345−28_S111
Lung_61_15_N345−29_S112
Lung_62_14_N345−14_S114

Figure S11: Altered basal/luminal signature in tumor samples

Expression of basal/luminal genes was assessed between control tissues and the three tumor groups. (A) luminal signature (B) basal signature.

A

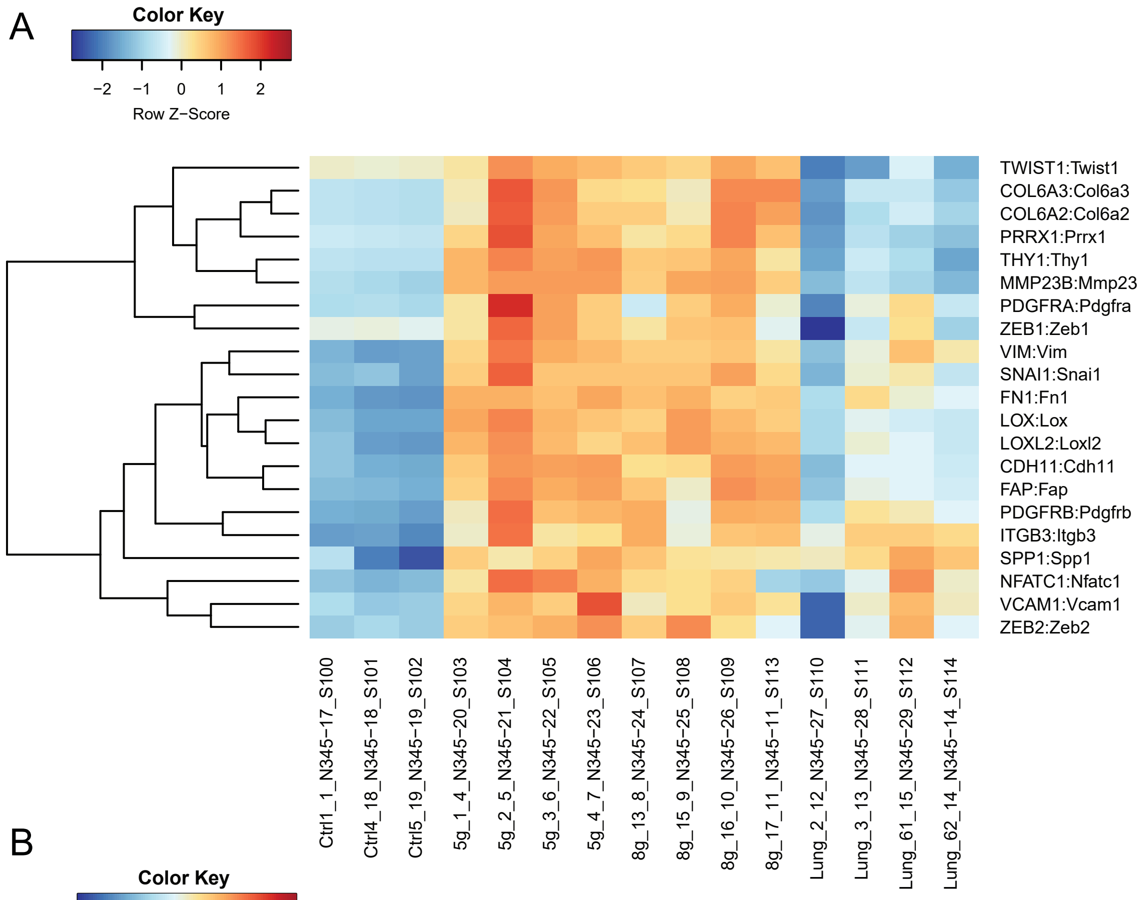
Color Key
−2 −1 0 1 2
Row Z−Score
TWIST1:Twist1
COL6A3:Col6a3
COL6A2:Col6a2
PRRX1:Prrx1
THY1:Thy1
MMP23B:Mmp23
PDGFRA:Pdgfra
ZEB1:Zeb1
VIM:Vim
SNAI1:Snai1
FN1:Fn1
LOX:Lox
LOXL2:Loxl2
CDH11:Cdh11
FAP:Fap
PDGFRB:Pdgfrb
ITGB3:Itgb3
SPP1:Spp1
NFATC1:Nfatc1
VCAM1:Vcam1
ZEB2:Zeb2
Ctrl1_1_N345−17_S100
Ctrl4_18_N345−18_S101
Ctrl5_19_N345−19_S102
5g_1_4_N345−20_S103
5g_2_5_N345−21_S104
5g_3_6_N345−22_S105
5g_4_7_N345−23_S106
8g_13_8_N345−24_S107
8g_15_9_N345−25_S108
8g_16_10_N345−26_S109
8g_17_11_N345−11_S113
Lung_2_12_N345−27_S110
Lung_3_13_N345−28_S111
Lung_61_15_N345−29_S112
Lung_62_14_N345−14_S114

B

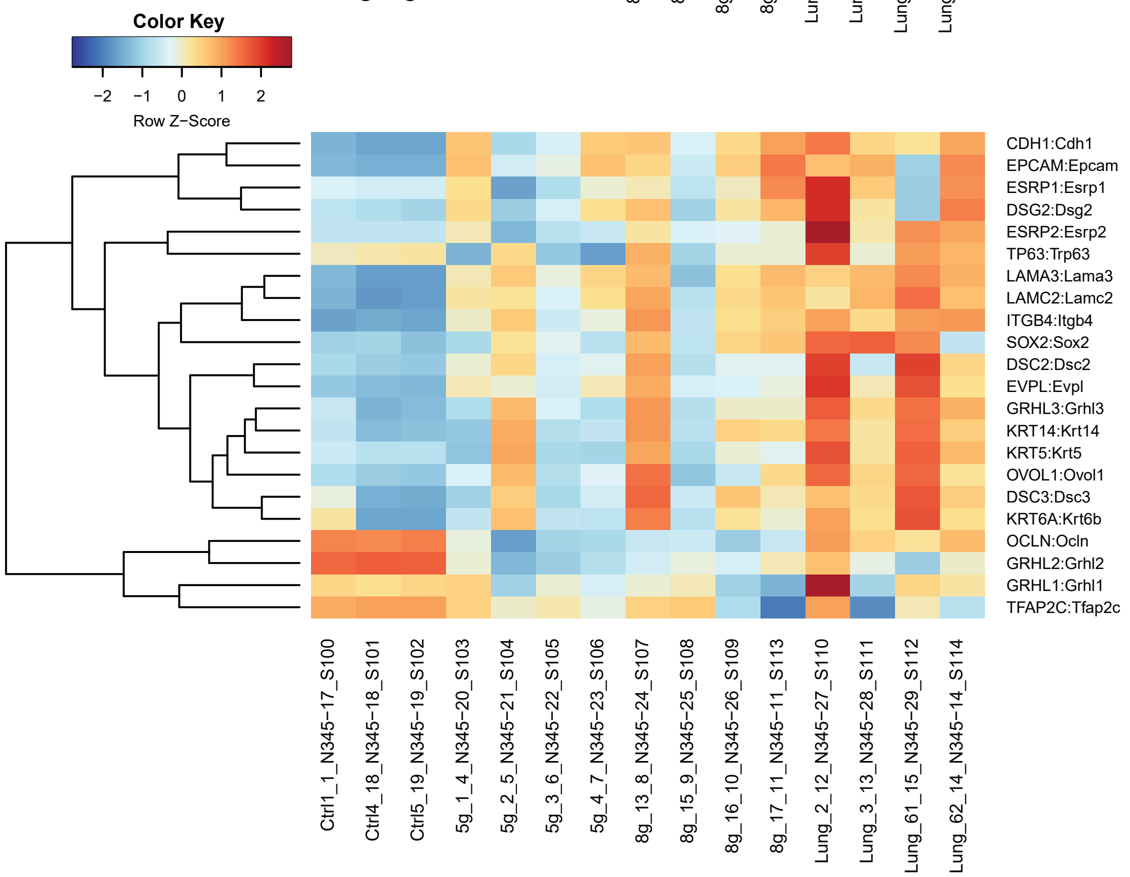
Color Key
−2 −1 0 1 2
Row Z−Score
CDH1:Cdh1
EPCAM:Epcam
ESRP1:Esrp1
DSG2:Dsg2
ESRP2:Esrp2
TP63:Trp63
LAMA3:Lama3
LAMC2:Lamc2
ITGB4:Itgb4
SOX2:Sox2
DSC2:Dsc2
EVPL:Evpl
GRHL3:Grhl3
KRT14:Krt14
KRT5:Krt5
OVOL1:Ovol1
DSC3:Dsc3
KRT6A:Krt6b
OCLN:Ocln
GRHL2:Grhl2
GRHL1:Grhl1
TFAP2C:Tfap2c
Ctrl1_1_N345−17_S100
Ctrl4_18_N345−18_S101
Ctrl5_19_N345−19_S102
5g_1_4_N345−20_S103
5g_2_5_N345−21_S104
5g_3_6_N345−22_S105
5g_4_7_N345−23_S106
8g_13_8_N345−24_S107
8g_15_9_N345−25_S108
8g_16_10_N345−26_S109
8g_17_11_N345−11_S113
Lung_2_12_N345−27_S110
Lung_3_13_N345−28_S111
Lung_61_15_N345−29_S112
Lung_62_14_N345−14_S114

Figure S12: Altered expression of epithelial and mesenchymal signature genes

Expression of epithelial and mesenchymal genes was assessed between control tissues and the three tumor groups. (A) mesenchymal signature (B) epithelial signature.

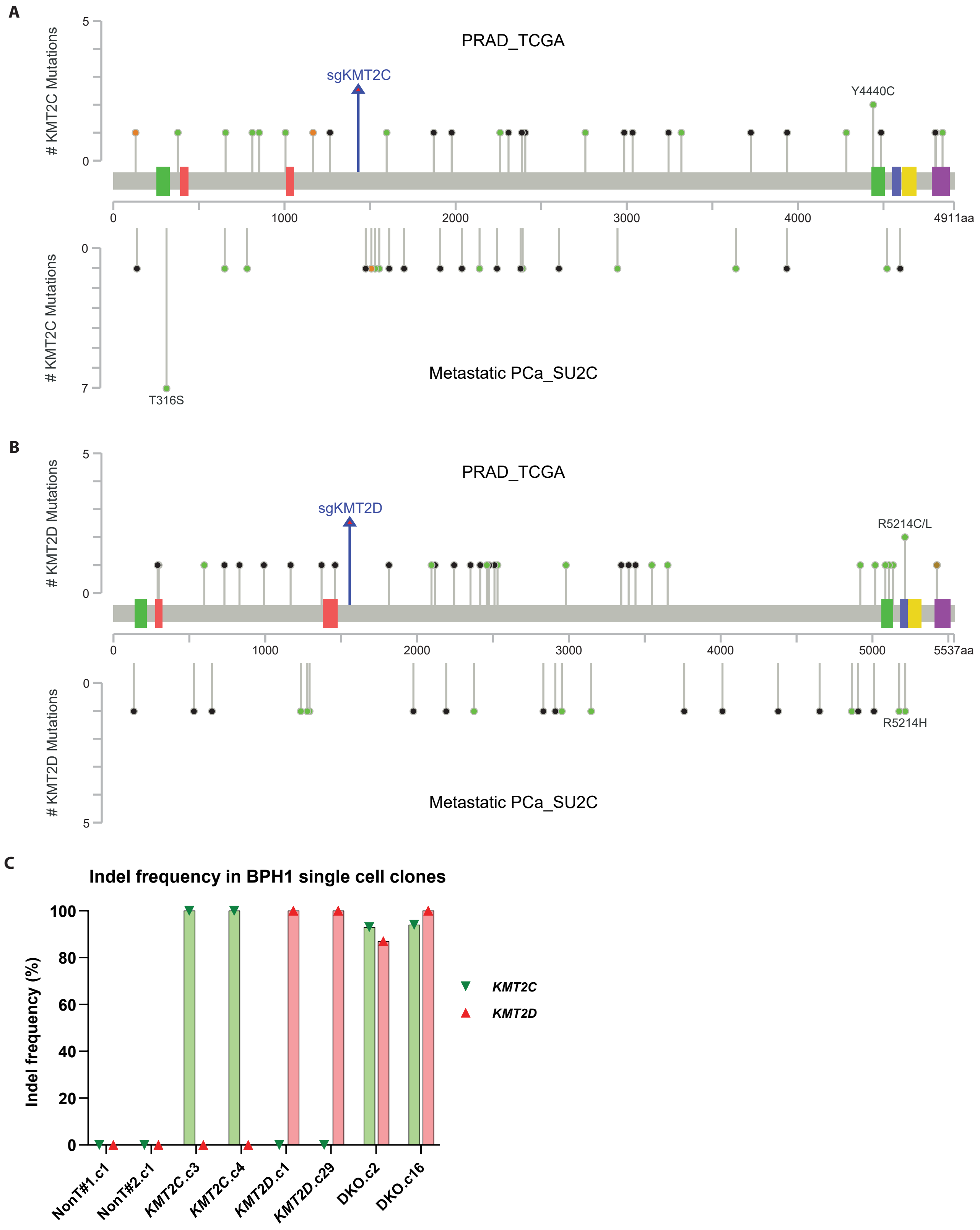

A
PRAD_TCGA
sgKMT2C
Y4440C
KMT2C Mutations
4911aa
T316S
Metastatic PCa_SU2C
B
PRAD_TCGA
sgKMT2D
R5214C/L
KMT2D Mutations
5537aa
R5214H
Metastatic PCa_SU2C
C
Indel frequency in BPH1 single cell clones
Indel frequency (%)
KMT2C
KMT2D
NonT#1.c1
NonT#2.c1
KMT2C.c3
KMT2C.c4
KMT2D.c1
KMT2D.c29
DKO.c2
DKO.c16

Figure S13: Mutation profile of KMT2C and KMT2D in human datasets and CRIPSR induces mutation to BPH1 cells.

(A, B) Mutation profile of KMT2C or KMT2D in primary PCa (up panel) and SU2C-derived metastatic PCa datasets (down panel). The blue arrow indicated the approximate amino acids (AA) locations targeted by CRISPR/Cas9 guide for KMT2C or KMT2D. (C). Indel frequencies for KMT2C and KMT2D in BPH1 cell clones mutated by CRISPR/Cas9.

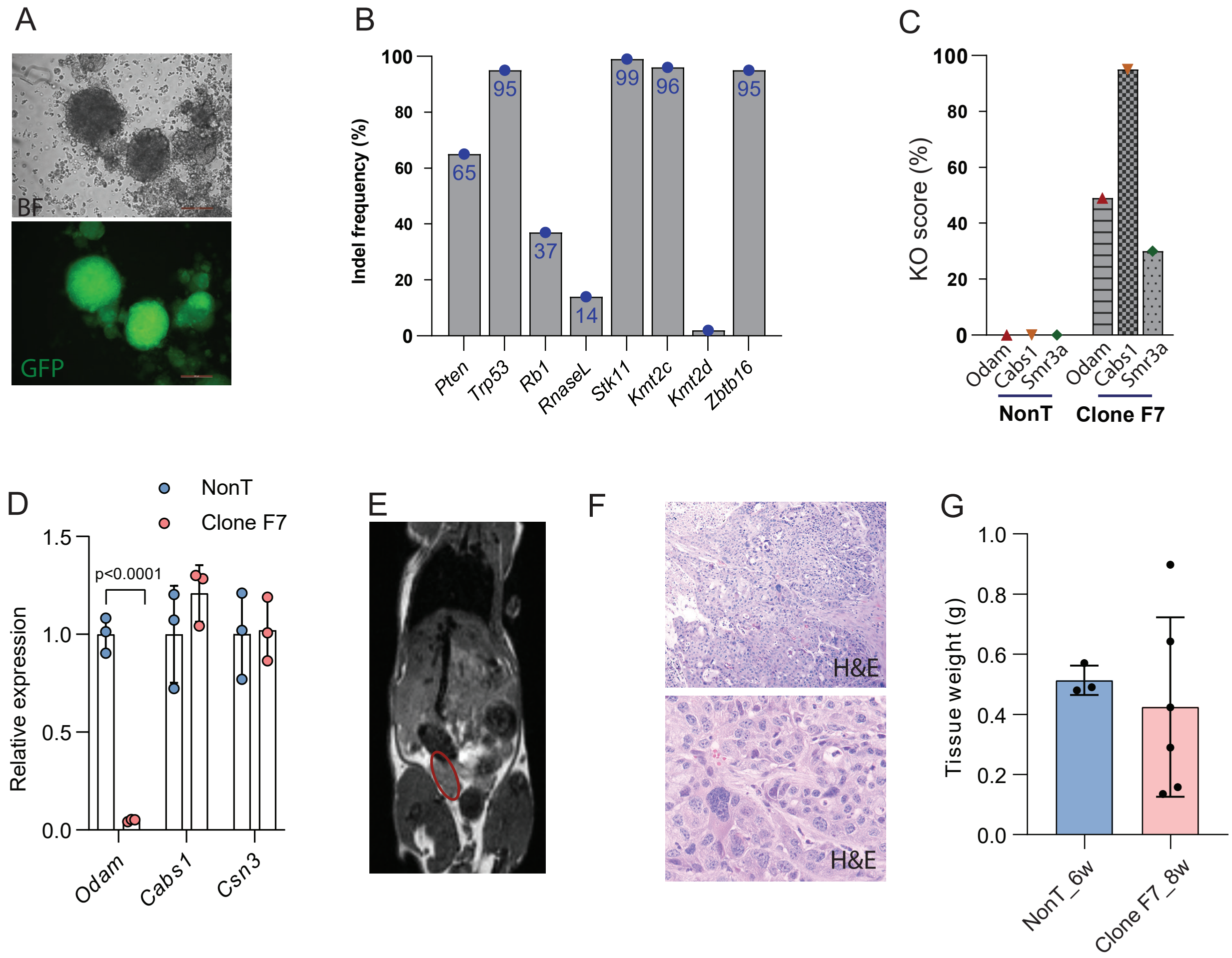

A
BF
GFP
B
Indel frequency (%)
Pten
Trp53
Rb1
RnaseL
Stk11
Kmt2c
Kmt2d
Zbtb16
65
95
37
14
99
96
95
C
KO score (%)
Odam
Cabs1
Smr3a
NonT
Clone F7
D
NonT
Clone F7
Relative expression
p<0.0001
Odam
Cabs1
Csn3
E
F
H&E
H&E
G
Tissue weight (g)
NonT_6w
Clone F7_8w

Figure S14: Disruption of Odam and Cabs1 impairs metastatic formation.

A) Representative pictures of a lung metastatic cell line derived from 8g PCa. B) The indel frequency for the eight target genes in the established cell line. C) KO score of the four target genes at the mouse chr5qE1 for clone F7. D) mRNA expression of three target genes in the control cells and clone F7 (n=3). E) MRi scanning of mice 6 weeks after orthotopically implantation of F7 cells into the prostate. The red dotted line marks tumor formation in the prostatic lobe. A representative picture is shown (n=4). F) H&E staining of the primary PCa 6 weeks after implantation. Representative pictures are shown (n=3). G) Weight of the primary tumor for control cells 6 weeks after implantation and for clone F7 at 8 weeks after implantation (n=3,6). Source data are provided as a Source Data file.

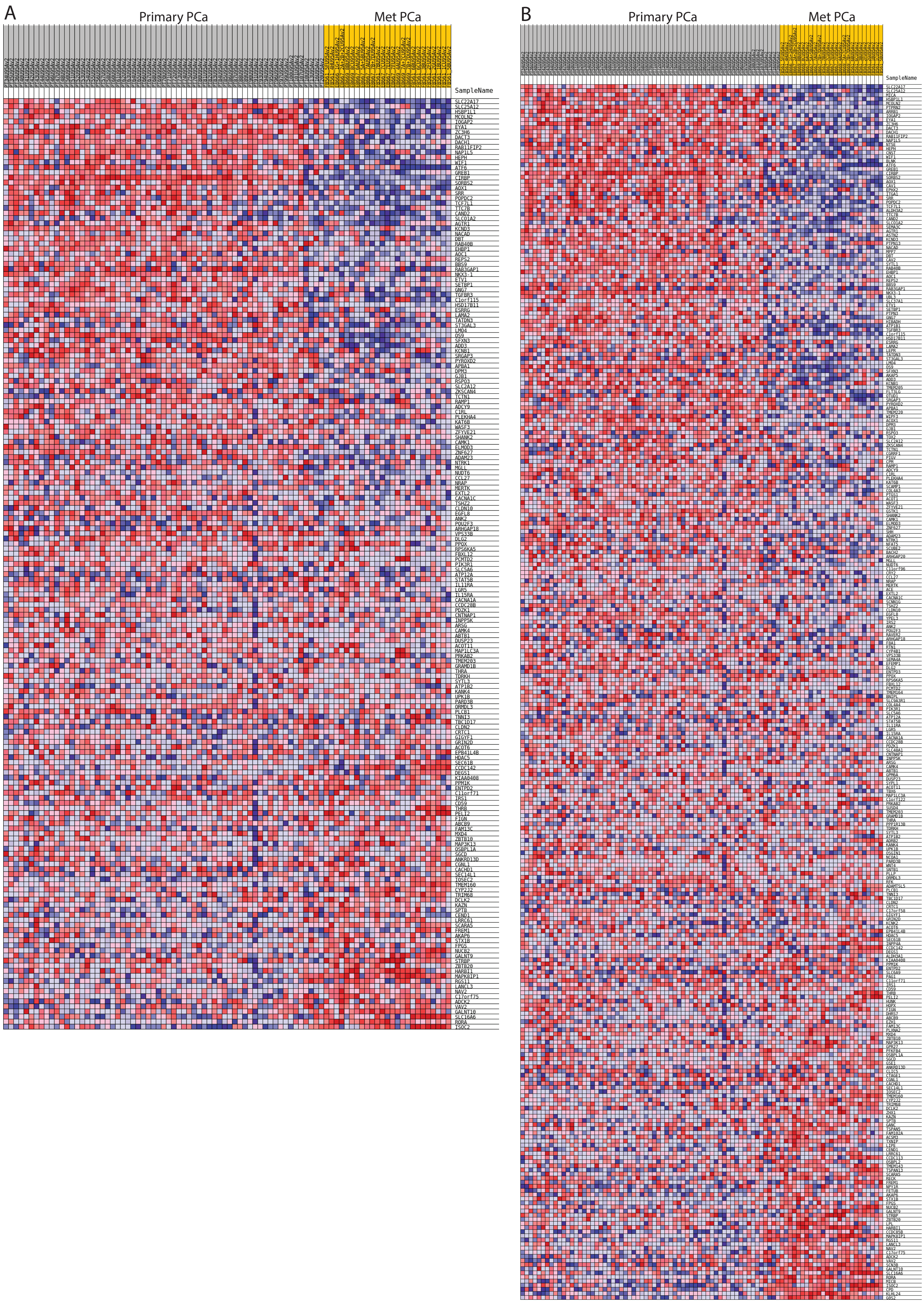

A
Primary PCa
Met PCa
SampleName
B
Primary PCa
Met PCa
SampleName

Figure S15: A gene signature was found based on 8g primary tumor alone.

A) The down-regulated genes (n=240), commonly shared by 8g primary and secondary tumor groups, can distinguish human patients with metastases from patients with primary tumors (GSE35988, n=54, 35). B) The down-regulated genes (n=394) of 8g-derived primary tumor but not present in 5g tumors differentiate human PCa into primary and secondary tumors (GSE35988, n=54, 35).

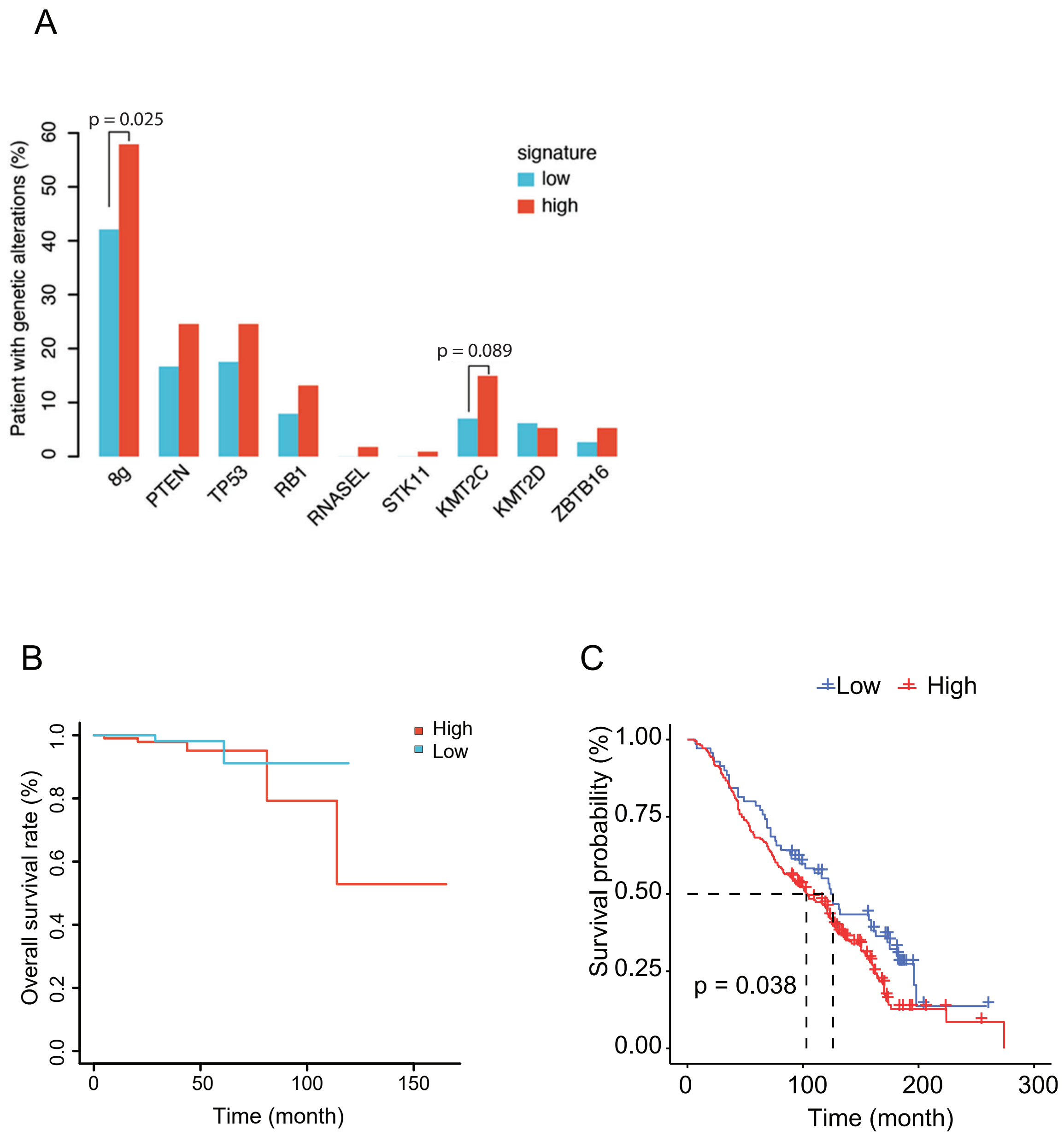
A
p = 0.025
p = 0.089
Patient with genetic alterations (%)
0
10
20
30
40
50
60
signature
low
high
8g
PTEN
TP53
RB1
RNASEL
STK11
KMT2C
KMT2D
ZBTB16
B
High
Low
Overall survival rate (%)
0.0
0.2
0.4
0.6
0.8
1.0
0
50
100
150
Time (month)
C
Low
High
Survival probability (%)
1.00
0.75
0.50
0.25
0.00
p = 0.038
0
100
200
300
Time (month)

Figure S16: DEGs between 5g and 8g predict overall survival

(A) Expression of 20 signature genes between 5g and 8g was correlated with mutation statuses for the 8 target genes in the TCGA dataset. Samples were grouped into high/low scores in relation to single mutations or mutations in all 8 genes ($p < 0.025$, hypergeometric test). (B) The 20-gene set from the DEGs between 5g and 8g was used to evaluate overall survival for the TCGA dataset (n=494). (C) A 10-gene panel derived from the DEGs between 5g and 8g tumors was used to evaluate the survival status in the Swedish Watchful Waiting cohort (GSE16560, n=280).
