# Supplementary material for: CRISPR/Cas9 model of prostate cancer identifies Kmt2c deficiency as a metastatic driver by Odam/Cabs1 gene cluster expression
Source: Nat Commun. 2024 Mar 7;15:2088. doi: 10.1038/s41467-024-46370-0 (PMC10920892; doi:10.1038/s41467-024-46370-0)
Supplement: Supplementary file 3 — Reporting Summary [file 41467_2024_46370_MOESM3_ESM.pdf]

Description of Additional Supplementary Files

File Name: Supplementary Data 1

Description: List of primers and sgRNA

File Name: Supplementary Data 2

Description: WGS analysis of Off-target and exonic mutations

File Name: Supplementary Data 3

Description: WGS Mean Base Quality

File Name: Supplementary Data 4

Description: WGS Mean Mapping Quality

File Name: Supplementary Data 5

Description: Differentially expressed genes

File Name: Supplementary Data 6

Description: GSEA analysis for 50 cancer-related hallmarks
