# Supplementary material for: CRISPR/Cas9 model of prostate cancer identifies Kmt2c deficiency as a metastatic driver by Odam/Cabs1 gene cluster expression
Source: Nat Commun. 2024 Mar 7;15:2088. doi: 10.1038/s41467-024-46370-0 (PMC10920892; doi:10.1038/s41467-024-46370-0)
Supplement: Supplementary file 5 — Supplementary Data 1-6 [file 41467_2024_46370_MOESM5_ESM.pdf]

nature portfolio

Peer Review File

CRISPR/Cas9 model of prostate cancer identifies Kmt2c deficiency as a metastatic driver by Odam/Cabs1 gene cluster expression

Open Access This file is licensed under a Creative Commons Attribution 4.0 International License, which permits use, sharing, adaptation, distribution and reproduction in any medium or format, as long as you give appropriate credit to the original author(s) and the source, provide a link to the Creative Commons license, and indicate if changes were made. In the cases where the authors are anonymous, such as is the case for the reports of anonymous peer reviewers, author attribution should be to 'Anonymous Referee' followed by a clear attribution to the source work. The images or other third party material in this file are included in the article’s Creative Commons license, unless indicated otherwise in a credit line to the material. If material is not included in the article’s Creative Commons license and your intended use is not permitted by statutory regulation or exceeds the permitted use, you will need to obtain permission directly from the copyright holder. To view a copy of this license, visit http://creativecommons.org/licenses/by/4.0/.

REVIEWER COMMENTS

Reviewer #1 (Remarks to the Author):

The authors elucidated the induction of primary and nonmetastatic prostate cancer through the mutation of 5 tumor suppressors (Pten, Trp53, Rb1, Stk11, and RnaseL) in mice. Furthermore, they also demonstrated that an additional mutation in Kmt2c leads to the formation of metastases. Additionally, their results also highlighted the crucial role of genes within the chr5qE1 genomic region in metastasis, as evidenced by their regulation of Kmt2c. Moreover, the metastasis ability of prostate cancer cell lines with Kmt2c mutation is compromised by mutations in Odam and Cabs1. Overall, this manuscript is well written and the analysis is reasonably presented. However, suggested experiments and edits can further enhance its quality:

Major:
1. The mutation profile shown in Figure S1B reveals that STK11 and RNaseL are only mutated in a small fraction of human prostate tumors, accounting for 0.4% and 0.6 respectively. This raises concerns regarding the rationale behind their inclusion in the study. Notably, one tumor exhibited an absence of mutations in both of these genes, further challenging the initial hypothesis to incorporate them.
2. The utilization of Sanger sequencing for indel analysis, as demonstrated in the Figure 1E, Figure 2E, Figure 5F, and 5G may prove inadequate for the identification of infrequent indels. To comprehensively detect and analyze indels within the tissues, targeted NGS is recommended.
3. In Figure 3A and B, the utilization of WGS to identify an off-target of Trp53 sgRNA, while not detecting the Trp53 on-target indel within the same lung metastases, indicating WGS is not sufficient for indel detection. To supplement this observation, targeted NGS is recommended.
4. On page 14, they stated that "the CRISPR induced mutations are sufficient to drive cancer formation and metastatic seeding but also facilities genetic instability resulting in heterogeneous tumors." "genetic instability resulting in heterogeneous tumors" lacks empirical backing. It is recommended to rephrase this statement to a more cautious tone or consider omitting this aspect altogether.

Minor:
1. Please provide the full names for the abbreviations "DLVP", "AP" in Figure 1B, "NEPC" in Figure 4F.
2. On page 13, they stated "Furthermore, mice with loss in only Pten, Trp53 and Rb1 in combination with loss of Kmt2c and Kmt2d had slower tumor progression, revealing that loss of Stk11 and RnaseL accelerated tumor development (Fig S3)." It is assumed that the reference is to Figure S3 G-I. However, these figures appear to be absent. Kindly reinsert Figures S3 G-I to support this statement.
3, On page 17, The intended sentence may be: "Overall, loss of KMT2C in PCa increased the expression of a conserved gene cluster, which is essential for tumor growth and the formation of metastasis."
4, a Western blot analysis is recommended to ascertain the knockout status of Odam and Cabs1 in the clones used in the study. This would provide definitive confirmation of the gene knockout and enhance the reliability of the findings.

Reviewer #2 (Remarks to the Author):

Questions:
How does creating multiple mutations at one effect the cell? Is there genomic instability? How clinically relevant does it make this since any temporal phenotype is lost.

Why inject into the anterior lobe of the prostate? It is known that the dorsolateral most resembles the human prostate.

I would like to understand more why STK11 (0.4%) and RNASEL (0.6%) were chosen when their incidence is so low (Fig S1). Does the survival cure change if they are removed?

Other than KMT2, did you look at other factors that may modify the chromatin state?

Reviewer #3 (Remarks to the Author):

In this extensive work, Cai and colleagues focus on understanding the contribution of epigenetic factors altering chromatin modifications on the onset of metastatic prostate cancer. Specifically, by introducing a pool of 3 specific mutations in KMT2C, KMT2D and ZBTB16 genes homologous in mice, in addition to a pool of 5 CRSPR RNA guided deletions for Pten, Trp53, Rb1, Stk11 and RnaseL in the anterior lobe prostate epithelial cells of immunocompetent mice, they discover that the 8g mice (mutated in Kmt2c, Kmt2d and Zbtb16 plus Pten, Trp53, Rb1, Stk11 and RnaseL) develop metastatic prostate cancer after 8 weeks as opposed to 5g mice (mutated in Pten, Trp53, Rb1, Stk11 and RnaseL only) that develop prostate cancer but do not develop metastases.
The authors further show that a region epigenetically regulated by KMT2C in chr5qE1 harbours gene potentially responsible for mediating the increased metastatic potential in 8g and that has a organotropism to the lungs.
This work provide an explanation of the role of the KMT2C mutations in contributing to metastatic prostate cancer. However, the model seems to be independent of AR and therefore rendering convoluted to set the clinical scenario in which such range of alterations would contribute to patients outcome.
Though findings are promising and have the potential to guide our understanding of the molecular underpinnings of metastatic prostate cancer, the approaches are bit expansive, with too many targets and genetic combinations to be able to determine the essential genetic alterations and transcriptional signatures promoting metastasis. If the authors are able to narrow down the specific genes and their targets that drive metastasis this will make a much more impactful study. Since KTM2C was already shown by another group to drive metastasis with Pten loss, the novelty here is in identifying Odam and Cabs1 as drivers downstream of KTM2c and the authors should focus their efforts here.

Some specific comments
1. Fig. S1B: STK11 and RNASEL alterations are quite rare. Also, there is no patient where all 5 alterations occur simultaneously. As such it is unclear the rationale for targeting these in their model and its translational relevance.

2. The rational for mutating also Stk11 and RnaseL is revealed in line 9 page 13 and Figure S3.: The mice developed tumors more rapidly. However the contribution of Stk11 and RnaseL alterations to PFS is minimal compared to Pten, Trp53, and Rb1 (Figure S1). Repeating the analysis without the 2 affected cases will not reveal dramatic changes in PFS. This should be further emphasised in abstract, and discussion.

3. Fig. S3: Data showing Pten, Rb1, p53 loss combined with loss of Kmt2c or Kmt2d is not shown and needs to be included. Data showing Pten loss alone with KMT2C loss is also not shown but referenced in the Discussion on Page 19.

4. Fig. 1E. Difficult to say if reduced indel frequency for RnaseL and STK11 is a consequence of reduced CRISPR cutting efficiencies or a biological consequence of loss of these genes having no or even a negative selective pressure in prostate cancer development. Indeed, RNASEL can also be amplified in human prostate cancer (fig. S1). To address these possibilities, the authors should first show CRISPR indel frequency in vitro to assess cutting efficiency. As indel frequency does not full equate with protein loss, the authors should show protein expression by IHC/IF or Western blots to confirm the degree of protein loss in vivo. Finally, they should generate tumor models with just the deletion of Pten, p53, Rb1 and compare tumor onset and severity to 5g model to definitely assess role of RNASEL and STK11 loss in prostate cancer development.

5. Again comparing Figure 1E , 2E and 4A, one would notice that the frequency of indels in the 5g is high only in Stk11, and this remains high in 8g, at the same level of Kmt2c, Kmt2d and Zbtb16 in 8g. Moreover the frequency of indels in 8g seems higher than in 5g. How can the authors rule out that is rather the differential efficiency of the vectors in generating mutations that can cause the different metastatic potential?

6. Fig. 3: Did the authors assess Copy number changes? For instance, MYC is commonly amplified and has been shown to drive metastasis. CNAs have been shown to be much more common in metastatic prostate cancer than mutations, which occur generally at a low frequency. That being said, the mutational rate in these metastases is unusually high. How does this compare to the mutational rate in the primary tumors? Comparing primary vs. metastatic tumors will be necessary to conclude that acquisition of additional mutations during tumor evolution is not a possible driver of metastasis in their model.

7. Overall: Because there are so many genes targeted in the 8g setting, it is difficult to determine the essential genetic alterations and transcriptional signatures promoting metastasis. The authors need to narrow down the essential TSG losses that are sufficient for primary tumor growth, castration resistance, and metastasis, and then analyze those samples transcriptionally to identify the essential genes and their targets necessary for metastasis. Given their findings, this would include p53, Pten, Rb1, and KMT2c, who's losses were present at high frequency in both primary and metastatic samples.

8. Fig. 1G. The authors should stain for AR expression and expression of CK5 and CK8. Based on p63 staining it appears that tumor cells start express high levels of basal markers in the 5g setting, suggesting basal differentiation. The authors should comment on this.

9. The castration of mice (fig 2C-D) revealed quite a bit of variability in reducing tumor weight as compared to intact mice (Fig 2C and 1J) but the mice reached the humane endpoint at the same time as the intact mice (Fig 2D and 1H). Given the imperfect CRSPR/Cas9 activity (Fig 1E), it would be worth to examine the dependence of AR of such tumors. Comparing castration in 5g and 8g and staining these for AR and AR target genes would also be of relevance. Does this newly generated model depend on AR at all ? The IHC analysis in S4A present weak cytoplasmic staining, similar to what is stated about Fig 4G-H, but the staining intensity is not quantified in the various groups. higher magnification images of the staining should be provided. The dependency of these tumors from AR should be clarified. If not rather these factors being important in progression of PC to CRPC, since androgen deprivation (castration) is the main stay treatment for primary PC patients, this model rather represent a possible genetic make up for high risk prostate cancer prone to develop metastases.

10. Figure 3 and Figure S5 deserves a little more attention. Figure 3F shows that additional mutations were found in all 4 WGsequenced samples but how can the authors exclude that those were not resulting from improper activity of the CRSPR/Cas9 and based on this, how can they attribute the increased metastatic potential in 8g only to the targeted 3 mutated genes? Did I read this correctly that none of the 4 sequenced tumors had mutations in ZBT16 (line 8-9 in page 14). This should be further clarified in the discussion (line 19-31 in page 19) and perhaps more attention should be given to the additional mutations found in the samples.

11. Fig 3D and 4C: The increased mutational burden in 3D and induction in genes related to DNA repair and immune signaling pathways in 4C (IFN, TNF, IL6, IL2) suggest that AAV delivery of CRISPR constructs and/or constitutive Cas9 expression in the prostate epithelium may induce DNA damage and inflammation that may indirectly lead to tumor progression and metastasis. The authors should comment on this possibility or perform additional experiments (for instance without AAV packaging or with transient AAV delivery of Cas9) to rule out these confounding factors as drivers of metastasis.

12. Fig. 5: This is a quite exciting and impactful finding. The authors though could have

alternatively or in addition also use CRISPR to deplete Odam and Cabs1 in vivo in 8g tumors using their AAV approach to validate.

13. Fig. 8D: Expression of Odam and Cabs1 that the authors showed were drivers of metastasis in Fig. 5 are not enhanced in human metastatic disease. The authors should comment and discuss. Moreover, only a handful of DEGs between 5g and 8g are altered in human metastatic disease and are not associated with differences in overall survival (Fig. S9b). Again, the authors should narrow down the list of genes and targets that are drivers of metastasis in their mouse model and then determine their relevance and correlation to human disease.

minor comments
1. fig S6 the gene labels are very small.
2. the word "progression" in the paper is mentioned over 40 times to indicate that a primary untreated tumor develops metastasis. this is a little confusing if one thinks that disease progression follows emergence of treatment resistance in the clinical setting. The word progression should be changed of carefully defined as the onset of metastatic disease. e.g. lines 29-31.
3. what is the frequecy of indels in chr5qE1?
4. figure 7: is the frequency of indels for g5+sgKmt2c higher than for g5+sgKmt2d and g5+sgZbtb16? this should be shown in a supplementary figure.
5. fig 8c labels are not readable.
6. Fig. 1B: Unclear if one or both anterior lobes are injected. Can the authors comment on why disease also forms in DLVP if injections occur in anterior lobe?
7. Figure 8E: what is the mutational status of tumors in TCGA PRAD scoring high (or the highest quartile) in respect to Kmt2c homologue? can the authors find any correlation?
8. FigS3 is missing G-I?
9. Fig.4A: "proportion" not propotion
10. Page 17, line 6: Should be KMT2C, not KMT2D
11. Some minor spelling/grammatical errors throughout.

RESPONSE TO REVIEWERS' COMMENTS

We would like to thank the Referees for their constructive criticisms and suggestions, which have improved our manuscript. We have addressed the reviewer comments as detailed below. In brief, changes have been made to the text, especially related to the inclusion of STK11 and RNASEL in the study. Figure 8 has been updated, and 7 new supplementary figures have been added.

REVIEWER COMMENTS

Reviewer #1 (Remarks to the Author):

The authors elucidated the induction of primary and nonmetastatic prostate cancer through the mutation of 5 tumor suppressors (Pten, Trp53, Rb1, Stk11, and RnaseL) in mice. Furthermore, they also demonstrated that an additional mutation in Kmt2c leads to the formation of metastases. Additionally, their results also highlighted the crucial role of genes within the chr5qE1 genomic region in metastasis, as evidenced by their regulation of Kmt2c. Moreover, the metastasis ability of prostate cancer cell lines with Kmt2c mutation is compromised by mutations in Odam and Cabs1. Overall, this manuscript is well written and the analysis is reasonably presented. However, suggested experiments and edits can further enhance its quality:

Major:

1. The mutation profile shown in Figure S1B reveals that STK11 and RNaseL are only mutated in a small fraction of human prostate tumors, accounting for 0.4% and 0.6 respectively. This raises concerns regarding the rationale behind their inclusion in the study. Notably, one tumor exhibited an absence of mutations in both of these genes, further challenging the initial hypothesis to incorporate them.

It is true that the mutation frequency of STK11 and RNaseL in human PCa is relatively low when analyzed in the TCGA dataset. There have been single studies indicating that both factors could have implications in PCa development (see PMID 14583476 and 20576793 for RNaseL and 32219437 and 37573301 for STK11/LBK1), and especially, the loss of STK11 has lately been associated with metastatic PCa. Furthermore, a dataset containing 1013 samples published in Nature Genetics in 2018 (PMID: 29610475) shows a higher incidence of somatic mutations in STK11 (4%) and RNaseL (2.4%). This data has now been added to Figure S1 and S3. Therefore, these two genes were included to assess a possible function in a more complex mouse model with mutations of additional other factors.

Furthermore, we have data where the exclusion of STK11 and RNaseL is delaying tumor development, so the humane endpoint is seen approximately 3 months after initiation of the cancer instead of 2 months (see Figure S4). This indicates that the loss of STK11 and RNaseL promotes cancer development, which we have included in the discussion (see lines 556-563).

As pointed out by the reviewer, samples with intact Stk11 and RNaseL are found in the mouse model. This shows that sub-clones can be formed in the tumor with intact Stk11 and RNaseL. However, in light of the slower tumor progression when these two factors are left out, we interpret that STK11

and RNaseL are implicated in PCa, even though we have not determined their direct function (see lines 548-551).

2. The utilization of Sanger sequencing for indel analysis, as demonstrated in the Figure 1E, Figure 2E, Figure 5F, and 5G may prove inadequate for the identification of infrequent indels. To comprehensively detect and analyze indels within the tissues, targeted NGS is recommended.

We agree with the reviewer that Sanger sequencing can be inadequate for low (<5%) or high (>80%) indel frequency, as the method is PCR-based and compares the target sequence to a wild-type PCR control. In Figure 5F, where a high KO score is expected in cell clones, the indel efficacy of approximately 90% can be an underestimation. However, it can also be due to the presence of a few unedited cells. We re-sequenced the two cell clones at a later passage number and obtained the same indel frequency. This indicates that there is no genetic drift in the clones, and it is possible that these two clones are fully knocked out. This is also confirmed by WB analysis, which has been added to the new Figure S2.

We followed the recommendation from this reviewer to apply targeted NGS for selected samples from 5g and 8g tumors. As both Stk11 and RnaseL have shown wild-type sequences in a subset of samples, this was assessed. The targeted NGS was based on 3 PCR amplification steps to include specific linkers, which may introduce errors. However, we could confirm that intact RnaseL and Stk11 were present in the samples. We have included an example of RnaseL with mutations and an example of a non-mutated sample here:

With indel:

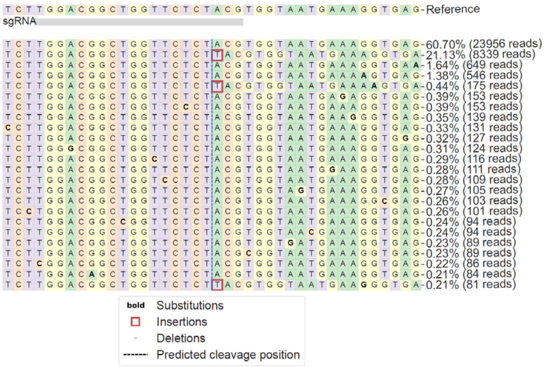

Without indel:

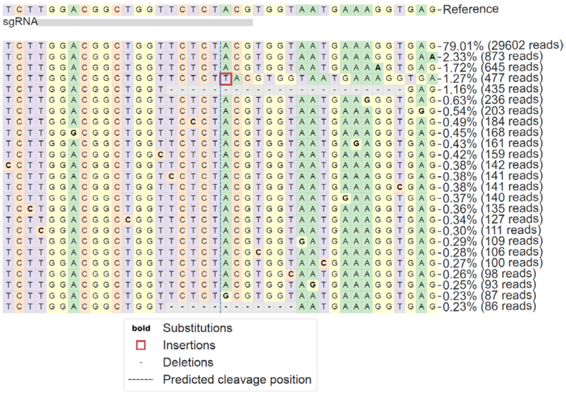

As seen for the sample without indel, 1.27% of the read counts show an insertion at the target site. We predict that this is a PCR error; however, it could also be a sub-clone in the tumor, as multiple clones can be generated by the AAV delivery of the sgRNAs. We are aware that heterogeneity is present in some of the primary tumors and have included that in the discussion (see lines 548-551).

3. In Figure 3A and B, the utilization of WGS to identify an off-target of Trp53 sgRNA, while not detecting the Trp53 on-target indel within the same lung metastases, indicating WGS is not sufficient for indel detection. To supplement this observation, targeted NGS is recommended.

The WGS was done with a read coverage of 40X (96 GB data per sample), and we see an average coverage of around 35 reads. This provides that mutation frequencies under approximately 3% could go undetected. As the tumor cells are at a larger percentage, and we see high mutation frequencies for the other target genes in the WGS samples, we interpret the non-mutated Trp53 as a likely result,

even though it is surprising to see wildtype Trp53 in two metastatic samples. It should be noticed that such samples are metastatic tissues, and tumor evolution and/or selection can occur during the spreading of tumor cells from primary tumor loci. This may also give rise to some metastatic clones with special mutation profiles.

When we obtained the WGS data and found Trp53 non-mutated in 2 samples, we validated the results by Sanger sequencing, which confirmed that no indels were present in Trp53 in these two specific samples (see below). As suggested by the reviewer, we also performed targeted NGS, but unfortunately, the results were inconclusive, as PCR amplification generated many errors, and less than 5% of the reads could be aligned to the reference sequences.

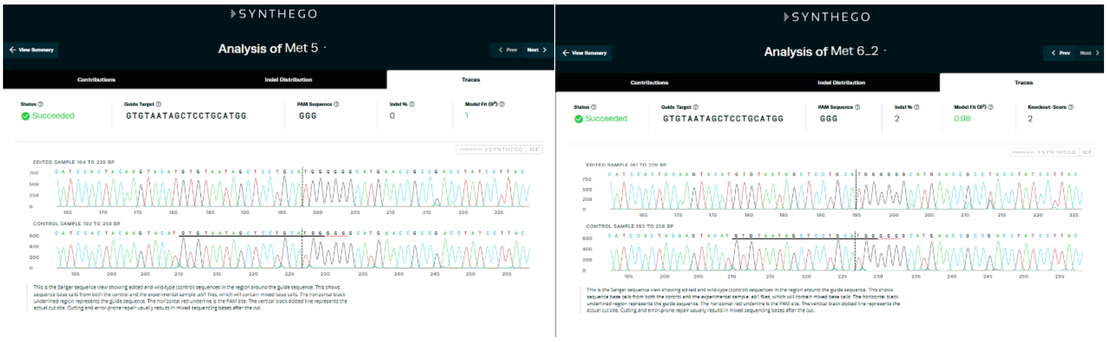

4. On page 14, they stated that "<u>the CRISPR induced mutations are sufficient to drive cancer formation and metastatic seeding but also facilities genetic instability resulting in heterogeneous tumors.</u>" "<u>genetic instability resulting in heterogeneous tumors</u>" lacks empirical backing. It is recommended to rephrase this statement to a more cautious tone or consider omitting this aspect altogether.

We appreciate the reviewer's comment. Although we observe a low frequency of somatic mutations in coding genes, we do observe frequent mutations at 1.1~1.9 mutations/Mb in the lung metastatic samples. We have now rephrased the statement to: **"This indicates that the specific CRISPR-induced mutations are sufficient to drive cancer formation and metastatic seeding, even though the tumors exhibit genetic instability with a high mutation frequency in intronic regions, resulting in heterogeneous tumors."** (see line 406-408).

Minor:

1. Please provide the full names for the abbreviations "DLVP", "AP" in Figure 1B, "NEPC" in Figure 4F.
   We have added this to the respective figure text.

2. On page 13, they stated "Furthermore, mice with loss in only Pten, Trp53 and Rb1 in combination with loss of Kmt2c and Kmt2d had slower tumor progression, revealing that loss

of Stk11 and RnaseL accelerated tumor development (Fig S3).” It is assumed that the reference is to Figure S3 G-I. However, these figures appear to be absent. Kindly reinsert Figures S3 G-I to support this statement.

We apologize for this mistake as it is also related to the first question raised by this reviewer. The data is now present in figure S4.

3, On page 17, The intended sentence may be: “Overall, loss of KMT2C in PCa increased the expression of a conserved gene cluster, which is essential for tumor growth and the formation of metastasis.”

Yes, this is a mistake, and we appreciate the reviews comment. The sentence has now been corrected.

4, a Western blot analysis is recommended to ascertain the knockout status of Odam and Cabs1 in the clones used in the study. This would provide definitive confirmation of the gene knockout and enhance the reliability of the findings.

We have now performed Western blot for Odam to confirm the loss of protein expression in the generated cell lines. This has been added to the new Figure S2 and commented on at lines 207-209 and 326. Unfortunately, we could not obtain a reliable antibody for Cabs1. The sgRNA for Cabs1 is located at the start of the gene, leading to an early mutation in the reading frame. The analysis by Synthego ICE software predicts a high knockout (KO) score for Cabs1 due to the introduction of a premature stop codon. The KO score for Cabs1 is in Figure 5F.

Reviewer #2 (Remarks to the Author):

Questions:

How does creating multiple mutations at one effect the cell? Is there genomic instability? How clinically relevant does it make this since any temporal phenotype is lost.

Creating multiple mutations by CRISPR has been shown to induce cell death or generate genomic instability. Therefore, we analyzed a subset of the metastatic samples by whole-genome sequencing to identify potential driver mutations that were not intended. Overall, we found approximately 1.1~1.9 mutations/Mb, which is comparable to human metastatic samples (1-3 mutations/Mb) (added to lines 396-398). However, very few common mutations in the coding sequence were found beside the sgRNA targets (total 33 mutations in 4 samples). This indicates that CRISPR induces mutations in the target genes, which were the primary drivers of the cancer. Furthermore, we addressed that there is no copy number amplification of cMyc (see lines 506-508). The clinical relevance is reflected in that our target mutations are common in human PCa or in the pathway connected to the genes. The slowly developing cancer is bypassed in this model; however, this is also the strength of the model as it allows us to address genetic interactions in a timely manner. We also show that the speed of tumor development can be reduced by removing Stk11 and RNaseL1 guides (Figure S4).

Why inject into the anterior lobe of the prostate? It is known that the dorsolateral most resembles the human prostate.

The injection into the anterior lobe is feasible, and it would be very difficult to directly inject into the dorsolateral lobe. However, the virus fluid is distributed to the other lobes (see Riedel et al for details, PMID: 33674748), and we observe a clear high-grade PIN and GFP signal from the dorsolateral lobe at 4 weeks after injection. Additionally, the samples for RNAseq were dissected from the dorsolateral area, including the control tissues. At the humane endpoint, the prostate has lost its structure, but a large tumor mass is seen around the dorsolateral lobe. We have now added this to the methods section at lines 179-180.

I would like to understand more why STK11 (0.4%) and RNASEL (0.6%) were chosen when their incidence is so low (Fig S1). Does the survival cure change if they are removed?

A similar question was raised by the other reviewers. We have copied our answer, and we have now included in the introduction and discussion the rationale behind the selection of two less common genetic alterations in PCa (see lines 67-72 and 556-563).

"It is true that the mutation frequency of STK11 and RNaseL in human PCa is relatively low when analyzed in the TCGA dataset. There have been single studies indicating that both factors could have implications in PCa development (see PMID 14583476 and 20576793 for RnaseL and 32219437 and 37573301 for STK11/LBK1), and, notably, the loss of STK11 has lately been associated with metastatic PCa. Furthermore, a dataset containing 1013 samples published in Nature Genetics 2018 (PMID: 29610475) shows a higher incidence of somatic mutations in STK11 (4%) and RNaseL (2.4%). This data has now been added to Figure S1 and S3. Therefore, these two genes were included to assess a possible function in a more complex mouse model with mutations of additional other factors.

Furthermore, we have data where exclusion of STK11 and RNaseL is delaying tumor development, so humane endpoint is seen approximately 3 months after initiation of the cancer instead of 2 months (see Figure S4). This indicates that loss of STK11 and RNaseL promotes cancer development, which we have included in the discussion (see lines 548-551 and 556-563)."

The survival does not change significantly when we exclude STK11 and RNASEL from the TCGA dataset, as only a few samples are mutated. The dataset from Nature Genetics in 2018 does not contain survival data, so we cannot assess the impact of the loss of STK11 and RNASEL on a larger dataset.

Other than KMT2, did you look at other factors that may modify the chromatin state?

In this study, we also included Kmt2d and Zbtb16, which are both often found mutated in human PCa samples. Our study reveals that neither Kmt2d nor Zbtb16 influences the metastatic potential. However, the loss of Zbtb16 seems to hamper the growth of the primary tumor. We have not investigated the potential molecular mechanism of Zbtb16 loss in this study and what implications it could have on PCa (See Figure 7 for details). We have commented on this in the results section at lines 518-519: 'The primary tumor recaptured the weight of the tumor with only the loss of five tumor suppressor genes, except the loss of Zbtb16, which generated significantly smaller tumors (Figure 7D). And in the discussion at lines 560-563.

Reviewer #3 (Remarks to the Author):

In this extensive work, Cai and colleagues focus on understanding the contribution of epigenetic factors altering chromatin modifications on the onset of metastatic prostate cancer. Specifically, by introducing a pool of 3 specific mutations in KMT2C, KMT2D and ZBTB16 genes homologous in mice, in addition to a pool of 5 CRSPR RNA guided deletions for Pten, Trp53, Rb1, Stk11 and RnaseL in the anterior lobe prostate epithelial cells of immunocompetent mice, they discover that the 8g mice (mutated in Kmt2c, Kmt2d and Zbtb16 plus Pten, Trp53, Rb1, Stk11 and RnaseL) develop metastatic prostate cancer after 8 weeks as opposed to 5g mice (mutated in Pten, Trp53, Rb1, Stk11 and RnaseL only) that develop prostate cancer but do not develop metastases.

The authors further show that a region epigenetically regulated by KMT2C in chr5qE1 harbours gene potentially responsible for mediating the increased metastatic potential in 8g and that has a organotropism to the lungs.

This work provide an explanation of the role of the KMT2C mutations in contributing to metastatic prostate cancer. However, the model seems to be independent of AR and therefore rendering convoluted to set the clinical scenario in which such range of alterations would contribute to patients outcome.

Though findings are promising and have the potential to guide our understanding of the molecular underpinnings of metastatic prostate cancer, the approaches are bit expansive, with too many targets and genetic combinations to be able to determine the essential genetic alterations and transcriptional signatures promoting metastasis. If the authors are able to narrow down the specific genes and their targets that drive metastasis this will make a much more impactful study. Since KTM2C was already shown by another group to drive metastasis with Pten loss, the novelty here is in identifying Odam and Cabs1 as drivers downstream of KTM2c and the authors should focus their efforts here.

Some specific comments

1. Fig. S1B: STK11 and RNASEL alterations are quite rare. Also, there is no patient where all 5 alterations occur simultaneously. As such it is unclear the rationale for targeting these in their model and its translational relevance.

It is true that the mutation frequency of STK11 and RNaseL in human PCa is relatively low when analyzed in the TCGA dataset. There have been individual studies indicating that both factors could have implications in PCa development (see PMID 14583476 and 20576793 for RNaseL and 32219437 and 37573301 for STK11/LBK1). Special loss of STK11 has lately been associated with metastatic PCa. Furthermore, a dataset containing 1013 samples published in Nature Genetics 2018 (PMID: 29610475) shows a higher incidence of somatic mutations in STK11 (4%) and RNaseL (2.4%). This data has now been added to Figure S1 and S3. Therefore, these two genes were included to assess a possible function in a more complex mouse model with mutations in additional factors.

Alterations of all the target genes simultaneously are rare in the same patient samples, but mutations can be present in the involved pathways, as known for p53 and Pten/PI3K pathways. The relevance of STK11 and RNASEL mutations is revealed with slow tumor progression when these two

guides are left out (Figure S4). This indicates that loss of STK11 and RNaseL promotes cancer development, which we have included in the discussion (see lines 545-551 and 556-563).

2. The rational for mutating also Stk11 and RnaseL is revealed in line 9 page 13 and Figure S3.: The mice developed tumors more rapidly. However, the contribution of Stk11 and RnaseL alterations to PFS is minimal compared to Pten, Trp53, and Rb1 (Figure S1). Repeating the analysis without the 2 affected cases will not reveal dramatic changes in PFS. This should be further emphasised in abstract, and discussion.

The survival does not change significantly when we exclude STK11 and RNASEL from the TCGA dataset, as only a few samples are mutated. The dataset from Nature Genetics in 2018 does not contain survival data, so we cannot test the impact of the loss of STK11 and RNASEL on a larger dataset. We have now added this information to the manuscript at lines 557-560.

3. Fig. S3: Data showing Pten, Rb1, p53 loss combined with loss of Kmt2c or Kmt2d is not shown and needs to be included. Data showing Pten loss alone with KMT2C loss is also not shown but referenced in the Discussion on Page 19.

We apologize for the mistake that appeared in Figure S3, and the correct data has now been included in Figure S4. The loss of only Pten in combination with Kmt2c has not been addressed by us but was published by the group of Lukas Kenner, and we have referenced their work in the discussion. In that study, they showed the presence of lung metastasis in 3 out of 10 mice at 18 months after tumor initiation. We have mentioned this in the discussion at lines 592-594.

4. Fig. 1E. Difficult to say if reduced indel frequency for RnaseL and STK11 is a consequence of reduced CRISPR cutting efficiencies or a biological consequence of loss of these genes having no or even a negative selective pressure in prostate cancer development. Indeed, RNASEL can also be amplified in human prostate cancer (fig. S1).

To address these possibilities, the authors should first show CRISPR indel frequency in vitro to assess cutting efficiency.

As indel frequency does not full equate with protein loss, the authors should show protein expression by IHC/IF or Western blots to confirm the degree of protein loss in vivo.

Finally, they should generate tumor models with just the deletion of Pten, p53, Rb1 and compare tumor onset and severity to 5g model to definitely assess role of RNASEL and STK11 loss in prostate cancer development.

We have now added a table in Figure S2 showing the indels in MEF cells in vitro. However, this data does not reflect the guide efficiency and was obtained with a plasmid containing puromycin selections. The data only confirms that the guide can induce mutations at the target site of each gene but does not truly reveal the efficiency of the guides. Currently, there is no good method to directly compare guide efficiency, but a selection will occur in the tumor for the most optimal mutation profile.

We have also conducted Western blotting for Trp53, Pten, Stk11, Rb1, Rnasel, and Odam in cell lines derived from the tumors. This was done to avoid interference from wildtype cells in the tumor milieu. Unfortunately, we have not been able to obtain a reliable antibody for mouse Kmt2c. The WB data has been included in the new Figure S2 and is described in the methods section at lines 207-209 and result at line 326.

Tumor progression in Pten, p53, Rb1 deficient samples has not been assessed. This has been previously published using classical genetic engineering mouse models (PMID: 28059767). Instead, we have data for mutating Pten, p53, Rb1 in combination with the loss of Kmt2c/d, which has been included in Figure S4. Here we observe a delay in tumor progression, indicating that the loss of Stk11 and RNaseL favors tumor progression. This has been mentioned in the results and discussion at lines 356-358 and 557-558.

5. Again comparing Figure 1E , 2E and 4A, one would notice that the frequency of indels in the 5g is high only in Stk11, and this remains high in 8g, at the same level of Kmt2c, Kmt2d and Zbtb16 in 8g. Moreover, the frequency of indels in 8g seems higher than in 5g. How can the authors rule out that is rather the differential efficiency of the vectors in generating mutations that can cause the different metastatic potential?

The difference in indel percentage between 5g and 8g may be due to variations in the tumor microenvironment. Although a clear difference in histology assessment was not observed, there were notable variations between samples. Nevertheless, we conducted a statistical analysis of the indel frequency between the 5g and 8g groups, but it was not significant for any of the target genes, including Stk11.

6. Fig. 3: Did the authors assess Copy number changes? For instance, MYC is commonly amplified and has been shown to drive metastasis. CNAs have been shown to be much more common in metastatic prostate cancer than mutations, which occur generally at a low frequency. That being said, the mutational rate in these metastases is unusually high. How does this compare to the mutational rate in the primary tumors? Comparing primary vs. metastatic tumors will be necessary to conclude that acquisition of additional mutations during tumor evolution is not a possible driver of metastasis in their model.

The potential amplification of the genome or a specific region of the genome has been discussed among the authors. The mouse genome is highly homogeneous with few single nucleotide polymorphisms (SNPs), which complicates the analysis. To assess the amplification of MYC, which is upregulated in the samples, we examined the reads for the genomic region containing MYC and compared them to the average reads. This analysis revealed no indications of MYC amplification, as the reads were very similar to the average reads. We have included this data in Figure S6 and mentioned the results in lines 506-508, along with additional details in the methods section at lines 258-260.

The second part of the question is similar to reviewer 2's query, and I've included the answer here with some modifications:

Creating multiple mutations by CRISPR has been shown to induce cell death or generate genomic instability. Therefore, we analyzed a subset of the metastatic samples by whole-genome sequencing to identify potential driver mutations that were not intended. Overall, we found approximately 1.1~1.9 mutations/Mb, which is comparable to human metastatic samples (1-3 mutations/Mb)

(added to lines 396-398). However, very few common mutations in the coding sequence were found, aside from the sgRNA targets (a total of 33 mutations in 4 samples). This indicates that CRISPR-induced mutations in the target genes were the primary drivers of the cancer. However, we did find Nupr1l (NUPR2 in humans) mutated in two of the four samples. NUPR2 is a positive regulator of ferroptosis, and it could potentially be a driver mutation. This is mentioned in the discussion at lines 569-576.

7. Overall: Because there are so many genes targeted in the 8g setting, it is difficult to determine the essential genetic alterations and transcriptional signatures promoting metastasis. The authors need to narrow down the essential TSG losses that are sufficient for primary tumor growth, castration resistance, and metastasis, and then analyze those samples transcriptionally to identify the essential genes and their targets necessary for metastasis. Given their findings, this would include p53, Pten, Rb1, and KMT2c, who's losses were present at high frequency in both primary and metastatic samples.

We agree with the reviewer that p53, Pten, Rb1, and Kmt2c seem to be the most crucial mutation drivers for PCa formation and metastasis. We focused our analysis on the three epigenetic factors and performed single knockouts in combination with the loss of the 5g. This directed us to analyze Kmt2c and identify novel genes that are dysregulated in both human and mouse PCa, as shown in Figure 5. Furthermore, we conducted RNAseq analysis between the 5g and 8g groups to identify transcriptional alterations that can facilitate metastasis formation. These findings were linked back to KMT2C in a human cell line setting, and we have provided comments on this at lines 588-595 in the discussion.

8. Fig. 1G. The authors should stain for AR expression and expression of CK5 and CK8. Based on p63 staining it appears that tumor cells start express high levels of basal markers in the 5g setting, suggesting basal differentiation. The authors should comment on this.

We have stained samples for AR, Ck5, and Ck8, as shown in Figures 4G and 4J, together with p63 staining included in Figures 1 and S5. We observe subareas with predominantly p63-positive tumor cells, but in general, we see p63-negative tumor cells in both 5g and 8g tumors. Furthermore, the RNAseq analysis does not indicate basal cell differentiation in 5g tumors when compared to 8g tumors (see Figure 4I). The data is discussed on lines 619-621 and mentioned in the results at lines 434-437.

9. The castration of mice (fig 2C-D) revealed quite a bit of variability in reducing tumor weight as compared to intact mice (Fig 2C and 1J) but the mice reached the humane endpoint at the same time as the intact mice (Fig 2D and 1H). Given the imperfect CRSPR/Cas9 activity (Fig 1E), it would be worth to examine the dependence of AR of such tumors. Comparing castration in 5g and 8g and staining these for AR and AR target genes would also be of relevance. Does this newly generated model depend on AR at all ? The IHC analysis in S4A present weak cytoplasmic staining, similar to what is stated about Fig 4G-H, but the staining intensity is not quantified in the various groups. higher magnification images of the staining should be provided. The dependency of these tumors from AR should be clarified. If not rather these factors being important in progression of PC to CRPC, since androgen deprivation (castration) is the main stay treatment for primary PC patients, this model rather represent a possible genetic make up for high risk prostate cancer prone to develop metastases.

Overall, mouse models with loss of PTEN have shown to overcome castration/androgen deprivation with continual proliferation (PMID: 14522255 and PMID: 16079851). Therefore, it was expected that the tumors in this model would show resistance, as the tumor is more aggressive compared to tumors with only the loss of Pten. It is worth noting that after castration, two mice in each group had smaller tumor weights, indicating a partial response to testosterone deprivation. When analyzing the indels in these samples, we did not find any differences among the target genes.

Interestingly, the RNAseq data also shows decreased expression of the AR signature in the tumor, with even less expression in the metastasis (Figure 4E). We have included the heatmap for the AR signature as a new supplementary Figure S9, together with a high-power magnification of AR staining. We believe this is more accurate than scoring the staining intensity, as we observe variation in staining intensity from sample to sample.

We conducted qPCR for three AR-regulated genes to address the dependency of AR in the model. Interestingly, only Klk4 was significantly decreased in castrated mice (see Figure S5 and lines 357-360). Together with the castration resistance and the decreased expression of AR-related genes in the tumors, we interpret the data as the tumors being partly dependent on AR. Considering the evidence from Fig 1j and Fig 2c, in which both groups had two mice that partly responded to castration, we assume that AR dependency has been present in some individuals.

This has been added to the discussion, where we speculate that the majority of tumors in our models belong to “double negative prostate cancer”. This is stated at line 580-582: **A decrease in AR signature with no obvious upregulation of NEPC scores suggested these tumors belong to a subtype of aggressive PCa, identified as double-negative prostate cancer (DNPC), which is either AR or neuroendocrine driven with ability of therapy resistance.**

10. Figure 3 and Figure S5 deserves a little more attention. Figure 3F shows that additional mutations were found in all 4 WGsequenced samples but how can the authors exclude that those were not resulting from improper activity of the CRSPR/Cas9 and based on this, how can they attribute the increased metastatic potential in 8g only to the targeted 3 mutated genes? Did I read this correctly that none of the 4 sequenced tumors had mutations in ZBT16 (line 8-9 in page 14). This should be further clarified in the discussion (line 19-31 in page 19) and perhaps more attention should be given to the additional mutations found in the samples.

We performed whole-genome sequencing (WGS) to identify new somatic mutations but also to assess off-target mutations induced by sgRNAs. For each guide, we evaluated mutations in the top off-targets predicted to be mutated. Among the 47 sites assessed, we found only one off-target incidence generated by the Trp53 guide. These data, together with the small number of somatic mutations, lead us to conclude that off-target mutations have not driven cancer progression (see Figure 3 and S6, and comments on lines 390-394 and 406-408).

For Zbtb16, none of the four metastases had a mutation in this gene. As pointed out by the reviewer, this is a strong indication that Zbtb16 is dispensable for metastasis formation in this scenario and may even hinder PCa growth in this context. This has now been mentioned in the discussion at lines 560-563.

11. Fig 3D and 4C: The increased mutational burden in 3D and induction in genes related to DNA repair and immune signaling pathways in 4C (IFN, TNF, IL6, IL2) suggest that AAV delivery of CRISPR

constructs and/or constitutive Cas9 expression in the prostate epithelium may induce DNA damage and inflammation that may indirectly lead to tumor progression and metastasis. The authors should comment on this possibility or perform additional experiments (for instance without AAV packaging or with transient AAV delivery of Cas9) to rule out these confounding factors as drivers of metastasis.

In this study, we have included multiple controls to assess if the method applied could induce bias. We used AAV particles containing non-targeting sgRNAs, and this did not alter the prostate epithelium (see lines 309-310). Additionally, we used AAV to generate other models of PCa, and these displayed milder phenotypes and longer latency, indicating that the strong phenotype seen in this study is not driven by AAV/Cas9 activation (see references: PMID: 33674748, PMID: 36139541). We also used AAV containing Cre expression without observing a phenotype related to the method (see reference: PMID: 33674748).

For the RNAseq data in Figure 4C, we used Cre-positive mice, resulting in prostate tissues with Cre, Cas9, and GFP expression (see line 263). This ensures that the upregulation of the inflammation pathway is not driven by Cas9 expression but is very likely cancer-dependent.

Regarding off-target mutations that could be introduced by Cas9, we only observed one off-target by the Trp53 guide, as mentioned in response to question 10.

12. Fig. 5: This is a quite exciting and impactful finding. The authors though could have alternatively or in addition also use CRISPR to deplete Odam and Cabs1 in vivo in 8g tumors using their AAV approach to validate.

We agree with the reviewer that it could be elegant to include guides targeting Odam and Cabs1 in the AAV vector. However, we did not take that approach due to the negative selection that would occur for clones with intact Odam and Cabs1 in metastasis, as we observed for Zbtb16. Therefore, we used the generated cell line from a lung metastasis to knock out Odam and Cabs1. This ensured that the cancer cells would be depleted for both targets via in vitro selection, and we could assess the impact on metastasis formation.

13. Fig. 8D: Expression of Odam and Cabs1 that the authors showed were drivers of metastasis in Fig. 5 are not enhanced in human metastatic disease. The authors should comment and discuss. Moreover, only a handful of DEGs between 5g and 8g are altered in human metastatic disease and are not associated with differences in overall survival (Fig. S9b). Again, the authors should narrow down the list of genes and targets that are drivers of metastasis in their mouse model and then determine their relevance and correlation to human disease.

We thank the reviewer for raising this question, as we found a mistake related to Odam in Figure 8D. The data was re-analyzed, and a new heatmap figure was created by removing the non-essential genes. However, Cabs1 was not included because the microarray probe for it was not found in the dataset.

For the survival data, we tested the genes that we found differentially expressed between the 5g and 8g groups. Since 8g tumors can form metastasis, this could influence the survival of the patients. However, there are some limitations because not all of the genes can be detected in the available public dataset. As the reviewer mentioned, not all the differentially expressed genes (DEGs) will be associated with metastasis formation, but we have not fully tested that and cannot exclude genes

from the list. The TCGA dataset has few deceased patients, compromising the analysis. Therefore, we have now analyzed the relevance of these signature genes to survival in another dataset (GSE16560, PMID: 20233430). Here, we observe a significantly decreased survival for patients with higher gene expression of the gene panel. Again, this panel is a subset of the whole 24-genes derived from 8g vs. 5g primary tumors. This new figure has now been added to Figure S16, and comments at lines 531-539.

minor comments

1. fig S6 the gene labels are very small.

We have split the figure into two parts, thereby increasing the font size for the heatmap. See new Figure S8 for details.

2. the word “progression" in the paper is mentioned over 40 times to indicate that a primary untreated tumor develops metastasis. this is a little confusing if one thinks that disease progression follows emergence of treatment resistance in the clinical setting. The word progression should be changed of carefully defined as the onset of metastatic disease. e.g. lines 29-31.

We have carefully gone through the manuscript and changed 'progression' to 'onset of metastatic disease' where it is appropriate. We thank the reviewer for pointing this out.

3. what is the frequecy of indels in chr5qE1?

There are not many indels in the region when analyzing the coding gene located at chr4q13.3 (mouse chr5qE1) in TCGA. Close to 1% of patients had deep deletion or amplification. These alterations are nearly in the same patients, which may deserve further focus in the future.

4. figure 7: is the frequency of indels for g5+sgKmt2c higher than for g5+sgKmt2d and g5+sgZbtb16? this should be shown in a supplementary figure.

We have included a new graph in Figure 7E, where we show indels for Kmt2c, Kmt2d, and Zbtb16 in the primary tumors. We did not observe any difference in the indel frequency for the three genes.

5. fig 8c labels are not readable.

We have added a high-resolution heatmap to Figure S15, allowing zooming to read the gene names.

6. Fig. 1B: Unclear if one or both anterior lobes are injected. Can the authors comment on why disease also forms in DLVP if injections occur in anterior lobe?

The injection into the anterior lobe is feasible, and we injected into both lobes. The virus is distributed to the other lobes (see Riedel et al for details, PMID: 33674748), and we clearly observe high-grade PIN and GFP signal from the dorsolateral lobe at 4 weeks after injection. At the humane

endpoint, the prostate has lost its structure, but a large tumor mass is seen around the dorsolateral lobe. We have now added this information to the methods section at lines 179-180.

7. Figure 8E: what is the mutational status of tumors in TCGA PRAD scoring high (or the highest quartile) in respect to Kmt2c homologue? can the authors find any correlation?

We thank the reviewer for raising these questions. As suggested by the reviewer, we investigated the correlation between the high/low score and the mutation status of the eight genes (8g). As shown in the figure below, indeed, we observed a positive correlation, in terms of the signature high group of patients having a high frequency of mutations in 8g ($p = 0.025$, hypergeometric test). Among the eight genes we studied, only KMT2C is marginally significant ($p = 0.089$, hypergeometric test) with a higher mutation frequency in the high signature group of patients. These results suggest consistency between the mouse 8g tumor and human PRAD tumors with mutations in these 8 genes. Data has been added to Figure S16 and at lines 533-538.

8. FigS3 is missing G-I?

We apologize for this mistake as it is also related to the first question raised by this reviewer. The data is now present in figure S4.

9. Fig.4A: “proportion” not propotion

We thank the reviewer for this comment and have now corrected the figure.

10. Page 17, line 6: Should be KMT2C, not KMT2D

We thank the reviewer for this comment and have now corrected the text.

11. Some minor spelling/grammatical errors throughout.

We proofread the manuscript and have corrected mistakes throughout.

REVIEWERS' COMMENTS

Reviewer #1 (Remarks to the Author):

The authors have successfully addressed most of my reviewing comments. However, a significant concern remains regarding the rationale behind including Stk11 and RnaseL in their study:
While the authors cited another dataset showing higher incidence of somatic mutations in STK11 (4%) and RNaseL (2.4%), the authors also tried to indicate that the loss of Stk11 and RNaseL promotes cancer development in their model by showing their results comparing the humane endpoint between mice injected with AAV particles targeting 5 genes (Pten, Trp53, Rb1, Kmt2c and Kmt2d) and those injected with AAV particles targeting 8 genes (Pten, Trp53, Rb1, Rnase1, Stk11, Zbtb16, Kmt2c, and Kmt2d), as shown in Figure S4G. Notably, mice injected with AAV particles targeting 8 genes developed tumors significantly faster than those injected with AAV particles targeting 5 genes. However, it is inappropriate to conclusively attribute cancer development solely to the loss of Stk11 and RnaseL, as the observed effect could also be influences by the loss of Zbtb16. Therefore, I recommend that the authors rephrase their conclusions regarding the significance of Stk11 and RnaseL in promoting cancer development, unless they can introduce another cohort of mice injected with AAV particles targeting only 3 genes (Pten, Trp53, Rb1) to specifically assess the significance of Stk11 and RnaseL in this context.
Additionally, I also recommend that the authors include the NGS results they referenced in their response to my reviewing comments for a more comprehensive and transparent presentation of their findings.

Reviewer #2 (Remarks to the Author):

happy to accept. well done

Reviewer #3 (Remarks to the Author):

The reviewer is satisfied with the authors’ responses to the comments and additions to the manuscript.

RESPONSE TO REVIEWERS' COMMENTS

We would like to thank the Referees for their revision of our work, which has improved our manuscript. Overall, for this revision, we obtained data for mice with the loss of Pten, Trp53, and Rb1, which we have added to Figure S4. See details below:

Reviewer #1 (Remarks to the Author):

The authors have successfully addressed most of my reviewing comments. However, a significant concern remains regarding the rationale behind including Stk11 and RnaseL in their study:

While the authors cited another dataset showing higher incidence of somatic mutations in STK11 (4%) and RNaseL (2.4%), the authors also tried to indicate that the loss of Stk11 and RNaseL promotes cancer development in their model by showing their results comparing the humane endpoint between mice injected with AAV particles targeting 5 genes (Pten, Trp53, Rb1, Kmt2c and Kmt2d) and those injected with AAV particles targeting 8 genes (Pten, Trp53, Rb1, Rnase1, Stk11, Zbtb16, Kmt2c, and Kmt2d), as shown in Figure S4G. Notably, mice injected with AAV particles targeting 8 genes developed tumors significantly faster than those injected with AAV particles targeting 5 genes. However, it is inappropriate to conclusively attribute cancer development solely to the loss of Stk11 and RnaseL, as the observed effect could also be influences by the loss of Zbtb16. Therefore, I recommend that the authors rephrase their conclusions regarding the significance of Stk11 and RnaseL in promoting cancer development, unless they can introduce another cohort of mice injected with AAV particles targeting only 3 genes (Pten, Trp53, Rb1) to specifically assess the significance of Stk11 and RnaseL in this context.

We agree with the reviewer that the loss of Kmt2c and Kmt2d can complicate the interpretation of these data. We have obtained data from mice with the loss of only Pten, Trp53, and Rb1 by CRISPR in the mouse prostate. Here we observe a delayed phenotype and a humane endpoint around 3 months after induction due to large tumors. These data are in agreement with previously published data with conditional loss of these three factors in the mouse prostate epithelium. The data also suggest that the loss of Stk11 and RnaseL is accelerating prostate cancer progression, as mice with the loss of these two factors in combination with the loss of Pten, Trp53, and Rb1 reach a humane endpoint 2 months after cancer induction. We have included the data in Figure S4 by replacing the data for mice with the loss of Pten, Trp53, Rb1, Kmt2c, and Kmt2d.

Additionally, I also recommend that the authors include the NGS results they referenced in their response to my reviewing comments for a more comprehensive and transparent presentation of their findings.

We have not been able to fulfill this suggestion as it will require multiple optimizations before the assay for the specific target genes will be reliable.

Reviewer #2 (Remarks to the Author):

happy to accept. well done

Reviewer #3 (Remarks to the Author):

The reviewer is satisfied with the authors’ responses to the comments and additions to the manuscript.
