# Supplementary material for: CRISPR/Cas9 model of prostate cancer identifies Kmt2c deficiency as a metastatic driver by Odam/Cabs1 gene cluster expression
Source: Nat Commun. 2024 Mar 7;15:2088. doi: 10.1038/s41467-024-46370-0 (PMC10920892; doi:10.1038/s41467-024-46370-0)
Supplement: Supplementary file 2 — Peer Review File [file 41467_2024_46370_MOESM2_ESM.pdf]

nature portfolio

Corresponding author(s): Martin K. Thomsen

Last updated by author(s): Feb 6, 2024

Reporting Summary

Nature Portfolio wishes to improve the reproducibility of the work that we publish. This form provides structure for consistency and transparency in reporting. For further information on Nature Portfolio policies, see our Editorial Policies and the Editorial Policy Checklist.

Statistics

For all statistical analyses, confirm that the following items are present in the figure legend, table legend, main text, or Methods section.

n/a	Confirmed	
☐	☒	The exact sample size (*n*) for each experimental group/condition, given as a discrete number and unit of measurement
☐	☒	A statement on whether measurements were taken from distinct samples or whether the same sample was measured repeatedly
☐	☒	The statistical test(s) used AND whether they are one- or two-sided<br>*Only common tests should be described solely by name; describe more complex techniques in the Methods section.*
☐	☒	A description of all covariates tested
☐	☒	A description of any assumptions or corrections, such as tests of normality and adjustment for multiple comparisons
☐	☒	A full description of the statistical parameters including central tendency (e.g. means) or other basic estimates (e.g. regression coefficient) AND variation (e.g. standard deviation) or associated estimates of uncertainty (e.g. confidence intervals)
☐	☒	For null hypothesis testing, the test statistic (e.g. *F*, *t*, *r*) with confidence intervals, effect sizes, degrees of freedom and *P* value noted<br>*Give P values as exact values whenever suitable.*
☒	☐	For Bayesian analysis, information on the choice of priors and Markov chain Monte Carlo settings
☒	☐	For hierarchical and complex designs, identification of the appropriate level for tests and full reporting of outcomes
☒	☐	Estimates of effect sizes (e.g. Cohen's *d*, Pearson's *r*), indicating how they were calculated

Our web collection on statistics for biologists contains articles on many of the points above.

Software and code

Policy information about availability of computer code

Data collection	No software was used
Data analysis	R, Python, GATK, STAR, DESeq2, FeatureCounts, Gencode, GSEA and Prism was used for this study

For manuscripts utilizing custom algorithms or software that are central to the research but not yet described in published literature, software must be made available to editors and reviewers. We strongly encourage code deposition in a community repository (e.g. GitHub). See the Nature Portfolio guidelines for submitting code & software for further information.

Data

Policy information about availability of data

All manuscripts must include a data availability statement. This statement should provide the following information, where applicable:

- Accession codes, unique identifiers, or web links for publicly available datasets
- A description of any restrictions on data availability
- For clinical datasets or third party data, please ensure that the statement adheres to our policy

https://www.ncbi.nlm.nih.gov/bioproject/PRJNA1053864
https://www.ncbi.nlm.nih.gov/bioproject/PRJNA1061795

Research involving human participants, their data, or biological material

Policy information about studies with human participants or human data. See also policy information about sex, gender (identity/presentation), and sexual orientation and race, ethnicity and racism.

Reporting on sex and gender	n/a
Reporting on race, ethnicity, or other socially relevant groupings	n/a
Population characteristics	n/a
Recruitment	n/a
Ethics oversight	n/a

Note that full information on the approval of the study protocol must also be provided in the manuscript.

Field-specific reporting

Please select the one below that is the best fit for your research. If you are not sure, read the appropriate sections before making your selection.

☒ Life sciences ☐ Behavioural & social sciences ☐ Ecological, evolutionary & environmental sciences

For a reference copy of the document with all sections, see nature.com/documents/nr-reporting-summary-flat.pdf

Life sciences study design

All studies must disclose on these points even when the disclosure is negative.

Sample size	A power calculation was conducted before experiments were started. Sample sizes are ranging from 3 to 10 individual samples.
Data exclusions	No data was excluded from the study.
Replication	All replications were successful.
Randomization	The study was based on different experimental groups and the allocation of mice to each group was randomize.
Blinding	Blinding was not possible as each group was predefined. The evaluation of the tumors by the pathologist was blinded.

Reporting for specific materials, systems and methods

We require information from authors about some types of materials, experimental systems and methods used in many studies. Here, indicate whether each material, system or method listed is relevant to your study. If you are not sure if a list item applies to your research, read the appropriate section before selecting a response.

Materials & experimental systems

n/a	Involved in the study	
☐	☒	Antibodies
☐	☒	Eukaryotic cell lines
☒	☐	Palaeontology and archaeology
☐	☒	Animals and other organisms
☒	☐	Clinical data
☒	☐	Dual use research of concern
☒	☐	Plants

Methods

n/a	Involved in the study	
☒	☐	ChIP-seq
☒	☐	Flow cytometry
☒	☐	MRI-based neuroimaging

Antibodies

Antibodies used	pAkt (CST, 4060), Ki67 (abcam, 16667), E-cadherin (BD Biosciences, 610181), Androgen receptor (Millipore, 06-680), Synaptophysin

Antibodies used	(Santa Cruz, sc177501), p63 (CST, 39692), Ck5 (BioLegend, 905501), Ck8 (BioLegend, 904801), Trp53 (CST, 2524), Pten (CST, 9188), pRb1 (sc102), Stk11(sc32245), Odam (16509-1-AP), pSrc (CST, 2101), pLyn (CST, 2731), Vinculin (Sigma, v9131)
Validation	Antibodies has been validated in KO cell clones or tissue.

Eukaryotic cell lines

Policy information about cell lines and Sex and Gender in Research

Cell line source(s)	BPH-1 cells. Human (male) prostate. Benign prostate hyperplasia.
Authentication	The cells was used without authentication.
Mycoplasma contamination	Tested negative for mycoplasma contamination.
Commonly misidentified lines (See ICLAC register)	n/a

Animals and other research organisms

Policy information about studies involving animals; ARRIVE guidelines recommended for reporting animal research, and Sex and Gender in Research

Laboratory animals	B6J.129(B6N)-Gt(ROSA)26Sortm1(CAG-cas9*,-EGFP)Fezh/J mice were purchased from Jackson Laboratories (catalog no. 26175) together with Pb-Cre4 mouse line (catalog no. 68167).
Wild animals	n/a
Reporting on sex	Only male mice were used for studying prostate cancer
Field-collected samples	n/a
Ethics oversight	The mice were bred and housed at Aarhus University and all animal experiments were conducted in accordance with the protocol approved by the Danish Animal Experiments Inspectorate (license no. 2020-15-0201-00711).

Note that full information on the approval of the study protocol must also be provided in the manuscript.

Plants

Seed stocks	n/a
Novel plant genotypes	n/a
Authentication	n/a
